# Dual-informant clinical outcome assessment in the inclusive orchestra: measurement architecture for neurodevelopmental disorders prior to empirical evaluation

**DOI:** 10.3389/fneur.2026.1834441

**Published:** 2026-09-29

**Authors:** Tommaso Liuzzi, Fiammetta D’Arienzo, Susanna Staccioli, Sarah Bompard, Martina Frascari Diotallevi, Roberto Giuliani, Donatella Lettori, Enrico Castelli

**Affiliations:** 1 Unit of Neurorehabilitation, Bambino Gesù Children’s Hospital, IRCCS, Rome, Italy; 2 Santa Cecilia Conservatory of Music, Rome, Italy; 3 Euterpe APS Cultural Association, Rome, Italy; 4 Neurorehabilitation Research Area, Bambino Gesù Children’s Hospital, IRCCS, Rome, Italy

**Keywords:** clinical outcome assessment, clinician-reported outcome, dual-informant assessment, Euterpe method, neurodevelopmental disorders, observer-reported outcome, orchestral music therapy, systematic behavioral observation

## Abstract

**Background:**

Systematic behavioral observation in a group requires the rater to know when a target behavior could have occurred. In an inclusive orchestra the notated part and the conductor’s cues generate that occasion outside the participant, before the session. This pre-implementation study builds a dual-informant clinical outcome assessment architecture on that property, for adolescents and adults with neurodevelopmental disorders.

**Methods:**

The architecture comprises two context-specific candidate instruments, each of five seven-item domains. Working independently, two psychologists will score the same replayable 20-min orchestral segment within 24 h using the clinician-reported instrument (ClinRO-T), while a nominated caregiver completes the observer-reported instrument (ObsRO-P) for daily-life behavior directly observed during the subsequent 48 h. Paired items share an observable referent, and their correspondence is functional. An item-level grid predefines each behavioral referent, the event or window that creates a scoring opportunity, and the codes for structural inapplicability, unobservability and technical failure. A focal-participant rotation will yield 100 participant-session observations from 20 participants over 20 weeks, each independently double-coded in the clinician channel. A modified Delphi process and cognitive interviewing will precede form freeze. The first observational phase will report window adherence, coding burden, code distribution, inter-rater agreement at count, band and domain level, and the suitability of the Vineland Adaptive Behavior Scales as an external interpretive reference.

**Conclusion:**

The design preserves separate orchestral-session and daily-life assessment channels. Both instruments remain candidate clinical outcome assessments. No participants have been enrolled, no psychometric data are reported, and no measurement property is claimed as established.

## 1 Introduction

### 1.1 Neurodevelopmental disorders and neurofunctional targets

Neurodevelopmental disorders (NDD), as defined in the *Diagnostic and Statistical Manual of Mental Disorders, Fifth Edition* (DSM-5-TR), are heterogeneous early-onset conditions affecting cognitive, motor, linguistic and socio-communicative functioning (1). Connectivity research and network, integrative, and transdiagnostic accounts suggest that behavioral dimensions interact and overlap across NDD, cutting across diagnostic categories (2–5). An instrument sampling one domain in isolation may attribute behavior arising from interactions between domains to a single domain. Five domains are consequently observed on the same occasion, preserving cross-domain co-occurrence without assuming domain independence.

Observation in a clinically relevant context increases contextual resemblance to daily life. Resemblance alone does not establish ecological validity, which requires explicit operationalization and evidence linking assessment performance to real-world functioning (6). Feasibility and inter-rater agreement will be evaluated prospectively.

The term *neurofunctional* denotes a level of description — domains of functional organization operationalized through externally observable behavior — and not neural function. No neuroscientific finding is offered as evidence for the domain structure.

This pre-implementation methods study reports no psychometric results and prespecifies the measurement architecture required for subsequent empirical evaluation: dual-informant instruments, observation windows, rules for handling structurally inapplicable, unobserved or technically unavailable items, scoring, training and governance. The design draws on established frameworks for clinician- and observer-reported outcome measures (7–9) and on current regulatory guidance for fit-for-purpose clinical outcome assessments, whose appendices address observer- and clinician-reported measures specifically (10).

### 1.2 Music-based neurohabilitation and the Euterpe method

The clinical evidence base constrains what may be claimed for the program. A Cochrane review of music therapy in autistic people found moderate-certainty evidence for global improvement and reduced total symptom severity, and a small improvement in quality of life. Effects on social interaction and on communication were unclear, certainty being low or very low. The review did not support conclusions beyond young adulthood (11). That evidence base does not cover the whole of the anticipated recruitment pool, approximately 14–32 years, so the Socio–Communicative and Synchronization domains are treated as observational targets and not as expected outcomes. The review’s call for measures of behavior outside the therapy context also supports placing the caregiver channel in daily life.

The program is delivered face to face at a non-residential community association. The Euterpe Method organizes inclusive orchestral activity through standardized procedural algorithms (12). Earlier conceptual work described such settings as structured observational environments for functional behavior in NDD (13).

The present architecture comprises two context-specific candidate clinical outcome assessments: a clinician-reported instrument for recorded therapy-session behavior (ClinRO-T) and a caregiver-completed observer-reported instrument for directly observed daily-life behavior (ObsRO-P). The instruments have separate response processes.

The setting is itself a source of methodological structure (Figure 1). Entries, dynamic and tempo changes and section alternations are written in the part and cued by the conductor, so the occasion for a target behavior is generated outside the participant and can be fixed before the session. The conductor’s gesture is a standardized, repeatable stimulus inside a naturalistic context.

**Figure 1:**
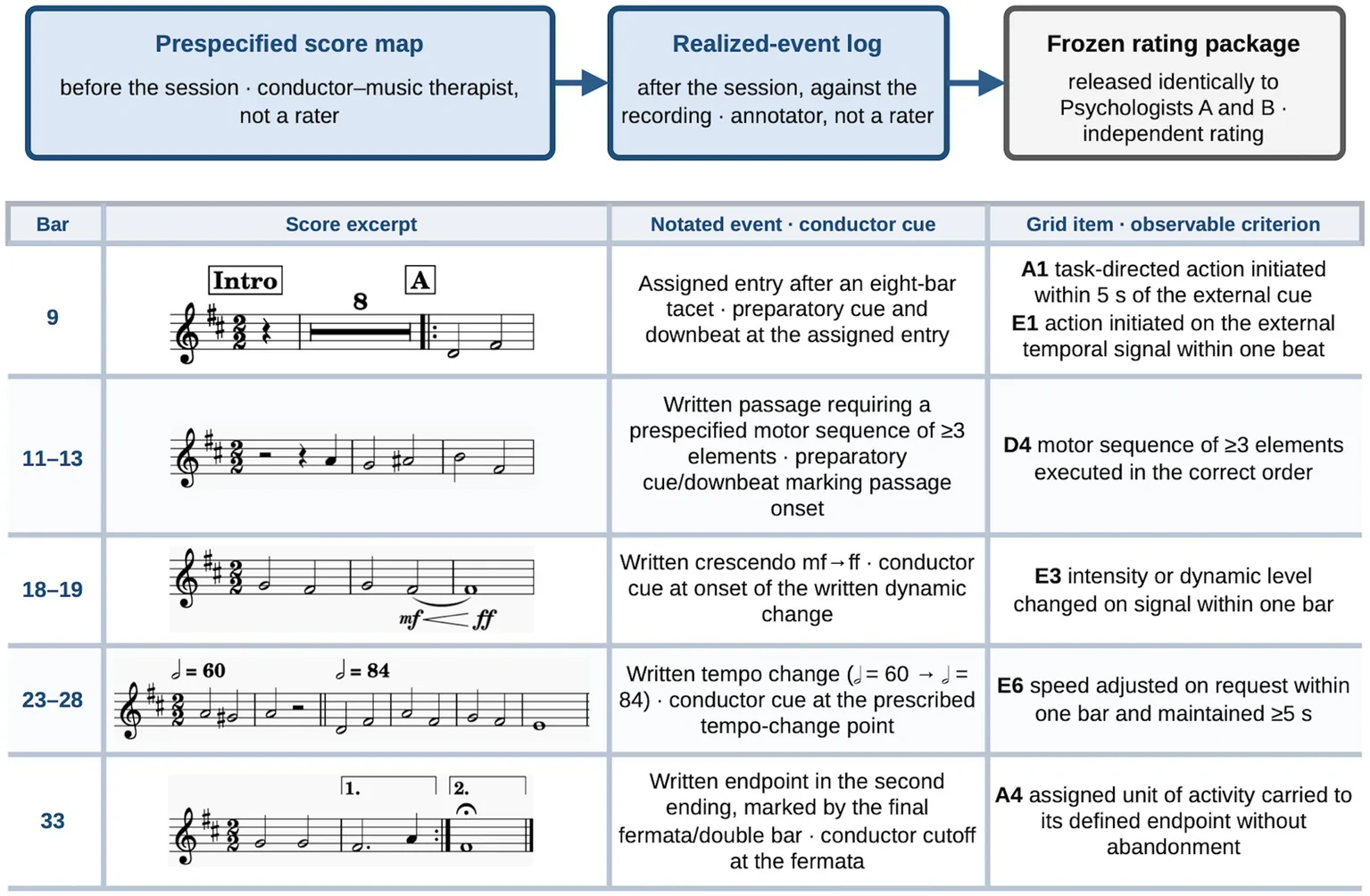
Score-generated observation opportunities: partial worked example on a participant’s assigned part. Five notated locations map to six of the 35 grid items. The example is restricted to opportunities generated by the score and is not representative of the grid. Domain C is absent. Endogenous opportunities — Those arising from an unplanned event, such as the audible error that opens A5, the detected loss of synchrony that opens E4, and the episode counts of item 7 — have no presession antecedent. The score map fixes the planned eliciting event before the session. The realized-event log records, against the recording, which planned events were realized, omitted, repeated or deviated. Both layers are frozen before rating and neither is produced by a behavioral rater. Prespecification concerns the eliciting opportunity and its observable criterion, not the participant’s response. The observable referent, the eliciting opportunity in each channel and the item-specific boundary and exclusion rules are given in Supplementary material S5. The excerpts are drawn from an inclusive arrangement, by one of the authors, of a work in the public domain.

Ensemble heterogeneity is a constitutive property of the setting. Synchronization and integration are defined only in the presence of competent partners, which is why the individual session is a site of acquisition and the group session the site of measurement. The same argument delimits transportability: the architecture applies wherever the scoring occasion is generated by an external temporal constraint shared by a group (other inclusive ensembles, group rehabilitation formats with a fixed temporal structure), and not to unstructured or dyadic settings.

### 1.3 Gaps in context-based outcome measurement

Three limitations recur in context-based assessment, and each is addressed by a specific component of the design. Domain specification during item development is often incomplete. The observation grid fixes, item by item, the observable referent and the event creating a scoring opportunity. Cross-informant agreement between professional and caregiver reporters varies, and has been reported as poor for challenging behaviors and moderate for social communication in preschool autistic children (14). The two channels are for this reason specified as separate instruments with independent reporting paths. Reporting windows often permit retrospective aggregation, and both channels operate under prespecified temporal boundaries. Analogous concerns arise in technology-based cognitive assessment in severe mental illness, where feasibility, reliability and ecological validity require empirical evaluation rather than inference from digital delivery (15). Session-based observation adds a condition of its own, since the sampling rule must be prespecified and grounded in systematic-observation principles (16, 17).

ClinRO-T ratings are completed from the recorded segment within 24 h of the orchestral music therapy (OMT) session. ObsRO-P covers daily-life behavior directly observed during the subsequent 48 h. Neither window fully excludes retrospective aggregation, which is why the design draws on an existing precedent in autism measurement: the caregiver-reported Observable Behaviors of Autism Spectrum Disorder Scale was developed around directly observable behaviors using a past-24-h recall period in children aged 4–12 years (18). That precedent supports bounded observer reporting in principle, not the specific 48-h window adopted here.

### 1.4 Rationale for a dual-informant clinical outcome assessment system

The observation context determines the use of clinician and caregiver reports. Experience-sampling procedures have been applied with autistic participants, but that evidence is concentrated in young and middle-aged adults with average or above-average intelligence and language, and does not extend to the heterogeneous NDD population targeted here (19). During ensemble performance the participant’s hands are occupied with the instrument and visual attention is divided between score and conductor, so an in-session prompt would interrupt the behavior under observation. Reporting is therefore assigned to sources with direct access to the relevant context. A psychologist codes the recorded session segment using prespecified rules, yielding a ClinRO rating (8), while a caregiver reports daily-life behavior from direct observation, yielding an ObsRO (9).

The instruments are designated ClinRO-T and ObsRO-P. Together they constitute a dual-informant clinical outcome assessment (COA), that is, two context-specific instruments rather than a single instrument completed by two raters (Table 1). The suffixes mark observation context, T the therapy session and P the personal daily-life setting, and carry no reference to reporter profession. ClinRO-T draws on recorded session evidence, whereas ObsRO-P draws on direct caregiver observation in daily life. Both channels instantiate the four-category COA taxonomy set out in the International Society for Pharmacoeconomics and Outcomes Research (ISPOR) Clinical Outcome Assessment framework (7), against which Table 2 classifies them. That framework targets criterion-anchored observation of externally defined behavior and does not extend to subjective experience or to participants’ own accounts.

**Table 1:** Defining properties of the prespecified dual-informant COA system.

Defining property	ClinRO-T	ObsRO-P
Reporter	Psychologists A and B, independently	One prespecified caregiver or regular support person
Evidence and sampling	Recorded 20-min OMT segment; prespecified phase and participant rotation	Direct observation in daily life; paired to the same participant and target week
Reference interval	One continuous 20-min segment	The 48 h after the scored OMT session
Completion boundary	Within 24 h after session end	By the end of the 48-h interval
Object of measurement	Externally observable behavior	Behaviorally analogous expression in a different context
Retrospective aggregation	Limited by replayable recorded evidence	Unavoidable within the interval
Observation density	Five prespecified observations per participant	Five paired reports per participant
Inference supported	Cohort-level characterization and feasibility	Exploratory cross-context correspondence only

ClinRO-T and ObsRO-P are paired by participant and study week but differ in reporter, source evidence and observational context. The pairing supports exploratory cross-context description; it does not imply metric equivalence, interchangeability or reliability between channels. ClinRO: Clinician-Reported Outcome; COA: Clinical Outcome Assessment; ObsRO: Observer-Reported Outcome.

**Table 2:** Classification of the two reporting channels against the ISPOR clinical-outcome-assessment taxonomy.

Attribute	ClinRO-T (therapy-session channel)	ObsRO-P (daily-life channel)
Who provides the report	Psychologist observing a standardized OMT segment	Caregiver observing daily life
Health-professional training required	Yes	No
Clinical judgment in applying the criteria	Yes	No — behaviorally anchored descriptors
Access to the patient’s internal / first-person states	No — external behavior only	No — external behavior only
Report of the patient’s own experience	No	No
Resulting COA class (ISPOR)	Clinician-Reported Outcome (ClinRO)	Observer-Reported Outcome (ObsRO)

The suffixes T and P denote the observation context (therapy vs. personal daily life), not the reporter’s role. For ClinRO-T, clinical judgment enters at the determination of observability and at the evaluation of the perceptual-musical criteria (on-beat entry, pulse maintenance, resynchronization, dynamic change), not at selection of the ordinal category, which is computed from the recorded counts.

### 1.5 Objectives and research questions

The primary objective is to prespecify, before empirical implementation, the operational structure and governance of the dual-informant COA system for adolescents and adults with NDD. Aggregate, non-study expressions of interest received by Euterpe APS, a social-promotion association, indicate an anticipated age span of approximately 14–32 years. This information is used for planning and does not constitute verified study data. Inclusion of adults follows from the persistence of neurodevelopmental conditions across the life course (20).

The primary phase 1 question is whether the system can be implemented in an inclusive orchestral setting. Five secondary questions will also be examined prospectively. The first is whether adherence to the ≤24-h ClinRO-T deadline and the 48-h ObsRO-P interval can be sustained, and what coding and form-entry durations are observed. The second is whether the five domains are interpretable and usable by raters and caregivers under naturalistic conditions. The third is whether the procedures keep the two channels independent. The fourth is whether the system supports secure, traceable and auditable data handling. The fifth is whether the prespecified use of the Vineland-3 (21) is suitable as an external interpretive reference. Each question is linked to a prespecified observable indicator for phase 1.

The five-domain model is fixed before data collection as the scoring structure. The planned observation density does not permit a test of structural validity or the derivation of an alternative structure, so phase 1 will provide only descriptive signals for later domain review under the prespecified revision conditions.

## 2 Materials and equipment

ClinRO-T and ObsRO-P are administered through separate digital channels. Technical specifications, item banks, scoring rules, non-observability pathways and governance procedures are documented in Supplementary materials S1–S12. Channel-specific temporal boundaries, timing assessment and deployment methods are summarized in Table 3.

**Table 3:** Administration parameters for ClinRO-T and ObsRO-P.

**Instrument**	**Temporal boundary**	**Deployment method**
ClinRO-T	Operational coding deadline: ≤24 h after session	Study-specific KoBoToolbox/Enketo link; configured controls will be subject to user-acceptance testing (22, 23)
ObsRO-P	48 h post-session daily-life observation interval; submit by interval end	Study-specific KoBoToolbox/Enketo link; configured controls will be subject to user-acceptance testing (22, 23)

The reporting windows are prespecified operational boundaries; their feasibility, burden and adequacy will be evaluated prospectively. Coding and form-entry duration is recorded without a fixed target or validity cutoff. Each scored OMT session generates one ClinRO-T form per designated participant. ClinRO-T: clinician-reported outcome, therapy-session channel; ObsRO-P: observer-reported outcome, caregiver channel; OMT: Orchestral Music Therapy.

### 2.1 Digital platform and electronic outcome-assessment configuration

The system will be configured on KoBoToolbox, an Open Data Kit (ODK)-based platform.

Marks and colleagues established the feasibility of ODK/Enketo for electronic research data capture, including timestamping, audit-related functionality and browser-based entry (22).

ClinRO-T and ObsRO-P will be deployed as distinct forms with separate submission links. Post-submission locking will be configured to prevent editing after submission. Before phase 1, user-acceptance testing will verify separation of the two reporting streams, locking behavior and display integrity across device classes (23), none of which has yet been carried out.

The metadata fields captured at submission and the quality-control variables derived from them are specified in Supplementary material S6. The observational evidence for ClinRO-T is a video recording of the OMT session. Because the grid applies criteria at the second level and to beat-level alignment, the recording configuration determines which opportunities are observable and therefore the denominators of items 1–6, and thus bears on measurement precision and not only on data capture.

Camera position and field of view relative to the designated targets and to the conductor, frame rate, and audio capture of the ensemble and of the conductor’s cues will be fixed before the first scored session. These parameters will be logged with each recording and verified against the calibration material during rater training, though no recording has yet been made for study purposes.

Items are presented on a five-point scale with three non-observability routes, Not Observed (NO), Structural Not Applicable (S-NA) and technical interruption/technical issue (TECH), which prevent forced responses. Caregiver onboarding is specified in Supplementary materials S2, S4.

### 2.2 COA instrument architecture

ClinRO-T and ObsRO-P each comprise five seven-item domains, for a total of 35 items per instrument. The domains are A, Cognitive–Executive; B, Socio–Communicative; C, Emotional–Motivational Regulation; D, Motor–Sensorimotor; and E, Synchronization, and an item is identified throughout by its domain letter and its position within the domain, so that A5 denotes the fifth item of the Cognitive–Executive domain. The 35 cross-channel item pairs use context-adapted behavioral referents whose correspondence is functional and not metric (Figure 2 and Table 4). In Domain E, the ObsRO-P items are daily-life functional analogues of ensemble synchronization, not identical behavioral referents.

**Figure 2:**
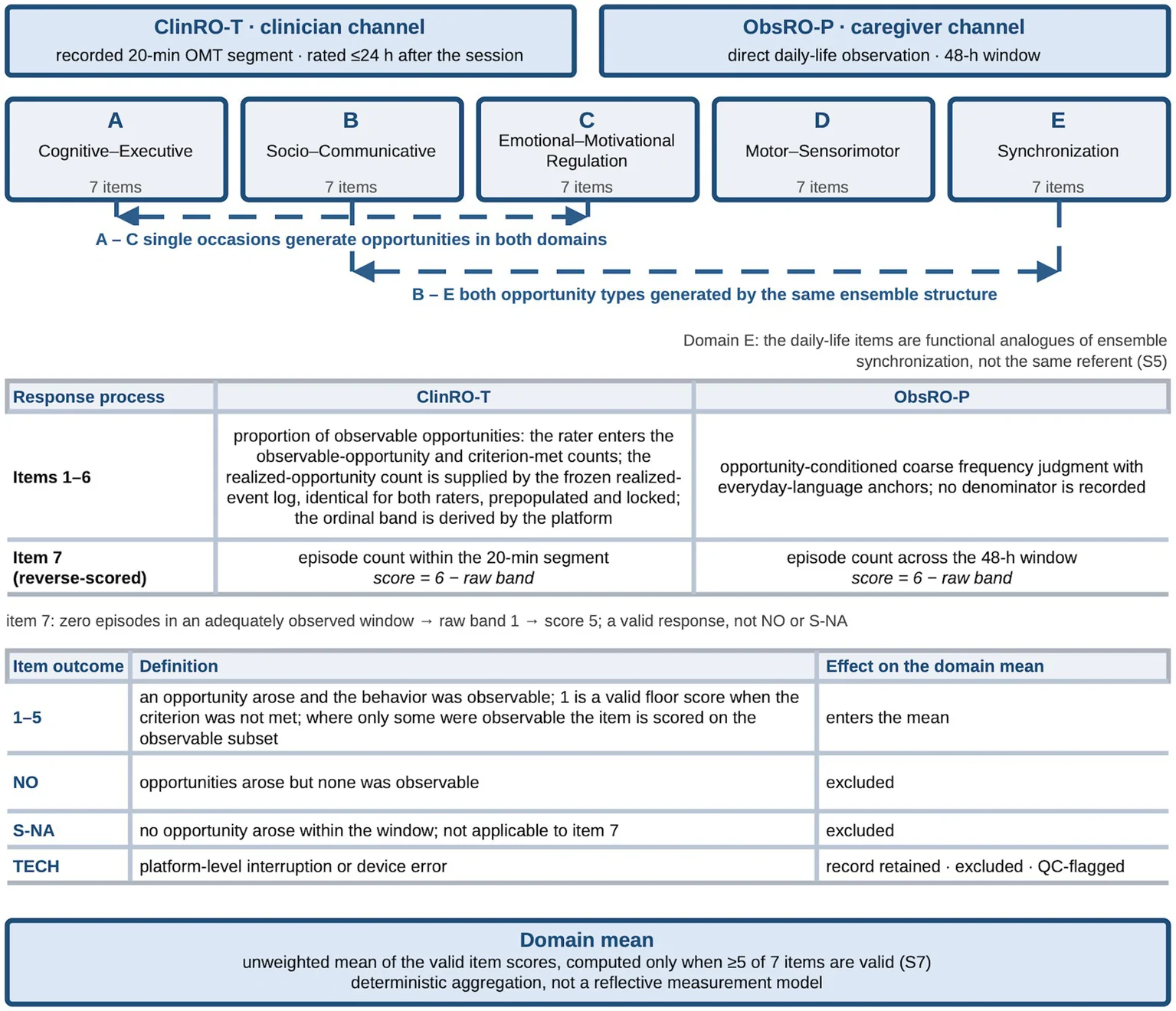
Domain and scoring architecture of the clinical outcome assessment system. The two channels share the item codes, the 1–5 ordinal range and the non-observability routes but differ in the response process by which the category is obtained. That difference bounds the interpretation of cross-context correspondence. Dashed connectors mark dependences arising in the scoring structure, not causal relations. Paired referents are functionally analogous and not metrically equivalent. The four item-level outcomes are exhaustive and mutually exclusive. A rating of 1 is a valid floor score and enters the domain mean, whereas NO, S-NA, and TECH yield no numeric contribution. Classifying non-occurrence as NO would therefore remove floor observations and bias the mean upward. The domain mean assumes rather than demonstrates exchangeability between a proportion and an episode count on a single ordinal scale, and implies no reflective measurement model. Item content, band boundaries, and aggregation logic are given in Supplementary materials S5, S7. ClinRO-T, clinician-reported outcome, therapy-session channel; ObsRO-P, observer-reported outcome, caregiver channel; NO, not observed; S-NA, structural not applicable; TECH, technical interruption; OMT, orchestral music therapy; QC, quality control.

**Table 4:** What the two channels measure, by domain: behaviorally analogous referents assessed in the orchestral session (ClinRO-T) and in daily life (ObsRO-P).

Domain	ClinRO-T — in the OMT session	ObsRO-P — in daily life
A Cognitive–Executive	begins on the conductor’s cue; sustains attention through a passage	Begins a requested task without the request being repeated
B Socio–Communicative	coordinates with section peers; takes turns in the ensemble	Engages appropriately with a familiar person
C Emotional–Motivational Regulation	recovers observably after a performance error	Recomposes after a small everyday frustration
D Motor–Sensorimotor	executes the required motor sequence on the instrument	Carries out an everyday motor task
E Synchronization	enters and stays synchronized with the ensemble	Keeps pace within a shared daily routine

Cross-context correspondence is exploratory and may be compatible with generalization but does not establish reliability, metric equivalence, an effect of OMT, or transfer.

## 3 Methods

### 3.1 Assessment protocol

The prospective assessment protocol is specified here, covering setting, sampling, rating, scoring, governance and ethics.

#### 3.1.1 Pre-implementation methodological phase

This manuscript describes the pre-implementation phase only. No participant was enrolled, no clinical data were collected and no psychometric estimate was generated. All materials were kept under version control.

#### 3.1.2 Setting and program

Sessions will be delivered face-to-face at Euterpe APS, an Italian nonprofit social promotion association operating in a non-residential community setting. Participants will attend the scheduled activity and return home. The prospective enrollment target is N = 20, and a typical OMT session will bring those participants together with 7 music therapists, 6 professional musicians, 6 volunteers and 1 clinical psychotherapist, some 40 people present. Non-probability mixed recruitment will combine spontaneous APS interest with the Bambino Gesù Children’s Hospital, IRCCS (OPBG), or the Azienda Sanitaria Locale (ASL; local health authority) Roma 4 referral. All sources will follow identical consent and eligibility procedures, source will be recorded and no diagnostic quota will apply.

Across the 20-week window each participant will follow 20 OMT and 20 individual music therapy (IMT) sessions, two per week and at least two days apart. IMT is the context in which individualized instrumental competencies are developed. OMT is the context in which their expression becomes observable within an inclusive ensemble. Daily-life observation examines whether behaviorally analogous expressions are reported outside OMT. Scoring is conducted only in OMT and in daily life, not in IMT. Cross-context correspondence is exploratory: it may be compatible with generalization, but establishes neither transfer nor an effect of OMT. The planned setting, program modules, weekly structure, cohort parameters, scored contexts, data medium and design status are summarized in Table 5.

**Table 5:** Setting and session structure of the planned phase 1 observational study.

**Feature**	**Specification**
Setting	Community-based, non-residential APS; participants attend the scheduled activity and return home
Program modules	IMT (individual, 60 min); OMT (orchestral group, 90 min); scoring occurs in OMT and in daily life only
Weekly structure	One IMT and one OMT session per week, scheduled at least two days apart; over 20 weeks, 20 IMT and 20 OMT sessions
Composition of the OMT session	Approximately 40 individuals per session: 20 enrolled participants meeting the prespecified NDD eligibility criteria, 7 music therapists, 6 professional musicians, 6 volunteers, and 1 clinical psychotherapist
Enrolled participants	*N* = 20, adopted for analytic homogeneity and ensemble balance
Age range	14–32 years; prespecified for the planned study from current anticipated APS demand and the adult eligibility pathways
Scored contexts	OMT (clinician channel) and daily life (caregiver channel). IMT is the site of acquisition and is not a measurement site
Data medium	Electronic capture (KoBoToolbox, Enketo/ODK)
Design status	Monitoring design: no comparison condition, and no attribution of observed change to any single program module

The parameters are prospective protocol specifications fixed before enrollment and without participant data. The development record for the instruments is given in Supplementary material S12. APS: Associazione di Promozione Sociale (social-promotion association); IMT: Individual Music Therapy; NDD: Neurodevelopmental Disorders; ODK: Open Data Kit; OMT: Orchestral Music Therapy.

#### 3.1.3 Planned phase 1 observational study

The prospective 20-week feasibility study targets N = 20 participants aged 14–32 years at consent. Eligibility requires a qualifying NDD consistent with DSM-5-TR criteria or International Classification of Diseases, Eleventh Revision diagnostic requirements (1, 24). The diagnosis must be source-documented and verified before enrollment by a clinician qualified in neurodevelopmental assessment. Verification will occur through OPBG for participants under 18, through a formally authorized OPBG transition pathway for adults previously managed there, or through ASL Roma 4 under a written inter-institutional agreement for other adults. Participants without a documented verification pathway will not be enrolled. If the available records are insufficient, clinical confirmation must be obtained through the applicable pathway. That pathway must also document whether 90-min inclusive OMT is safe with individualized accommodations and planned breaks. A syndromic or genetic diagnosis alone is insufficient, and no genetic or genotype analysis is planned. Cerebral palsy is treated as a neurological comorbidity and not as a qualifying NDD. A participant with cerebral palsy may enroll only if an intellectual disability or another documented NDD is present, cerebral palsy alone permitting ensemble participation but not study enrollment. Exclusion is limited to an acute or clinically unstable medical, neurological or psychiatric condition judged unsafe despite accommodation. Assistance, augmentative communication, adapted parts, scheduled breaks and lack of musical experience are not exclusions.

Age at consent, sex and gender identity will be recorded at enrollment and reported in aggregate following the Sex and Gender Equity in Research (SAGER) guidelines (25), together with diagnostic category, recruitment source and orchestral section. Domain and feasibility summaries will additionally be reported by age band (14–17 and 18–32 years) as descriptive strata, without inferential comparison.

At screening, the participant and caregiver must confirm anticipated availability for the 20-week program and for the five prespecified target weeks. Later absences or withdrawal will not trigger retrospective exclusion or invalidate prior observations, and are retained as adherence, retention and missing-data outcomes. A participant absent in a designated target week is not replaced, and the corresponding participant-session observation is recorded as missing, so that the realized total may fall below 100 participant-session observations.

#### 3.1.4 Sampling and target designation

Exhaustive participant-level coding of all enrolled participants at every session would require 400 potential participant-session observation units (20 participants × 20 sessions) and is not assumed feasible. Accordingly, the video-observation protocol uses a prespecified rotating focal-participant sampling scheme, adapted from established focal-subject sampling principles in which observation is centered on identified individuals over defined observation periods (16). The sampling frame, observational unit, target-selection rule, observation duration and coding procedure are specified prospectively, before any data are observed. This is consistent with contemporary recommendations for systematic coding of observed human behavior and with methodological work on sampling from video data (26, 27).

Five participants are designated for each scored session, and each is rated against the complete replayable 20-min OMT segment (T = 20 min), which is not subdivided among the five targets. Across 20 scored sessions, each of the 20 participants contributes five scored occasions, yielding 100 participant-session observation units and five focal participants per session (Table 6). The target-allocation schedule will be generated by the study statistician before the first scored session, stratified by orchestral section and frozen under version control. It provides five scored occasions per participant, and does not require pairwise balance in how often participants are co-designated.

**Table 6:** Sampling design and target-designation rule for the clinician channel.

**Parameter**	**Symbol**	**Value**	**Definition and rationale**
Planned target sample	N	20	Prospective recruitment target; no cohort is currently enrolled
Scored OMT sessions	S	20	One OMT session per week across the 20-week window
Observation segment	T	20 min	One continuous segment per session; across the 20 sessions: 7 opening (0–20 min), 6 middle (35–55 min), and 7 closing (70–90 min), interleaved across the twenty weeks rather than blocked
Coding and form-entry duration	C	Logged	Logged as a feasibility outcome; no fixed cutoff and not inferred from segment length
Targets per session	k	(N·m)/S = 5	Five targets per session distribute 100 records across 20 sessions
Observations per participant	m	5	Prespecified balanced rotation; each participant is observed in all three session phases, though necessarily in unequal number (Figure 3)
Total participant-session observations	O	N·m = S·k = 100	Twenty participants × five occasions = twenty sessions × five targets
Dual-channel rule	—	Symmetric	Both channels report on a participant only in that participant’s target week, yielding five paired clinician–caregiver observations per participant
Inference supported	—	Cohort level	Five observations per participant support cohort-level characterization and feasibility assessment; they do not support estimation of individual trajectories

Target designation is a prespecified rotation rather than an exhaustive census. Five targets per session are required by the prespecified balanced panel (k = m·N/S); the practical coding burden associated with this sampling choice is an empirical feasibility outcome and is not presented as a known rater-capacity limit. The number of targets is not a partition of the segment: Coding is carried out after the session from a replayable recording, and every target is coded against the full segment. ClinRO-T: clinician-reported outcome, therapy-session channel; OMT: Orchestral Music Therapy.

One 20-min segment is selected from each 90-min OMT session according to a temporal sampling schedule fixed before data collection. Segment selection is stratified across three prespecified within-session positions: seven opening segments (minutes 0–20), six middle segments (minutes 35–55) and seven closing segments (minutes 70–90). The three positions will be interleaved across the 20 study weeks so that session position is not tied to program chronology or cumulative exposure (Figure 3), keeping mean study week similar across the three strata. Each participant’s five scored occasions will include at least one observation from each position. Because five occasions cannot be divided equally among three positions, their representation is necessarily unequal. This design broadens temporal coverage but does not randomize session phase. Phase-specific summaries will therefore remain descriptive and will not be interpreted as causal or comparative phase effects.

**Figure 3:**
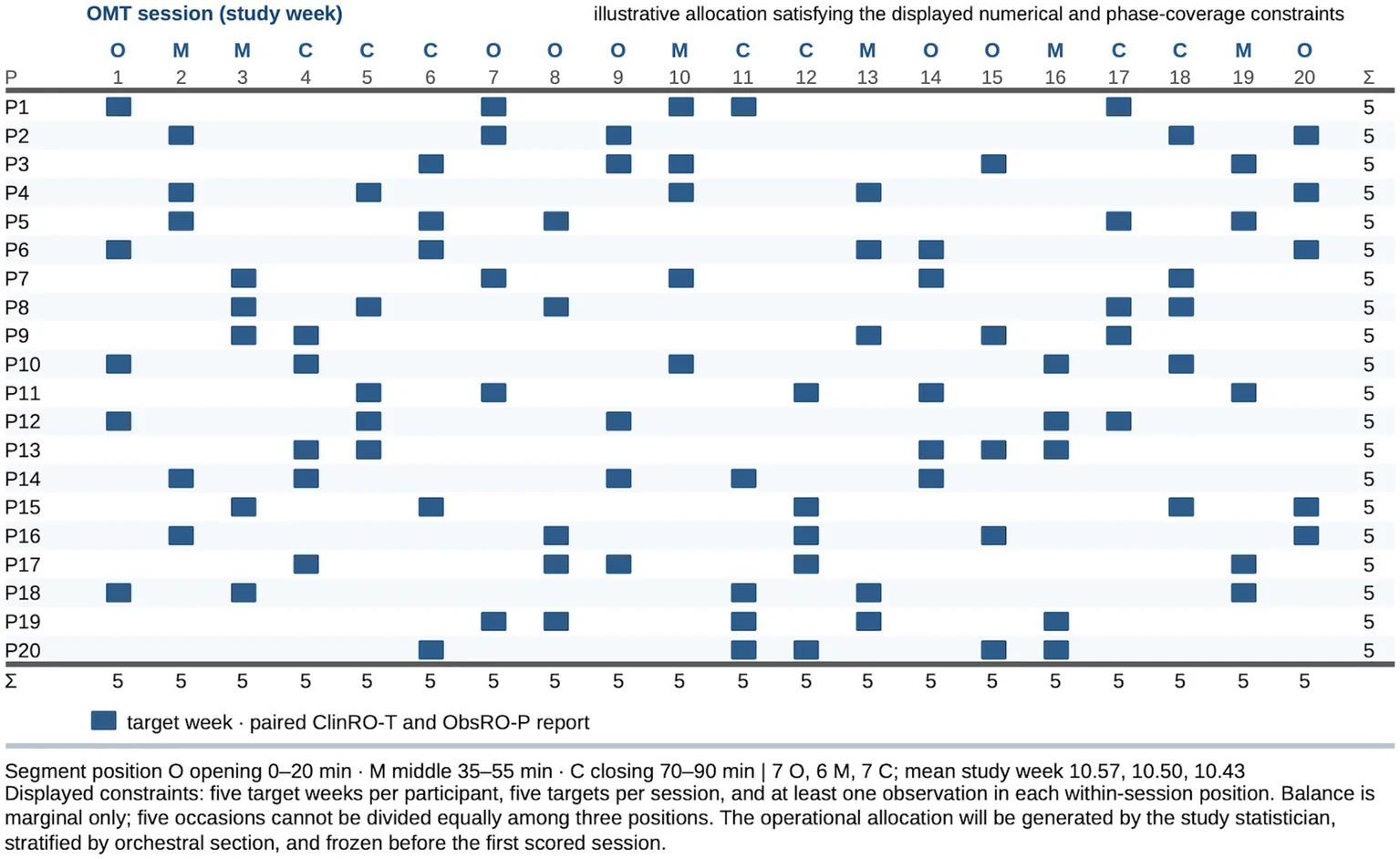
Architecture of the dual-informant monitoring system. The two branches are operationally separate: The daily-life branch does not derive from the recorded segment, and the caregiver has no access to ClinRO-T entries. Both channels report on a participant only in that participant’s target week. Psychologists A and B receive an identical frozen rating package, so their independent ratings concern equivalent procedures. Agreement between them is therefore conditional on a common identification of the scoring occasion and does not estimate the accuracy of that identification. The two comparison parameters are distinct and not interchangeable: Inter-rater agreement concerns the same evidence, whereas cross-context correspondence concerns different reporters and observational contexts and is exploratory. Masking conditions are given in Table 7. Masking to participant identity and program context is not claimed. The full sampling specification is given in Table 6, and capture and governance procedures in Supplementary materials S6, S11. IMT, individual music therapy; OMT, orchestral music therapy; ClinRO-T, clinician-reported outcome, therapy-session channel; ObsRO-P, observer-reported outcome, caregiver channel.

Observation-window duration is treated as a study-specific sampling parameter, because the duration and number of coded observation periods can affect the behavioral information captured and the reliability of video-based observational measures (28). The supporting evidence for the present 20-min window is convergent, drawn from other settings. Blinded video raters have obtained substantial agreement from a prespecified 15-min window in active music therapy with children with developmental disorders (29). Observation length has affected reliability and validity in other video-based systems, including studies using 20-min classroom coding occasions (30). Thin-slice and clinical group-interaction studies indicate that the amount of observation required depends on the construct, coding system and behavioral context (31, 32). The 20-min window is prespecified as the sampling unit and is not presented as a validated, optimal or psychometrically established cutoff for inclusive OMT. Its empirical adequacy will be evaluated prospectively using opportunity yield, phase- and domain-specific NO/S-NA frequencies, inter-rater agreement, coding burden and technical or operational missingness.

When a participant is designated for a given week, both informant channels report on that same participant. Neither channel produces a rating for participants who were not designated for that participant-session occasion. Non-designated participant-session combinations fall outside the planned observational sample; they are not classified as NO/S-NA and do not represent technical or operational missingness. Independent double-coding of ClinRO-T yields 200 coder-level records for inter-rater assessment, while the planned sample remains 100 participant-session observation units. Cross-channel correspondence is analyzed separately and is not interpreted as inter-rater reliability, because the two channels constitute distinct informant sources and not interchangeable ratings of the same measurement process.

#### 3.1.5 Rater training and calibration

Rater preparation follows a standardized observational framework supported by operational manuals and video-based calibration modules using 20-min excerpts. Comparable development work on video-based observational instruments in autistic children has incorporated explicit rater training, iterative item refinement and repeated examination of inter-rater agreement before interpretation of coded observations (33).

The calibration material will be simulated: the segments used for training and concordance review will be reconstructed for the purpose, informed by the conductor–music therapist’s prior experience of the program. They will contain no footage or data from patients, study participants or actual sessions. Individuals appearing in the simulated materials will provide authorization for recording and training use.

Consent procedures for the recording of real sessions, which phase 1 will require, are specified in Supplementary material S11 and will be submitted with the protocol for ethics review.

Agreement between the two raters on the calibration material will be computed before the first scored session and reported alongside the phase 1 results. Because the material is simulated and constructed to contain boundary cases, this is a check on training adequacy and on the discriminability of the anchors, and does not substitute for the phase 1 agreement analysis or provide a reliability estimate. Structured monitoring and periodic review of scoring patterns are specified in Supplementary material S3. Because digital literacy varies among caregivers, ObsRO-P onboarding includes an instructional video, a one-page reference sheet and a supervised practice submission before any naturalistic reporting begins. Caregivers receive procedural support only, with no guidance on how to score specific behaviors, so as to preserve response-process independence.

#### 3.1.6 Identification of scoring occasions

Identification of the scoring occasion is separated from the rating of behavior. For each scored work the conductor–music therapist, who is not a behavioral rater, will prepare a presession score map listing, by measure, the notated event, the conductor cue, the associated item code and the observable criterion. The map will be frozen and version-controlled before the session. After the session a musically competent annotator, likewise not a behavioral rater, will compile a realized-event log against the recording. It records the actual timestamp of each planned event, the cue as produced, whether the passage was executed, omitted or repeated, and any restart or deviation. The log records events only and assigns no rating. Both layers will be frozen before behavioral rating begins. Psychologists A and B will receive an identical package: the same video segment, score map, realized-event timestamps, criteria and temporal windows. Their independent ratings will therefore be based on the same evidence and procedures. Behavioral raters need not read the score or recognize repeats, cues or section turns. Musical judgment is confined to the non-rater annotation layer, which is produced once and not independently replicated.

The two layers reduce but do not remove the perceptual demand of some items. Judging whether an entry falls on the beat, whether pulse is maintained, whether resynchronization occurs or whether action is coordinated with the section still requires perceptual comparison against the ensemble. For these items the criterion is an explicit temporal tolerance relative to the conductor’s downbeat or cue, applied in place of an unaided judgment. Calibration uses material annotated by a musical expert. Whether raters without formal musical training reach adequate agreement on them is an empirical question for phase 1. Item-level agreement will be reported for each item and, secondarily, by observational-demand class.

#### 3.1.7 Role segregation and masking

Two rating roles are operationally separated. Psychologist A observes the session, completes ClinRO-T and administers the Vineland-3. Being present in the program context and distinct from the intervention provider, A cannot be masked to the setting. Psychologist B has no intervention or clinical-management role, does not attend the sessions and independently codes every scheduled ClinRO-T participant–session observation from the same pseudonymized video segment. Psychologist B is masked to A’s ratings, ObsRO-P reports, Vineland-3 results, clinical records, treatment objectives and previous assessments. Masking to participant identity and program context is not claimed because these may be inferable from the recording (Table 7 and Figure 4).

**Table 7:** Rater architecture and the two distinct comparison parameters.

Element	Specification
Primary rater	Psychologist A: session observer, distinct from the intervention provider (conductor–music therapist), present during the OMT session and not masked to program context
Independent secondary rater	Psychologist B: no intervention or clinical-management role; absent from live sessions; independently codes all scheduled participant–session observations; masked to A’s ratings, caregiver reports, Vineland-3 results, clinical records, treatment objectives and previous assessments; not claimed to be masked to identity or program context
Inter-rater agreement	Psychologist A versus Psychologist B on the same 20-min segment for all scheduled participant–session observations (target n = 100); item-level ordinal and domain-level agreement reported separately with repeated observations accounted for. Double rating yields two locked rater-specific records; no consensus, averaged or adjudicated score is derived
Cross-context correspondence	Each locked clinician rating versus the matched ObsRO-P record for the same participant and week, reported separately for Psychologist A and for Psychologist B; parallel exploratory description only, not pooled or averaged
Bias control for the session-exposed primary rater	Item-level anchoring to observable referents (Supplementary materials S1, S2), common-video anchoring and independent double-coding of all scheduled ClinRO-T observations; these procedures standardize the evidence available to the two raters and preserve independent records but do not eliminate expectancy or context-inference bias
Informant segregation	The clinician and caregiver reporting streams are operationally separated; neither informant has access to the entries of the other

Agreement between Psychologists A and B on the same recorded evidence is an inter-rater agreement parameter; correspondence between ClinRO-T and ObsRO-P concerns different reporters and observational contexts and is exploratory. ClinRO-T: clinician-reported outcome, therapy-session channel; ObsRO-P: observer-reported outcome, caregiver channel; OMT: Orchestral Music Therapy.

**Figure 4:**
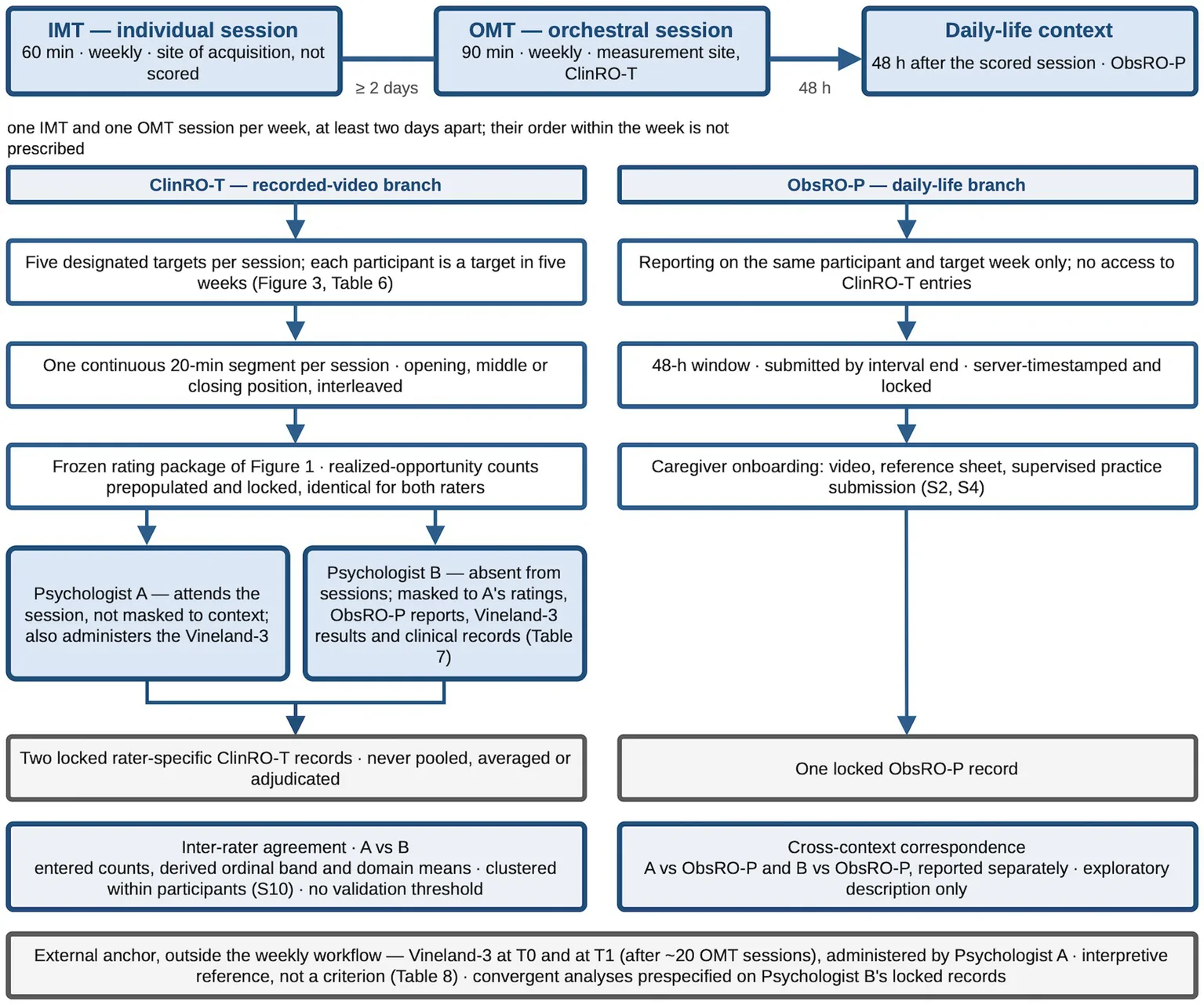
Planned sampling schedule: Illustrative target-participant allocation for paired ClinRO-T/ObsRO-P target weeks and ClinRO-T segment position across the 20 OMT sessions. The matrix is one allocation satisfying the numerical and phase-coverage constraints it displays: Equal participant-level replication of five target weeks, five targets per session, and at least one observation from each within-session position for every participant. Row and column sums are shown at the margins. Stratification by orchestral section is a further prespecified constraint that the display does not represent. The operational allocation will be generated by the study statistician before the first scored session, stratified by orchestral section, and frozen under version control, so P1–P20 are placeholders. Designation is shared by the two channels: A participant designated for a given week is reported by both, and a participant not designated is reported by neither. Segment position applies to the ClinRO-T segment only. Balance is marginal only: It does not extend to the frequency with which participants are co-designated within a session. Because five occasions cannot be divided equally among three positions, phase representation is unequal by construction, which is one reason phase-specific summaries remain descriptive. Segment position is interleaved rather than blocked, leaving it approximately orthogonal to study week and to cumulative exposure. The matrix neither represents nor supports an effect of segment position. ClinRO-T, clinician-reported outcome, therapy-session channel; ObsRO-P, observer-reported outcome, caregiver channel; OMT, orchestral music therapy.

The two raters use separate access paths and cannot view one another’s entries. Each rating is locked before the paired records are released for agreement analysis. Any discrepancy review occurs only after both ratings have been finalized and is appended without overwriting either entry. Double rating yields two locked rater-specific records, and no consensus, averaged or adjudicated score is derived. Any observation not independently coded by both raters according to the schedule in Figure 3 is documented as a protocol deviation and excluded from the primary agreement set. The clarification record is used only to examine sources of rating discrepancies and does not enter the analytical dataset.

#### 3.1.8 External anchoring

The Vineland-3 Interview Form will be administered at baseline (T0) and at completion of the 20-week phase 1 observation period, after approximately 20 OMT sessions (T1). It serves as an external interpretive reference for adaptive functioning over a broader reference frame (21). It is not a criterion against which the COA indicators are validated. Its role, timing and independence safeguards are specified in Supplementary material S9, and Table 8 states the conceptual non-equivalence between the two instruments. Administration will follow standardized procedures and scoring will be independently verified before entry into the dataset. Because Psychologist A both completes ClinRO-T and administers the Vineland-3, later convergent analyses are prespecified to use Psychologist B’s locked records, B being masked to the Vineland-3 results. The corresponding analyses on A’s records will be reported separately and identified as subject to common-rater variance.

**Table 8:** Conceptual comparison between the COA system and Vineland-3.

Feature	COA system	Vineland-3
Temporal window	Bounded observation intervals (20-min OMT segment; 48 h post-session daily-life interval)	Broader reference frame of typical behavior
Operational construct	Behavioral expression within a prespecified bounded interval	Standardized adaptive functioning
Scoring metric	Study-specific domain summaries	Norm-referenced standardized scores
Analytical role	Repeated session-level monitoring at cohort level	External adaptive-functioning characterization at T0 and T1

The two approaches operate over different reference frames and are complementary rather than interchangeable: The COA system records prespecified observable behavior within bounded observation intervals, whereas Vineland-3 provides a standardized characterization of adaptive functioning over a broader reference frame. The comparison is conceptual; the degree of empirical correspondence between the two is an open question for the observational phase. COA: Clinical Outcome Assessment; OMT: Orchestral Music Therapy; Vineland-3: Vineland Adaptive Behavior Scales, Third Edition.

#### 3.1.9 COA administration protocol

The two streams are operationally separated to maintain informant independence. ClinRO-T is completed by the ≤24-h post-session deadline from the recorded segment. That deadline is set to prevent backlog and cross-session contamination and is not a criterion of measurement validity. ObsRO-P covers behavior directly observed during the subsequent 48-h interval and is submitted by its end through a separate secure link (Table 3). The 48-h interval allows relevant opportunities to occur outside the orchestral setting. Informants rate only behavior directly observed within the applicable interval and do not use second-hand accounts. Absent opportunities or observations follow the S-NA and NO pathways. Submissions are server-timestamped and locked on completion. Completion latency and associated missingness are feasibility outcomes.

Before enrollment each participant will nominate one primary parent, caregiver or regular support person with direct observational access in daily life who agrees to complete the five scheduled forms. An adult participant must authorize that person’s involvement. The informant must have no role in OMT delivery, clinical supervision, outcome rating or program volunteering. The same informant will be retained across the five target weeks and will report without access to ClinRO-T entries. An unavoidable replacement requires the same onboarding, a new pseudonymous identifier and a documented informant-change flag. Affected records are retained and examined separately.

The five domains are operationalized through the observation grid (Supplementary material S5), which defines three elements for each of the 35 items: the observable behavioral referent constituting an occurrence; the eliciting opportunity in each channel, which provides the denominator for a frequency rating; and the boundary cases that invalidate an opportunity or a putative occurrence.

Items 1–6 are scored as the proportion of opportunities on which the criterion was met. In the clinician channel the denominator is the number of opportunities recorded from the passage in the score map. Three cases are distinguished at item level: no opportunity arose (S-NA); opportunities arose but none was observable (NO); or some were observable. In the third case the item is scored on the observable subset with both counts retained. The number of realized opportunities is supplied by the frozen realized-event log, prepopulated in the form and locked: it is common to both raters and is not a rater judgment. The rater enters only the two rater-determined quantities, namely how many of those opportunities were observable for the designated participant and on how many the criterion was met. Writing *p* for the proportion of observable opportunities on which the criterion was met, the platform derives the ordinal band *b* from the prespecified boundaries:

$$b=\{\begin{array}{ll} 5, & p\geq 0.90\\ 4, & 0.70\leq p<0.90\\ 3, & 0.40\leq p<0.70\\ 2, & 0.10\leq p<0.40\\ 1, & p<0.10 \end{array}\;\;\;\;$$ (1)

The band is therefore computed rather than judged, and every quantity remains available for audit. Because the band coarsens the entered counts, agreement is prespecified on the two rater-determined counts as well as on the band, but not on the realized-opportunity count, which is common by construction. The band boundaries are design conventions requiring calibration, and are not evidence-based cutoffs. Because the five bands of Equation 1 map proportions onto a five-point scale, fewer than four observable opportunities leave some bands unattainable by construction. Such items are scored and reported with their denominators, and the distribution of denominators is reported among the measurement-process indicators.

The caregiver channel cannot enumerate opportunities across a 48-h window. For items 1–6, caregivers use everyday-language frequency anchors when at least one relevant situation occurred and the behavior was directly observed. The category approximates the clinician-channel bands but is not calculated from a denominator, and no denominator is recorded. The channels share the ordinal range, scoring direction and non-observability pathways but differ in the response process by which the category is obtained. That difference concerns how the rating is produced and not merely how finely it is resolved, and it bounds the interpretation of cross-context correspondence.

The reverse-scored seventh item uses episode count because a disruptive event is not opportunity-bounded: the clinician channel counts episodes within the 20-min segment, the caregiver channel across the 48-h interval. Item 7 is the only item whose denominator is time and not discrete opportunities, and domain-level cross-context correspondence is prespecified to be reported with and without it. Its non-observability routing differs accordingly: zero qualifying episodes within an adequately observed window is entered as a raw episode-band of 1 (no episodes), which reverse-scores to 5. It is not coded S-NA, which does not apply to item 7 because there is no opportunity to be absent. NO is used only when the window was insufficiently observable to determine whether episodes occurred, and TECH remains reserved for technical failure. Episode counts are entered and the raw band derived by the platform, as for items 1–6, and the item score is obtained as 6 minus that raw band. All seven Emotional–Motivational Regulation items refer to observable behavior and require no attribution of an internal state. The grid is an anchoring specification, not an empirical result. Band boundaries will be calibrated against the training material before the first live session, and each item is classified *a priori* as score-determined or endogenous from the task structure. Explicit eliciting conditions and coding criteria do not establish reliability or validity. The session instrument shares methodological features with Direct Behavior Rating (34) but differs by using replayable recorded evidence, event-defined scoring opportunities, count-derived response categories for items 1–6 and a separate paired observer-report channel.

The grid applies only to externally observable behavior and does not assess participant experience. Its rules constrain the rater’s classification of observed behavior. The observable basis of the domain labels varies across domains. In Domains A, B, D and E the referents derive from task requirements. Entries, written durations, rests during another section’s passage, notated dynamics and tempo changes are properties of the part and the ensemble, and would be identified in the same terms by any musician. In observational measurement these are low-inference categories anchored to physically defined events (17). In Domain C the label is carried by the domain heading alone. Retention of Domain C is therefore a prespecified decision, resolved in phase 1 against the four revision conditions.

#### 3.1.10 Non-observability, missingness and aggregation rules

The instrument distinguishes three mutually exclusive non-numeric response options at item level. NO denotes an opportunity that arose but a behavior that could not be observed within the reporting window. S-NA denotes no opportunity within the window. TECH denotes a platform-level interruption or device error. These options are exhaustive only among the instrument’s coded non-numeric responses. Uncoded missing items and whole-form non-submission are recorded separately as missingness and quality-control outcomes.

A behavior for which an opportunity occurred, the participant was observable and the criterion was not met receives a numeric rating of 1, not NO. NO is excluded from aggregation whereas a rating of 1 contributes to the domain mean, so misclassifying non-occurrence as NO would remove floor observations and bias the mean upward. The applicable item-level rules are specified in Supplementary material S5, and Figure 2 summarizes the codes and their scoring consequences.

Domain means are unweighted within-domain means of the available item scores, fixed before data collection so that scoring is deterministic, and the rule is an arithmetic convention for summarizing the available item scores. A domain indicator is computed only when at least 5 of its 7 items have valid ratings. Otherwise, the domain score is suppressed and logged. This 5-of-7 domain-score availability rule is a prespecified study convention, not an externally validated cutoff, and the full codebook and aggregation rules are given in Supplementary material S7. Because the rule permits a domain mean to be computed over different item subsets on different occasions, the identity of the contributing items is recorded with each domain summary and reported with it.

#### 3.1.11 Data quality and governance

Automated quality-control functions will be configured to monitor reporting-window compliance, incomplete entries and non-differentiated response patterns (Table 9). Their correct operation will be verified through user-acceptance testing. The indicators follow the completeness, consistency and plausibility dimensions used in data-quality frameworks for observational health research collections (35). Pattern flags are descriptive review triggers, and do not independently invalidate a record or support an inference about insufficient-effort responding.

**Table 9:** Quality-control flag logic and data clarification request workflow.

QC flag	Trigger logic	System variable (Supplementary material S6)	Response strategy (Supplementary material S7)
QC_WINDOW_NONCOMPLIANT	Submission >24 h (ClinRO-T) or >48 h (ObsRO-P)	SUBMISSION_LATENCY_HOURS	Retained in the all-submitted set; excluded from the primary window-compliant analytic set; retained for sensitivity summaries
QC_PATTERN_ANOMALY	Invariant/straight-line patterns	PATTERN_TYPE	DCR initiated
QC_TECH_FLAG	TECH code selected	ITEM_TECH_FLAG	Item excluded; device troubleshooting
QC_DOMAIN_UNAVAILABLE	Fewer than 5 of 7 valid items in domain	DOMAIN_VALID_ITEMS_N	Domain mean suppressed; logged as missing
QC_INCOMPLETE	Missing responses without NO/S-NA	ITEM_COMPLETE_FLAG	DCR issued
QC_REVIEW_TRIGGER	20% of submitted ObsRO-P forms selected	REVIEW_STATUS	Prespecified response-process QC review

The prespecified quality-control and audit-trail procedures are designed to support procedural adherence and data integrity through contemporaneous recording, preservation of original submissions, traceability and auditability (39). ClinRO-T: clinician-reported outcome, therapy-session channel; ObsRO-P: observer-reported outcome, caregiver channel; DCR: Data Clarification Request; TECH: technical interruption; NO: Not Observed; S-NA: Structural Not Applicable.

A prespecified 20% subset of ObsRO-P submissions will undergo response-process quality-control review by an OPBG Clinical Research Coordinator, who will be appointed before study initiation. This review is an operational quality-control procedure and is not part of the inter-rater agreement analysis. Flagged entries will initiate a structured Data Clarification Request workflow. The anomaly, the clarification requested and the procedural resolution will be recorded. Original entries will never be overwritten, and clarifications will be appended to the audit trail.

#### 3.1.12 Data architecture and general data protection regulation governance

Privacy by design will be implemented through pseudonymization with key separation. The analytical dataset will contain pseudonymous participant and session identifiers but no direct identifiers. The re-identification file will be stored separately under the control of OPBG, the planned Data Controller. Comparable approaches to pseudonymization and to the management of pseudonymized research data have been described previously (36–38). Access will be role-based, and Supplementary material S11 specifies the planned governance, retention and audit-trail procedures. These procedures are informed by risk-based data-integrity principles described for research conducted outside formal regulatory quality systems (39). They do not establish formal regulatory compliance or constitute validation of the electronic system.

#### 3.1.13 Ethical framework

Because this manuscript reports only methodological development and involves neither enrollment nor identifiable human research data, ethics approval was not required for the pre-implementation work. The planned study is intended to be sponsored by OPBG, acting as institutional data controller, and conducted at Euterpe APS under a formal inter-institutional agreement. No study-specific recording or outcome collection will begin before institutional feasibility assessment, a favorable opinion from the competent Territorial Ethics Committee, completion of the applicable data-protection requirements and documented informed-consent and assent procedures. Recruitment of adults will not begin until OPBG has authorized the applicable adult transition pathway or a written agreement with ASL Roma 4 has been executed. That agreement defines diagnostic verification, referral, safety-escalation and data-governance responsibilities. Study-specific authorization will cover research video recording, secure institutional storage and independent secondary rating under the masking conditions of Supplementary material S3.

Consent will be obtained from every identifiable person with decision-making capacity. Parental or legally authorized representative consent and developmentally appropriate assent will apply where required. Dissent expressed during a session, verbal or behavioral, will suspend recording for the participant concerned and the corresponding segment will be excluded before research access. Identifiable professionals, musicians and volunteers will provide separate authorization. Persons without it will remain outside the recording field or be masked. Before the first study recording, Euterpe APS will designate and train an Operational Site Representative independent of outcome rating. That person will verify documented authorization for persons present, manage recording-field logistics and report site-level deviations to the OPBG Clinical Research Coordinator, without access to rating outputs. Further safeguards include pseudonymization with key separation and role-segregated access control (Supplementary material S11).

#### 3.1.14 Worked example

A partial worked example on a participant’s assigned part is given in Figure 1. Five score locations show the notated event, the conductor cue at that location, the item code activated and the observable criterion applied, spanning the Cognitive–Executive, Motor–Sensorimotor and Synchronization domains. The example is restricted to opportunities generated by the score. Opportunities arising from an unplanned event (an audible error, A5; a detected loss of synchrony, E4; the episode counts of the reverse-scored seventh items) have no presession antecedent and are identified from the realized-event log alone. The two classes are distinguished *a priori* in the observation grid, which fixes the observable referent for each of the 35 items, the event that creates its scoring opportunity in each channel, and the cases that void it (Supplementary material S5).

### 3.2 Instrument development and provenance

The development of the instruments, the rationale for the design choices, the options considered and set aside, and the content-development steps still outstanding are documented separately from the protocol above, so that the protocol can be read on its own.

The system was specified as a dual-informant architecture for observation in the therapy session and in daily life, using established ClinRO/ObsRO definitions and protocol-level traceability for electronic capture (7–9). Item banks and behavioral anchors are given in full in Supplementary material S1 (ClinRO-T) and Supplementary material S2 (ObsRO-P).

#### 3.2.1 Domain and item development

Development proceeded through sequential stages. Exploratory item pools published with the antecedent Perspective (13), and developed with clinical input in a prior academic collaboration external to the present author group, provided the source material for the present instruments. Before enrollment, these pools underwent multidisciplinary clinical and methodological re-specification by clinical psychotherapists, a child neuropsychiatrist, a neurologist and the conductor–music therapist. This process produced two 35-item candidate instruments, each organized into five domains of seven items. Domain selection and subsequent item refinement followed direct observability in the orchestral and daily-life contexts, in preference to an *a priori* taxonomy of impairment. Item wording was restricted to referents that a rater can identify without inferring an internal state. Item count and domain structure remained unchanged during item-level refinement, and the complete development audit is documented in Supplementary material S12.

No formal consensus procedure, cognitive interview or wording pilot has yet been conducted. Following a favorable opinion or formal determination from the competent ethics committee and completion of the applicable data-protection requirements, the current candidate item sets will undergo a prespecified modified Delphi involving independent multidisciplinary experts, intended ClinRO-T raters, ObsRO-P caregiver observers and, where appropriate, persons with NDD. The procedure will use iterative rounds with anonymous ratings, controlled feedback and *a priori* criteria for item retention, revision, removal and addition. The Delphi-derived version will subsequently undergo cognitive interviewing with the intended raters and caregiver observers, with all item-level decisions documented under version control before the phase 1 forms are frozen (10, 40–42).

The version intended for administration is Italian, whereas the English wording presented in this manuscript is for publication only. No linguistic-equivalence claim is made for the English version unless formally established. Comprehensibility and cognitive testing of the Italian version are part of these pre-freeze procedures. Good-practice recommendations for content development include cognitive interviewing with the intended observer population (40), an approach used in content-validity development of autism observer-report scales (43). Because these procedures remain pending, content validity is not claimed for the present candidate instruments (44).

Developing a new instrument, instead of modifying an existing COA, requires an explicit fit-for-purpose rationale (45). Existing COAs considered by the development group did not directly operationalize temporal alignment with an ensemble or context-bound coordinated action during orchestral performance. No systematic review of existing NDD instruments was conducted, and suitable measures may exist elsewhere in the literature. Validated instruments are retained for the phase 2 randomized study to assess established constructs alongside the context-specific indicators developed here.

#### 3.2.2 Functional domain selection and neurofunctional rationale

The five domains were selected for observable functional variation in adolescents and adults with NDD, conditions that frequently persist into adulthood and affect several functional systems (20). Two criteria operated in sequence. Observability in the ensemble setting fixed the admissible content: a domain was retained only if its referents can be identified by a rater from recorded evidence without inferring an internal state. This excludes constructs of clinical interest that the setting does not expose. Neurofunctional considerations then governed the grouping and labeling of the retained referents. The domain labels are consequently organizing conventions applied to observable behavior. Evaluation of the adequacy of the item–domain structure is reserved for a subsequent purpose-designed validation study.

Two cross-domain associations identified in the scoring structure are retained in Figure 2. The literature cited in support of each provides context only, and does not define or validate the scoring structure.

The association between the Cognitive–Executive and Emotional–Motivational Regulation domains (A↔C) follows from the grid, in which single occasions generate opportunities in both. An audible performance error opens an opportunity for A5, which scores resumption latency, and for C2, which scores whether overt distress ends within 60 s. A correction from the conductor opens an opportunity for A3 and for C3. The domains are rated against different referents but are not statistically independent, and the dashed link in Figure 2 records that dependence, not a hypothesized process. The association is contextually consistent with accounts of cognition–emotion interaction (4) and with transdiagnostic framing of overlapping neurodevelopmental profiles (5). Systematic-review and meta-analytic evidence documents executive function and emotional dysregulation in autism and Attention-Deficit/Hyperactivity Disorder (ADHD), and executive-function differences across neurodevelopmental conditions (46, 47).

The association between the Socio–Communicative and Synchronization domains (B↔E) is constitutive of the setting, not inferred. Ensemble synchronization is defined only relative to performing partners. E4 and E5 have no referent in the absence of a section that is itself in time, and the grid voids the opportunity when the section loses synchrony (Supplementary material S5). Both kinds of opportunity are generated by the same ensemble structure. Research on interpersonal entrainment and joint music making identifies coordination and temporal alignment as central to real-time ensemble performance (48, 49); related work documents interbrain synchrony during duet performance (50) and rhythm and timing vulnerabilities across neurodevelopmental conditions (51). The scoring architecture includes no neural measure.

The Motor–Sensorimotor domain (D) is recorded alongside the other four with no hierarchical status. Its items score execution and coordination against referents fixed by the instrumental part and by the everyday task. Motor difficulties are documented across autism, ADHD and developmental coordination disorder (52). The reported differences include gross-motor performance and its association with social skills in autism (53), sensorimotor and manual dexterity beyond childhood (54), and sensory processing and broader functional involvement in developmental coordination disorder (55, 56). This literature supports recording motor behavior as contextual information but does not assign causal or hierarchical status to Domain D. Two item-level overlaps are documented in the grid: D3 and B2 may be scored on the same gestural cue, with B2 scoring social responsiveness and D3 motor execution. Ordering errors in D4 are separated from timing errors, which are scored under Domain E.

Items 1–6 and item 7 use different metrics, and three Domain C items index the same behavior class, overt distress and agitation, under different eliciting conditions and at different durations. No structural analysis is performed or planned at this observation density.

#### 3.2.3 Alignment with methodological standards

This phase concerns instrument development, and the two instruments are positioned within the COA framework as a ClinRO rating and an ObsRO measure (7–9), addressing early framework elements: context of use, concept of interest and content development. Within the U. S. Food and Drug Administration framework for patient-focused outcome measurement (10), the instruments occupy the transition from a measure under development to the beginning of COA development. At that point, documenting content validity through qualitative and mixed-methods work is the prescribed next step, whereas cross-sectional and longitudinal measurement properties belong to later stages. Item refinement completed before enrollment forms part of instrument development and does not constitute independent evidence of content validity, which remains subject to the prespecified pre-freeze procedures. Content-validity concepts from the COnsensus-based Standards for the selection of health Measurement INstruments (COSMIN)—relevance, comprehensiveness, comprehensibility and cognitive testing with target observers—were used only to identify outstanding development evidence, that guidance having been developed for patient-reported outcome measures (44). The regulatory guidance on fit-for-purpose COA sets out the corresponding expectations for clinician- and observer-reported measures: reporter definition, observable referents, response options, rater training and standardized administration (10). The sampling plan, rater training and agreement procedures follow systematic-observation conventions (16, 17). These sources inform instrument development and systematic observation but are not used as conformity or appraisal instruments, and the present work is not a regulated submission. The program in which observation occurs is the measurement context, not an intervention under evaluation.

#### 3.2.4 Documentation and prespecification

The protocol traceability matrix (Supplementary material S12) records where each methodological element is specified and what remains planned. Fixing the scoring rules, the non-observability routes, the 5-of-7 domain-score availability rule and the quality-control triggers before data collection is intended to limit analytical flexibility in phase 1.

## 4 Anticipated results

The outputs prespecified for phase 1 fall in three areas, namely implementation feasibility, preliminary measurement performance and evidence relevant to the design of the subsequent validation study. Each research question is linked in Table 10 to the feasibility and monitoring indicators through which it will be addressed. This section defines the planned reporting and does not present observed results, predicted findings or empirically validated thresholds.

**Table 10:** Pre-implementation feasibility and monitoring indicators.

Indicator	Reporting specification	Rationale
Completion rate	Estimated prospectively	Reported by channel and week with numerator and denominator; no imported benchmark
Handling of missing data	NO/S-NA/TECH classified; no imputation	Preserves distinct reasons for missingness; category-specific and window-compliance sensitivity summaries are planned prospectively
Caregiver digital literacy	Structured onboarding + supervised trial	Provides standardized exposure to the interface before independent reporting; effects on completion time, missingness and response variability are empirical
Response-process monitoring	QC flags + prespecified response-process QC review	Aligns with recommendations for documenting response processes during development
Content relevance (RQ2)	After phase 1: cognitive debriefing of raters and caregivers; per-item clarification-request frequency	Addresses whether the five domains are interpretable and usable under naturalistic reporting conditions
Informant independence (RQ3)	Documented breaches of access segregation; informant replacement rate; ObsRO-P submission timing relative to ClinRO-T	Addresses whether the procedures keep the two channels independent
Data governance (RQ4)	Audit-trail completeness; user-acceptance testing outcome; role-based access verification	Addresses whether the system supports secure, traceable and auditable data handling
External anchoring (RQ5)	Vineland-3 score distribution and exploratory correspondence with domain summaries, on Psychologist B’s locked records	Addresses whether the Vineland-3 is suitable as an external interpretive reference; A’s records reported separately for common-rater variance
Score composition	Item set contributing to each domain summary; proportion of summaries suppressed under the 5-of-7 domain-score availability rule	Domain means computed over different item subsets are not directly comparable and are reported with their composition
Agreement resolution	Agreement on entered counts, on the derived ordinal band, and by denominator class	The derived band coarsens the entered counts; opportunity denominators vary with the instrumental part
Observation-window adequacy	Estimated prospectively	Opportunity yield and NO/S-NA summarized by phase and domain, together with inter-rater agreement, coding burden and missingness; no imported threshold

These are prespecified reporting domains for the subsequent study, not results or validated thresholds. Completion, latency and form-entry duration will be described prospectively by channel without a fixed validity cutoff or an imported completion-rate benchmark. COA: Clinical Outcome Assessment; ObsRO-P: observer-reported outcome, caregiver channel; QC: Quality-Control.

### 4.1 Feasibility and implementation outputs

Active coding time and form-entry duration will be estimated prospectively, and are not fixed *a priori*. Platform timestamps will record form opening and submission, and additional replay or coding time will be logged. These quantities are feasibility outcomes and do not determine record validity in isolation. The full 20-min segment remains the observational evidence for each designated target, so elapsed coding time is not inferred from segment duration and is not used to determine the number of targets. Each scored session generates one ClinRO-T record per designated target, bounding reporting load independently of group size.

No external completion-rate benchmark is prespecified because the applicability of estimates derived from other reporting paradigms cannot be assumed for this interval-based, dual-informant design. Completion, submission latency and missingness will be estimated prospectively by channel, participant and study week and reported with their denominators. User-acceptance testing will verify platform constraints, timing capture, routing logic and non-observability functions before implementation (23). These outputs will characterize the observed reporting burden and operation of the planned workflow without introducing an unprespecified pass–fail threshold.

### 4.2 Preliminary measurement-performance outputs

Measurement-performance outputs will be characterized within the planned sample. That sample, 20 participant clusters and 35 items, supports feasibility and inter-rater agreement, and structural evaluation is deferred to a subsequent purpose-designed validation study. The analyses are limited to inter-rater agreement, item-level distributional behavior, non-observability and score-availability indicators, and exploratory cross-context comparison (Supplementary material S10), using methods appropriate to ordinal observer ratings and clustered repeated observations (57, 58).

Agreement is prespecified at three levels, on the two rater-determined counts for items 1–6, on the ordinal band derived from them, and on domain means. The five observations contributed by each participant will be treated as clustered and not as independent, and, since opportunity denominators vary with the instrumental part, agreement will also be reported by denominator class.

Both raters will work from the same score map and realized-event log. The realized-opportunity count is therefore not a rated quantity, and an error in either source would propagate to both ratings. Agreement between A and B will estimate concordance in rating given a common identification of the scoring occasion, and not concordance in identifying the occasion itself. Standardization on that layer may yield higher agreement than an unstandardized design, by a margin the planned data cannot estimate.

Signals for domain review before the validation study are prespecified as four observable conditions, each reported irrespective of its direction. Near-invariant item distributions may indicate limited discrimination within the observed cohort and would prompt item review in place of automatic removal. A concentration of NO or S-NA frequencies in one domain may indicate that the setting does not expose that domain at the assumed rate, which would bear on domain admissibility and not by itself on domain labeling. Inter-rater agreement markedly lower in one domain than in the others would prompt a diagnostic review before any structural interpretation. Potential sources include referent ambiguity, insufficient training, the perceptual demands of the items, an inadequate score map or realized-event log, and audio-video quality. Only referent ambiguity persisting after that review would count as evidence relevant to structural revision, and Domain C is the prespecified candidate for this appraisal. Inter-item associations inconsistent with the assumed grouping would be examined descriptively. None of the four conditions constitutes a structural test, and each will be reported as evidence relevant to appraisal of the current structure.

The impact of the 5-of-7 domain-score availability rule will be examined descriptively, by reporting the proportion of domain summaries suppressed under it and the item composition of those retained. Where the conditions indicate that Domain C may not cohere, the candidate reconfiguration reassigns its regulatory items to the Cognitive–Executive domain and retains the remainder as a separate disruption index, and no other regrouping is prespecified. Domain C will also be summarized in a reduced representation retaining one member of the C2/C6/C7 set, since these three items index the same behavior class. The retained item will be selected during the pre-initiation content review on grounds of relevance, direct observability and non-redundancy, documented in the item-level development audit and frozen before enrollment, so that comparison of the complete and reduced representations involves no data-dependent item selection.

The paired structure allows exploratory examination of clinician–caregiver correspondence, on the understanding that therapeutic and naturalistic settings may capture distinct behavioral manifestations. Correspondence will be described separately for each locked ClinRO-T rating, rater A against the matched ObsRO-P record and rater B against the same record, as parallel analyses that are neither pooled nor averaged. Differences between the two estimates would be interpreted in relation to rater dependence within the clinician channel and not attributed to the daily-life channel. Domain-level correspondence will be reported with and without item 7, whose denominator is time.

Domain indicators will be described at cohort level across the five observations per participant, and by age band as a descriptive stratum. Individual trajectories are not estimable at that observation density, and no trajectory or state-space model is planned. Opportunity yield, non-observability and domain summaries will be reported by segment position, which the interleaved schedule renders approximately orthogonal to study week and cumulative exposure. These analyses will remain descriptive because the design includes no comparison condition. Missingness will be summarized by prespecified category and observation occasion.

### 4.3 Interpretive scope and implications for validation

The scoring and reporting rules are defined, whereas the measurement properties of the instruments are not yet empirically established. Items are scored on a five-point ordinal scale, which constrains the granularity of observable score differences. Floor or ceiling concentrations may occur for referents nearly uniformly present or absent in the cohort. The non-observability pathways delimit behaviors for which no valid numeric score is obtained.

Within-person score variation across the five scheduled observations will be described in phase 1. Estimation of responsiveness, measurement error, standard error of measurement and minimal detectable change is reserved for a subsequent validation design capable of distinguishing measurement error from change over time.

The domain mean combines a proportion for items 1–6 with an episode count for reverse-scored item 7 on the same ordinal scale and applies no differential weighting. Aggregating them on a single scale assumes commensurability for scoring purposes, and empirical support for that assumption remains to be examined. Among observations with complete numeric data for all seven items, each domain will additionally be summarized using items 1–6 only, and the paired difference from the corresponding seven-item domain mean will be reported descriptively. This analysis will evaluate the influence of the episode-count item without modifying the prespecified primary domain score.

Video permits enumeration of opportunities and verification of second-level latencies, whereas a caregiver reporting over 48 h cannot enumerate every opportunity or apply fine temporal thresholds. Item-level correspondence will therefore be interpreted within the resolution of the coarser channel.

A single episode may satisfy Domain C items C2, C6 and C7 and so contribute three times to the domain mean, with the effect of weighting overt distress and agitation disproportionately in the domain summary. The prespecified reduced representation will allow that influence to be examined without replacing the complete domain score.

Domain E does not represent an identical behavioral domain across the two channels. Ensemble synchronization is defined relative to performing partners and a written pulse, conditions that daily life does not reproduce, whereas ObsRO-P indexes pacing and coordination with a single partner. Correspondence is therefore more context-dependent here than for Domains A, B and D. Low correspondence would be interpreted first in relation to that contextual difference, and not by itself as evidence against either channel.

The five summaries are retained separately, and no global cross-domain score is prespecified, because their commensurability as indicators of a single superordinate construct has not been established. Completeness is accordingly represented by domain-specific availability and by the number of available domains, ranging from 0 to 5, and not by a mean across domains.

The 5-of-7 domain-score availability rule permits a domain mean to be computed from different item subsets on different occasions. Such means are not directly comparable unless their composition is considered, and the contributing item set will be reported with each summary.

The five domains map only partially onto recognized taxonomies of human functioning such as the International Classification of Functioning, Disability and Health (59). The set is delimited by what the ensemble setting exposes, so that functions of clinical relevance outside that exposure remain outside the instruments’ coverage and the domains do not constitute a comprehensive functional profile.

The instrumental part determines the density and difficulty of scoring opportunities, since percussion and first-violin parts do not necessarily present equal numbers of entries, dynamic changes or tempo changes within a 20-min segment. Denominators therefore vary across participants, and a low denominator is associated with greater sampling uncertainty within the same ordinal band. The rotation is stratified by section, the assigned part is recorded, and agreement is reported by denominator class, procedures that support interpretation of between-part heterogeneity without removing it.

The repertoire remains fixed for 20 weeks, and each participant performs the same personalized part at every session, which stabilizes the opportunity structure across the five observations but allows familiarization to accrue. An across-session difference would therefore remain compatible with both behavioral change and familiarization, which the planned design cannot separate, and generalization to other repertoire likewise remains to be examined.

These outputs will inform the design of the subsequent validation study, without extending interpretation beyond the observational evidence that phase 1 will actually produce.

## 5 Discussion

### 5.1 Methodological contribution

This manuscript prespecifies a dual-informant COA system for naturalistic monitoring of behavioral domains in adolescents and adults with neurodevelopmental disorders. Its methodological contribution is the integration, before data collection, of two context-adapted instruments, an item-level observation grid, three non-observability routes, fixed reporting windows, auditable electronic capture and separated rater roles. Psychologist A observes the session, whereas Psychologist B independently codes the same recorded evidence while masked to prior ratings and to clinical and caregiver information.

The prespecified five-domain model organizes scoring and is not treated as an empirical result. Referents were selected for direct observability in the ensemble and daily-life settings before they were grouped into domains.

The two-layer annotation procedure separates identification of scoring occasions from behavioral rating. This allows both raters to assess the same event information without requiring independent musical annotation, but any error in the shared annotation layers will affect both ratings.

### 5.2 Implications and future directions

Prespecified calibration, quality-control procedures and governance are intended to make phase 1 outputs interpretable, and do not establish measurement properties. The development pathway comprises four stages, namely pre-initiation content development, the phase 1 observational study, a subsequent purpose-designed validation study and a separate randomized study of the program. Phase 1 will examine whether the five domains are observable at the assumed rate and whether independent raters apply their referents consistently. Structural validity, responsiveness, measurement error and evaluation of alternative item groupings belong to the subsequent validation study, which must be designed for those purposes. The separate randomized study will use validated instruments of motor proficiency, executive function, adaptive behavior and quality of life alongside the context-specific indicators. Its comparison condition, outcome hierarchy and analysis plan are specified separately, and it is not an instrument-validation design.

### 5.3 Other limitations

The instruments remain psychometrically untested, and no measurement property is established for the current candidate version. Mixed non-probability self-referral and clinical referral may overrepresent motivated individuals, so that population-representative estimates are not claimed. The system is moreover specified on a single ensemble, at a single site, with a single conductor–music therapist, and its transportability to other ensembles and directors is untested. Repeated reporting and variable coding requirements may impose burden on raters and caregivers, and coding and form-entry durations are therefore reported as feasibility outcomes. Technological accessibility may also influence participation, despite the structured onboarding provided to caregivers. The parallel instruments assume a partial conceptual alignment across contexts whose extent remains to be determined.

The occasion-identification layer is produced once by a single annotator and is not independently replicated in phase 1, so inter-rater agreement is conditional on that layer and does not estimate its accuracy. The error rate of the layer is undetermined, and double annotation of a subset, a candidate addition for the validation study, would bound it. The order in which segments are coded is neither randomized nor counterbalanced. Carryover across the 20 sessions and within-participant familiarity therefore cannot be separated from genuine agreement, and scoring drift will be examined descriptively but not controlled by design.

Every COA rating rests on judgment that no anchoring scheme removes entirely. Item-level anchoring, role segregation and independent double-coding constrain scoring variability, but expectancy effects in Psychologist A and contextual inference by Psychologist B persist.

## 6 Conclusion

This manuscript prespecifies the operational and governance architecture of two context-specific candidate clinical outcome assessments, ClinRO-T for recorded orchestral-session behavior and ObsRO-P for directly observed daily-life behavior. The channels remain separate and are not assumed to be metrically equivalent. A modified Delphi process and cognitive interviews will precede form freeze, and phase 1 will evaluate feasibility, prespecified measurement-process and governance indicators, and the suitability of Vineland-3 as an external interpretive reference. No participants have been enrolled, no psychometric data are reported, and neither measurement properties nor the structural adequacy of the five-domain model are claimed as established.

## Data Availability

The original contributions presented in the study are included in the article and Supplementary materials S1–S12; further inquiries can be directed to the corresponding author.
