# Supplementary material for: Dual-informant clinical outcome assessment in the inclusive orchestra: measurement architecture for neurodevelopmental disorders prior to empirical evaluation
Source: Front Neurol. 2026 Sep 29;17:1834441. doi: 10.3389/fneur.2026.1834441 (PMC13660360; doi:10.3389/fneur.2026.1834441)
Supplement: Supplementary file 1 [file Data_Sheet_1.PDF]

Supplementary Material S1

ClinRO-T instrument

1 Scope and objective

ClinRO-T is completed independently by two psychologists from the same replayable 20-minute OMT segment within 24 hours after the scored session. It characterizes directly observable behavior in the therapy-session context. The planned phase 1 cohort will include participants aged 14–32 years at consent; no participant is enrolled. Recruitment, eligibility, diagnostic verification, safety, attendance, withdrawal and focal-participant sampling procedures are specified in sections 3.1.2, 3.1.3 and 3.1.4 of the main manuscript and apply to all participants assessed using ClinRO-T. The wording reported below constitutes the current candidate form. S5 specifies the corresponding scoring opportunities, observable criteria and boundary rules. The administration version will be frozen only after the planned modified Delphi and cognitive-interviewing procedures described in section 3.2.1 of the main manuscript and S12, and before participant outcome collection begins.

2 Methodological principles

- Direct observability: ratings are restricted to behavior visible or audible in the recorded segment; no item requires inference about an internal state.
- Informant and rater independence: each psychologist completes ClinRO-T independently, without access to the other psychologist's entries or to the matched caregiver-reported ObsRO-P record before the ClinRO-T records are locked.
- Interpretive scope: phase 1 analyses are restricted to implementation feasibility, inter-rater agreement, item- and domain-level distributions, non-observability, score availability and exploratory cross-context correspondence. The scope of phase 1 and the elements deferred to a subsequent purpose-designed validation study are specified in S10. The five observations per participant do not support estimation of individual short-interval trajectories.

3 Observational window and response scale

- Observational window and completion deadline: ratings refer exclusively to one continuous 20-minute segment. Across the 20 scored sessions, segment position follows the prespecified schedule of seven opening, six middle and seven closing segments. Each rating must be completed within 24 hours after the session; this is an operational coding deadline intended to limit backlog and cross-session contamination, not a criterion of measurement validity. The 20-minute duration is a prespecified sampling parameter rather than a validated cutoff. Its adequacy will be evaluated prospectively through opportunity yield, phase- and domain-specific NO/S-NA frequencies, inter-rater agreement, coding burden and missingness (29-32).
- Ordinal response scale (1–5): for items 1–6, the realized-opportunity count is supplied by the frozen realized-event log, prepopulated in the form and locked. The rater records the number of opportunities that were observable for the designated

participant and the number on which the behavioral criterion was met. The platform derives the ordinal category from the proportion of observable opportunities on which the criterion was met: 5 = ≥90%; 4 = 70–89%; 3 = 40–69%; 2 = 10–39%; and 1 = <10%. These categories are computed from the recorded counts rather than selected by the rater from verbal frequency labels. Item 7 is scored from the number of qualifying episodes within the 20-minute segment (S5). The band boundaries are prespecified design conventions to be examined against the calibration material before the first scored session (S3, S5); they are not evidence-based cutoffs.

- Non-observability pathways: for items 1–6, S-NA is used when no scoring opportunity arose within the segment. NO is used when one or more opportunities arose, but none was observable. When only a subset of opportunities was observable, the item is scored using that subset and is not coded NO. A participant being outside the recording field is one possible cause of non-observability, not its definition. A numeric rating of 1 is assigned when an opportunity arose, the participant was observable and the criterion was not met. For item 7, S-NA does not apply because scoring is based on episode count rather than discrete opportunities. Zero qualifying episodes during an adequately observed segment produces a raw episode band of 1, which reverse-scores to 5; NO is used only when the segment was insufficiently observable to determine whether episodes occurred. TECH denotes a technical failure preventing observation or data entry. TECH records are retained, excluded from aggregation and flagged for quality-control review. NO and S-NA are also excluded from aggregation. Full item-level rules are provided in S5.

4 Functional domains and paired context-adapted items (items 1–6 positive; item 7 negative / reverse-scored)

DOMAIN A — Cognitive–Executive

A1. Initiates task on signal;
A2. Maintains attention ≥5 min;
A3. Follows changes in instructions;
A4. Completes musical exercise;
A5. Resumes activity after error within 5 s;
A6. Switches sections without hesitation;
A7. Non-functional interruption of the trial (reverse-scored).

DOMAIN B — Socio–Communicative

B1. Orients body toward director;
B2. Responds to gesture within 3 s;
B3. Interacts with section peers;
B4. Waits for musical turn;
B5. Assists peer upon request;
B6. Maintains gaze toward director ≥3 s;
B7. Withdraws from the group (reverse-scored).

DOMAIN C — Emotional–Motivational Regulation

C1. Remains at own station in playing posture for the assigned passage;
C2. Overt distress after an error or a correction ends within 60 s;
C3. Attempts the corrected action without verbal or gestural refusal;
C4. Requests assistance without raised voice, throwing, or leaving the seat;
C5. During waiting intervals ≥30 s, remains seated and oriented to the ensemble;
C6. Returns to task-oriented posture within 60 s after agitation signs;
C7. Sustained overt distress lasting >5 min (reverse-scored).

DOMAIN D — Motor–Sensorimotor

D1. Maintains instrumental posture;
D2. Coordinates hands/limbs;
D3. Moves limb on signal;
D4. Executes motor sequence;
D5. Regulates pressure/touch;
D6. Moves safely during transitions;
D7. Repetitive non-functional movement (reverse-scored).

DOMAIN E — Synchronization

E1. Enters on the beat;
E2. Maintains rhythm ≥10 s;
E3. Changes dynamics on signal;
E4. Re-synchronizes within 5 s;
E5. Synchronizes with section;
E6. Changes tempo on request;
E7. Loss of rhythm (reverse-scored).

5 Scoring and integrity rules

- Reverse-scoring: item 7 in each domain (A7–E7) is automatically reverse-scored by the platform after derivation of the corresponding episode-count band.
- Domain-score availability rule: a domain mean is computed only when at least 5 of its 7 items have valid numeric ratings. NO, S-NA and TECH do not contribute to the domain mean; TECH is additionally flagged for quality-control follow-up.
- Data governance: server-side timestamping, post-submission locking, and audit-trail logging are prespecified to support contemporaneity and traceability in electronic COA capture; their correct implementation will be verified through user acceptance testing (23).

Supplementary Material S2

ObsRO-P instrument and onboarding protocol

6 Scope and objective

This document defines the caregiver-reported ObsRO-P instrument. It covers behavior directly observed during the 48-hour interval after each scored OMT session and is submitted by the end of that interval. Before enrollment, each participant nominates one primary daily-life informant (parent, caregiver, or regular support person); an adult participant authorizes that person. The informant has no role in OMT delivery, clinical supervision, outcome rating, or program volunteering. The same person independently completes all five target-week forms without access to ClinRO-T. Only behavior directly observed at home, school, or in the community is rated; second-hand accounts are excluded.

The wording reported below constitutes the current candidate form. S5 specifies the corresponding scoring situations, observable criteria and boundary rules. The administration version will be frozen only after the planned modified Delphi and cognitive-interviewing procedures described in section 3.2.1 of the main manuscript and S12, and before participant outcome collection begins.

7 Methodological principles

- Direct daily-life observation: ratings are based only on behavior directly observed by the designated informant in everyday settings.
- Burden assessment: form-entry duration is timestamped and summarized prospectively; no fixed completion-time threshold is imposed.
- Informant independence: the designated informant completes each ObsRO-P form without access to ClinRO-T entries.

8 Observational window and response scale

- Observation interval: ratings cover behavior directly observed during the 48 hours following the scored OMT session. The form is submitted by the end of that interval.
- Response scale: ObsRO-P uses the same 1–5 ordinal range, scoring direction and NO/S-NA/TECH pathways as ClinRO-T, but the response process differs between channels. For items 1–6, the informant selects a frequency category based only on relevant situations directly observed during the 48-hour interval: 5 = every time or almost every time; 4 = most times; 3 = about half the times; 2 = a few times; and 1 = never or almost never. No opportunity denominator is recorded and the category is not calculated from a proportion. The channels are therefore not assumed to be metrically equivalent. A numeric rating is entered only when at least one relevant situation occurred and the behavior was directly observed. Item 7 is scored from the number of qualifying episodes observed across the 48-hour interval (S5).
- Non-observability pathways: for items 1–6, S-NA is used when no relevant situation arose during the reporting interval. NO is used when one or more relevant situations arose, but the informant did not directly observe the participant's behavior. When the informant directly observed only some relevant situations, the rating is based solely on those observed situations and is not coded NO. A numeric rating of 1 is assigned when

a relevant situation arose, the behavior was directly observed and the criterion was not met. For item 7, S-NA does not apply because scoring is based on episode count rather than discrete opportunities. Zero qualifying episodes during an adequately observed interval produces a raw episode band of 1, which reverse-scores to 5; NO is used only when direct observation was insufficient to determine whether episodes occurred. TECH denotes a technical failure preventing form completion or data submission. TECH records are retained, excluded from aggregation and flagged for quality-control follow-up. NO and S-NA are also excluded from aggregation. Full item-level rules are provided in S5.

- Item polarity: items 1–6 positive; item 7 negative (reverse-scored) to maintain consistent directionality.

9 Functional domains and paired context-adapted items

DOMAIN A — Cognitive–Executive

A1. Initiates daily task on request;
A2. Maintains attention ≥5 min;
A3. Follows household instructions;
A4. Completes initiated activity;
A5. Resumes task after error;
A6. Switches activity on request;
A7. Non-functional interruption of tasks (reverse-scored).

DOMAIN B — Socio–Communicative

B1. Orients trunk and head toward the person speaking;
B2. Responds to gestures addressed to him or her within 3 s;
B3. Initiates an interaction with a family member;
B4. Waits for own turn in a shared exchange without interrupting;
B5. Assists family member on request;
B6. Maintains gaze toward the speaker ≥3 s;
B7. Withdraws from shared activity (reverse-scored).

DOMAIN C — Emotional–Motivational Regulation

C1. Remains engaged in the activity without leaving before it ends;
C2. Visible distress after an error or a correction subsides within about a minute;
C3. Engages with a changed plan without verbal or gestural refusal;
C4. Asks for help without raised voice, throwing, or leaving the place;
C5. During waiting intervals, remains in place without disruptive vocalization or motor disruption;
C6. Settles within about a minute after agitation appears;
C7. Sustained overt distress lasting >5 min (reverse-scored).

DOMAIN D — Motor–Sensorimotor

D1. Maintains posture and balance during the activity;
D2. Handles everyday objects with both hands without dropping them;
D3. Moves limb on request;
D4. Carries out a three-step task in the correct order;

D5. Adjusts force when handling delicate objects or in physical contact with another person;
D6. Moves safely in the environment;
D7. Repetitive non-functional movement (reverse-scored).

DOMAIN E — Synchronization

E1. Initiates action on signal;
E2. Maintains steady pace ≥10 s;
E3. Changes intensity on request;
E4. Re-synchronizes with others within 5 s;
E5. Synchronizes action with a partner;
E6. Changes speed on request;
E7. Loss of coordination or rhythm (reverse-scored).

10 Electronic administration and caregiver onboarding

- No fixed time limit is imposed after form opening; form-open and submission timestamps are retained, and completion duration is summarized prospectively as a feasibility outcome.
- Onboarding comprises a brief instructional video, a one-page reference sheet and a supervised practice submission before independent ObsRO-P reporting begins (S4).
- Data integrity: encrypted transmission, server-side timestamps, post-submission locking and audit logging are prespecified to support contemporaneous data capture, preservation of the original submitted record and traceability. Correct implementation will be verified through user-acceptance testing.

Supplementary Material S3

Standard operating procedures for rater calibration

1 Purpose and scope

This Standard Operating Procedure (SOP) defines the training and calibration procedures for clinician-reported ClinRO-T ratings.
It establishes structured processes to support:

- consistent interpretation of item anchors,
- stability of scoring across sessions,
- preservation of informant independence,
- traceability of calibration decisions.

The SOP applies to Psychologist A, the session-exposed primary rater, and Psychologist B, the independent secondary rater.

2 Roles and responsibilities

Psychologist A — session observer, distinct from the intervention provider

- Completes ClinRO-T within ≤24 hours after each scored OMT session.
- Participates in initial training and periodic re-calibration.
- Follows the scoring, non-observability and timing rules prespecified in S1 and S7.

Psychologist B — independent secondary rater

- Independently codes every scheduled ClinRO-T participant–session observation from the same pseudonymized 20-minute segment used by Psychologist A (target n = 100).
- Does not attend live sessions and has no intervention or clinical-management role.
- Uses a separate access path and cannot view Psychologist A's entries before both ratings are locked.
- Is masked to Psychologist A's ratings, ObsRO-P reports, Vineland-3 results, clinical records, treatment objectives and previous assessments. Masking to participant identity and program context is not claimed because these may be inferable from the recording.

OPBG Clinical Research Coordinator (CRC; to be appointed before study initiation)

- Is formally appointed and delegated by the principal investigator before study initiation and maintains the delegation, training, deviation and data clarification request (DCR) logs.
- Schedules training and refresher sessions and maintains attendance logs, calibration records, and version control for training materials.
- Will ensure consistent dissemination of updated anchors or clarifications and will verify that paired ClinRO-T ratings are locked before any discrepancy review.

3 Training content and schedule

Initial training (approx. 2 hours)

Includes:

- domain definitions and item anchors,
- non-observability pathways (NO, S-NA, TECH),
- scoring logic and reverse-scoring rules (S5/S7),
- examples illustrating boundary cases.

Video-based calibration

- Conducted using simulated 20-minute segments matching the ClinRO-T observational window.
- Calibration is anchored to expert musical annotation. For each scoring occasion, the annotation identifies the notated event, conductor cue and temporal window within which the behavioral criterion is counted as met. For items requiring perceptual comparison with the ensemble—entry on the beat, pulse maintenance,

resynchronization, dynamic change and coordination with the section—the criterion is expressed as an explicit tolerance relative to the conductor's downbeat or cue. Rater performance on these items is recorded separately during calibration, and item-level agreement will be evaluated empirically in phase 1.
- The calibration segments will be reconstructed specifically for training and concordance review. They will contain no footage or data from patients, study participants or actual sessions and will include representative and boundary-case scoring situations. Performance on these materials evaluates training adequacy and anchor discriminability; it is not an estimate of instrument reliability. Expert musical annotations guide identification of scoring occasions and do not constitute gold-standard behavioral ratings.
- Used to harmonize interpretation of anchors and response categories.

Training materials

- S1: ClinRO-T item bank and behavioral anchors
- S5: Observation grid, scoring opportunities and observable criteria
- S6: Variable Dictionary (metadata, QC variables)
- S7: Codebook and scoring logic

Refresher sessions

- Held every 8–10 sessions
- Triggered earlier if prespecified QC-review findings suggest potential drift.

4 Calibration procedure (video-assisted)

Step 1 — Independent rating

Psychologists A and B independently score the same simulated 20-minute segment using ClinRO-T.

Step 2 — Concordance review

Ratings are compared on discordant items.
Discussion focuses on:

- anchor interpretation,
- classification of observable opportunities and criterion-met occasions,
- ordinal-band and episode-count boundaries,
- boundary cases (e.g., transitions between categories).

Step 3 — Action log

A calibration log is completed including:

- session ID,
- item-level points of discussion,
- clarifications or examples agreed upon.

Logs are append-only, preserving traceability and version history.

5 Double-coding and concordance (descriptive only)

- Every scheduled ClinRO-T participant–session observation is independently coded by Psychologists A and B (target n = 100; 20 sessions × 5 designated targets). Any observation lacking two locked independent ratings is recorded as a protocol deviation and excluded from the primary inter-rater agreement set.
- Inter-rater agreement is summarized for the rater-determined item-level counts, the derived ordinal bands and the domain-level means, with repeated observations within participants addressed as specified in S10.
- Concordance between Psychologists A and B on the simulated calibration segments is computed before the first scored session, using the item-level and domain-level statistics specified in S10, and is reported alongside the phase 1 results. Because the material is simulated and constructed to contain boundary cases, this quantity characterizes training adequacy and anchor discriminability; it is not a reliability estimate for either instrument and does not substitute for the phase 1 agreement analysis.
- No phase 1 agreement estimate exists in the pre-implementation phase; those analyses are prospective and will be reported only after empirical data collection.
- Patterns of discrepancy identified after both records have been locked may trigger targeted feedback or micro-training.

6 Quality-control integration and data clarification requests

Quality-control monitoring

The OPBG Clinical Research Coordinator reviews the quality-control indicators derived from S6. Any review involving paired scoring patterns occurs only after both rater-specific records have been locked:

- Completion duration
- Submission latency
- Pattern anomalies
- Domain-score availability (at least 5 of 7 valid numeric ratings)
- TECH flags

These outputs populate a monitoring record used to identify potential scoring drift or procedural inconsistencies.

Data clarification request procedure

When anomalies are detected:

- The OPBG CRC issues a DCR noting the variable, anomaly and proposed clarification only after the relevant independent ratings have been locked.
- The rater responsible for the relevant entry provides the procedural clarification.
- All DCR resolutions are appended without overwriting the original entries, maintaining audit-trail integrity. DCR resolution is procedural and non-destructive: it

does not create a consensus score, alter either locked ClinRO-T rating, or merge the two rater-specific records.

7 Records and version control

Training attendance, calibration outcomes, action logs, and DCR resolutions are recorded with: date/time, P_ID, S_ID and rater ID.
Before form freeze, any update to anchors, examples or scoring rules is:

- version-controlled,
- time-stamped,
- communicated to all raters before the subsequent reporting cycle.

After form freeze, any substantive change requires a documented protocol amendment and applies prospectively. Historical versions remain accessible for audit purposes.

8 Occasion identification: score map and realized-event log

Identification of the scoring occasion is separated from the rating of behavior and is carried out by two roles that do not rate: the conductor–music therapist, who prepares the presession score map, and a musically competent annotator, who compiles the realized-event log after the session. Neither role has access to ClinRO-T or ObsRO-P entries.

Presession score map. For each scored work the conductor–music therapist will prepare, before the session, a map listing by measure the notated event, the conductor cue associated with it, the item code it generates and the observable criterion, with one row per planned opportunity. The map is version-controlled and frozen before the session, and the frozen version identifier is recorded with the session.

Realized-event log. After the session the annotator will compile against the recording a log of the events actually realized, recording for each planned opportunity the timestamp, the cue as produced, whether the passage was executed, omitted or repeated, and any restart or deviation. Repetitions generate additional opportunities and omissions void the planned opportunity. The log records events only and assigns no rating to any participant.

Freeze and release. Both layers are frozen before behavioral rating begins. Psychologists A and B then receive the same segment, score map, realized-event timestamps, criteria and temporal windows. Their independent ratings therefore use the same occasion information. An error in the score map or realized-event log would be shared by both raters rather than independent; the corresponding limitation is stated in sections 4.2 and 5.3 of the main manuscript.

Verification. Before the first scored session, the CRC will verify on the calibration material that each planned opportunity entered in the score map is identifiable in the recording and that the realized-event log reproduces the map when the passage is executed as planned. This verification concerns score-generated planned opportunities. Events without a presession antecedent are identified directly from the recording: performance errors that generate A5 opportunities, synchrony losses that generate E4 opportunities and qualifying item-7 episodes. Discrepancies will be resolved before behavioral rating begins and recorded in the action log.

Supplementary Material S4

Standard operating procedures for caregiver training

1 Purpose and scope

This Standard Operating Procedure (SOP) defines the onboarding workflow for caregivers who complete ObsRO-P. Objectives are to ensure:

- basic interface fluency,
- correct use of the NO/S-NA/TECH routes (no forced numeric responding), and
- adherence to the prespecified 48-hour post-session observation interval and submission deadline.

This SOP applies to all primary ObsRO-P informants. Before enrollment, one informant with no role in OMT delivery, clinical supervision, outcome rating or program volunteering is registered. The same informant is retained across the five target weeks. An unavoidable replacement completes the same onboarding procedure, receives a new pseudonymous informant identifier and generates the informant-change flag defined in S6. Affected records are retained and examined separately.

2 Roles and responsibilities

OPBG Clinical Research Coordinator (CRC; to be appointed before study initiation)

The CRC schedules onboarding, provides the materials, verifies completion of the supervised practice submission and maintains attendance logs and document version control. During data collection, the CRC may provide procedural support but neither suggests nor alters caregiver responses. The CRC conducts the prespecified response-process quality-control review on a randomly selected subset comprising 20% of submitted ObsRO-P forms (S6–S7) and initiates a data clarification request (DCR) when required. The review does not permit modification of the original submitted response. Psychologist B has no access to ObsRO-P records.

Caregiver (ObsRO-P reporter)

Completes the onboarding module and one supervised simulation submission; subsequently reports independently, using only behavior directly observed during the applicable 48-hour post-session interval and no second-hand account.

3 Onboarding materials

- Brief instructional video (≤5 minutes): outlines purpose, the 48-hour post-session observation interval, the five-category ordinal response scale, and the NO/S-NA/TECH taxonomy.
- One-page quick-reference sheet: definitions and examples distinguishing NO, S-NA, and TECH; reminder of the observation/submission interval; technical-support contact.

- Simulation form (web link): uses the same layout and routing logic as ObsRO-P and is rendered through Enketo. Practice submissions are not transferred to or stored in the production dataset.

4 Stepwise onboarding procedure

Step 1 — Orientation (10–15 min).

The OPBG CRC introduces ObsRO-P, explains the prespecified 48-hour post-session observation interval, and highlights non-observability routes. The caregiver watches the instructional video and receives the quick-reference sheet.

Step 2 — Supervised simulation (10–15 min).

The caregiver completes a practice submission using the simulation form. Scenarios include at least one case for each route (NO, S-NA, TECH) to verify correct interpretation and timing. The OPBG CRC confirms understanding and addresses procedural questions.

Step 3 — Confirmation and access (≤5 min).

Upon successful simulation, the caregiver receives the secure ObsRO-P link and a succinct reminder of:

- the 48-hour observation interval and submission deadline,
- confirmation that completion duration will be timestamped and evaluated descriptively,
- the independence of reporting (no consultation with the rating clinician or other informants).

5 Non-observability pathways

- NO (Not Observed) — For items 1–6, one or more relevant situations arose during the interval, but the caregiver did not directly observe the participant’s behavior. When the behavior was directly observed and the criterion was not met, the item receives a numeric rating of 1 rather than NO. No behavior is inferred from second-hand information or from periods outside the reporting interval.
- S-NA (Structural Not Applicable) — For items 1–6, no relevant situation calling for the behavior arose during the interval.
- Item 7 — S-NA does not apply because item 7 is scored from episode count rather than discrete opportunities. When the interval was adequately observed and no qualifying episode occurred, the caregiver enters zero episodes; the platform derives the corresponding raw episode band and reverse-scored value. NO is used only when direct observation was insufficient to determine whether qualifying episodes occurred.
- TECH (Technical Interruption) — A device or platform failure prevented form completion or submission.
- Operational effects: for items 1–6, selecting NO, S-NA or TECH disables numeric input. For item 7, S-NA is unavailable; selecting NO or TECH disables episode-count

entry. Non-numeric records are retained, excluded from aggregation and handled according to the quality-control workflow specified in S6–S7.

6 Data integrity and independence

- Independent channels: caregivers report via a segregated ObsRO-P link; they do not access clinician entries (ClinRO-T).
- Timing safeguards: server-side form-opening and submission timestamps and post-submission locking are prespecified to support contemporaneous data capture and preservation of the original submitted record. Correct implementation will be verified through user-acceptance testing. No fixed minimum or maximum completion duration is imposed. Completion duration is summarized prospectively as a feasibility outcome and does not independently determine record validity.
- Privacy and governance: submissions contain pseudonymous participant and session identifiers but no direct identifiers. The re-identification file remains separately under the control of the Data Controller (S11); the corresponding variable names are defined in S6.

7 Troubleshooting and support

- Access or display issues: verify link integrity, browser compatibility, and connectivity; if needed, re-issue a secure link.
- TECH events: if a technical interruption occurs, advise a second attempt within the window. If unresolved, record TECH and notify the OPBG CRC; the entry remains retained and QC-flagged.
- Clarifications: only procedural clarifications are provided (e.g., where to select NO/S-NA/TECH). No guidance is given on how to score specific behaviors to preserve response-process independence.

8 Records and version control

The OPBG CRC documents the onboarding date and time, practice-submission completion status and any procedural clarifications provided. Before form freeze, all onboarding materials—video, quick-reference sheet and simulation form—are version-controlled through an append-only change log, and updates are communicated before the subsequent onboarding cycle. After form freeze, any substantive change affecting instructions, response options or routing logic requires a documented protocol amendment and applies prospectively. Historical versions remain available for audit.

Supplementary Material S5

Observation grid, calibration and reverse-scoring guidance

9 Purpose and scope

This document specifies the current observation grid and the scoring rules applied to it. Part I defines, for each of the 35 items, the observable referent, the event or situation that permits scoring in each

channel and the applicable boundary or exclusion rules. Part II specifies item polarity, response bands, reverse-scoring, domain-score availability and boundary-case handling. Development history is documented separately in S12. Variable names and data types are specified in S6, algorithmic implementation in S7, rater calibration in S3 and the data-clarification workflow in S11.

10 Part I — Observation grid

The grid is the operational reference for the ClinRO-T and ObsRO-P item banks presented in S1 and S2. For each item, it distinguishes the behavioral criterion from the situation permitting that criterion to be scored and from conditions that invalidate or prevent scoring.

10.1 Opportunity-referenced scoring (items 1–6)

Items 1–6 use a five-category ordinal scale derived differently in the two channels. In ClinRO-T, the realized-opportunity count is supplied by the frozen realized-event log and is common to both raters. Each rater records how many realized opportunities were observable for the designated participant and on how many observable opportunities the behavioral criterion was met. The platform derives the category from the resulting proportion. In ObsRO-P, the caregiver selects an opportunity-conditioned frequency category based on directly observed relevant situations, without recording an enumerated denominator.

Three item-level routes apply to ClinRO-T items 1–6: when no opportunity occurred, the item is coded S-NA; when one or more opportunities occurred but none was observable, it is coded NO; when at least one opportunity was observable, the item is scored using the observable subset. The realized-opportunity count, observable-opportunity count and criterion-met count are retained separately.

The attainable ordinal categories depend on the number of observable opportunities. With one observable opportunity, only categories 1 and 5 are attainable; with two, only 1, 3 and 5 are attainable; with three, category 4 is unattainable; and at least four observable opportunities are required for all five categories to be attainable. Items with fewer than four observable opportunities remain scored and are reported with their denominators rather than suppressed. Denominator distributions are reported among the measurement-process indicators specified in S10.

The platform applies the following proportion bands: 5 = criterion met on ≥90% of observable opportunities; 4 = 70–89%; 3 = 40–69%; 2 = 10–39%; and 1 = <10%. These boundaries are prespecified design conventions. They will be reviewed using the calibration material before form freeze and evaluated descriptively after implementation; they are not presented as empirically established cutoffs.

In the orchestral setting, the score and conductor cues identify planned opportunities for several items in the Cognitive–Executive, Socio–Communicative and Synchronization domains. Before the session, these opportunities are entered in the presession score map. After the session, the realized-event log records whether each planned event was executed, omitted or repeated and identifies its timestamp. The realized-opportunity count is then prepopulated in the ClinRO-T form and locked. The rater enters only the observable-opportunity count and criterion-met count. The platform calculates the corresponding proportion and ordinal category. Exact variable names are defined in S6.

10.2 Episode-count scoring (item 7)

Item 7 records qualifying episodes rather than responses to discrete opportunities. In ClinRO-T, the raw episode bands for the 20-minute segment are: 1 = no episodes; 2 = one; 3 = two; 4 = three; and 5

= four or more. In ObsRO-P, the raw episode bands for the complete 48-hour interval are: 1 = no episodes; 2 = one or two; 3 = three to six; 4 = seven to twelve; and 5 = more than twelve. The platform applies the reverse transformation 6 − raw band.

The channels use different observation windows and episode-count boundaries. Item-level cross-context correspondence for item 7 must therefore be interpreted separately from correspondence for items 1–6. Within each domain, the reverse-scored item-7 band is combined without differential weighting with the six opportunity-referenced item scores. This is the prespecified scoring rule; empirical support for their commensurability has not been established.

10.3 Decision rule for non-observability

NO, S-NA and TECH are mutually exclusive item-level non-numeric response options. They do not include uncoded missing items or whole-form non-submission, which are handled separately.

For items 1–6, S-NA is used when no relevant opportunity or situation arose. NO is used when one or more opportunities arose but none was directly observable. In ClinRO-T, this may result from absence, occlusion or recording position. In ObsRO-P, NO applies when a relevant situation arose but the caregiver did not directly observe the participant's behavior. When only some relevant opportunities or situations were observed, scoring is based on the observed subset. A numeric rating of 1 is assigned when the participant was observed and the criterion was not met.

S-NA does not apply to item 7 because episode counting does not require a discrete opportunity. Zero qualifying episodes during an adequately observed window produces a raw episode band of 1 and a reverse-scored value of 5. NO is used only when observation was insufficient to determine whether qualifying episodes occurred. TECH is reserved for a technical failure affecting observation, form completion or submission, as applicable to the channel. Non-numeric responses are retained, excluded from aggregation and handled under the quality-control rules in S6–S7.

10.4 Differences between the channels

ClinRO-T uses replayable video, permitting enumeration of scoring opportunities and verification of second-level temporal criteria. ObsRO-P relies on direct caregiver observation across 48 hours; opportunities cannot be exhaustively enumerated and fine temporal thresholds cannot be verified in the same manner. The channels share the ordinal range, scoring direction and non-observability pathways, but their response processes and measurement resolution differ. No metric equivalence is assumed.

For Domains A, B and D, the paired items concern functionally corresponding behaviors assessed at different levels of observational resolution. Domain E differs more substantially: ClinRO-T assesses synchronization relative to a written pulse and performing partners, whereas ObsRO-P assesses pacing and coordination with another person in daily life. Domain E correspondence is therefore more context-dependent and cannot be interpreted as agreement between interchangeable measurements.

10.5 Part and repertoire

Opportunity density and task difficulty may differ across instrumental parts and across the selected passages. The repertoire and each participant's personalized part remain fixed across the 20-week period, but this does not make opportunity counts equivalent between orchestral sections. Target rotation is stratified by orchestral section, and the scored piece, passage and assigned part are recorded with each segment.

10.6 Domain A — Cognitive–Executive

Item	Observable referent and criterion	ClinRO-T — opportunity and stem (20-min OMT segment)	ObsRO-P — opportunity and stem (≤48 h daily-life window)	Boundary and exclusions
A1	Task-directed action initiated within 5 s of an external initiating cue.	Opportunity: a start cue written in the part and given by the conductor. Stem: begins playing or preparatory action within 5 s of the cue.	Opportunity: an adult's request to begin a defined task. Stem: begins the requested task promptly, without repetition of the request.	No cue in the segment → S-NA; participant out of frame → NO.
A2	Task-directed attention sustained ≥5 min with no off-task interruption >10 s.	Opportunity: a continuous rehearsal block of ≥5 min. Stem: sustains attention throughout the block.	Opportunity: a daily activity lasting ≥5 min. Stem: sustains attention to the activity for at least five minutes.	No block of ≥5 min → S-NA. Interruption instructed by the conductor is not off-task.
A3	Ongoing action modified after a change of instruction, within 10 s.	Opportunity: a mid-passage change of instruction (tempo, dynamics, entry point, articulation). Stem: adjusts within 10 s.	Opportunity: a change of instruction for an activity under way. Stem: adjusts without the instruction being repeated more than once.	Distinct from A6, which is transition between tasks. A conductor correction is one instance of a change of instruction and therefore constitutes an A3 opportunity as well as a C3 opportunity. No change of instruction → S-NA.
A4	Assigned unit of activity carried to its defined endpoint without abandonment.	Opportunity: an assigned passage with a written endpoint. Stem: plays the passage through to its end.	Opportunity: an activity begun with a defined endpoint. Stem: carries the activity through to its end.	External interruption voids the opportunity → S-NA for that occurrence.
A5	Task-directed action resumed within 5 s of a performance error.	Opportunity: an audible error. Stem: resumes playing within 5 s.	Opportunity: an error during a daily task. Stem: returns to the task promptly without abandoning it.	The same event may generate an opportunity for C2; A5 scores latency, C2 scores overt distress.

Item	Observable referent and criterion	ClinRO-T — opportunity and stem (20-min OMT segment)	ObsRO-P — opportunity and stem (≤48 h daily-life window)	Boundary and exclusions
A6	Transition to a different activity begun within 10 s of the request.	Opportunity: rehearsal moves to another section or piece. Stem: moves to the new section within 10 s.	Opportunity: a request to change activity. Stem: changes activity without the request being repeated more than once.	Transition instructed but materially impossible → S-NA.
A7 (reverse)	Task stopped or abandoned without task-consistent reason. Metric: episode count.	Stem: episodes of stopping or abandoning the task without task-consistent reason.	Stem: episodes of stopping or abandoning a task without apparent reason.	Stopping on instruction, or after an external or technical interruption, is excluded.

10.7 Domain B — Socio–Communicative

Item	Observable referent and criterion	ClinRO-T — opportunity and stem (20-min OMT segment)	ObsRO-P — opportunity and stem (≤48 h daily-life window)	Boundary and exclusions
B1	Trunk and head oriented toward the person directing the activity when addressed.	Opportunity: the conductor addresses the ensemble or the section. Stem: orients trunk and head toward the conductor.	Stem: orients trunk and head toward the person speaking.	Instrument prevents trunk rotation → S-NA; participant out of frame → NO.
B2	Behavioral response to a non-verbal gesture within 3 s.	Opportunity: a conductor gesture addressed to the participant or section. Stem: responds within 3 s.	Stem: responds to gestures addressed to him or her.	Gesture outside the visual field → S-NA; gesture simultaneous with a verbal instruction → excluded.
B3	Task-relevant exchange initiated with a peer or family member.	Opportunity: intervals permitting peer exchange. Stem: initiates a task-relevant exchange with a peer in the section.	Stem: initiates an interaction with a family member.	Exchange initiated by the other party, with the participant only responding, does not count.
B4	Turn-taking: waiting for one’s own turn	Opportunity: passages assigned to	Stem: waits for own turn in a shared	Responsiveness to being addressed

Item	Observable referent and criterion	ClinRO-T — opportunity and stem (20-min OMT segment)	ObsRO-P — opportunity and stem (≤48 h daily-life window)	Boundary and exclusions
	without intruding on another's.	another section. Stem: waits for own entry without playing during another section's passage.	exchange without interrupting.	remains covered by B2 and B6.
B5	Assistance provided to another person on request.	Opportunity: a request for help from a peer or the conductor. Stem: assists a peer when asked.	Opportunity: a request for help from a family member. Stem: assists a family member when asked.	Assistance offered without request is recorded under B3.
B6	Gaze directed toward the person directing the activity, sustained ≥3 s.	Opportunity: moments of visual cueing by the conductor. Stem: maintains gaze toward the conductor for ≥3 s.	Stem: maintains gaze toward the speaker for ≥3 s.	Gaze not assessable from the camera angle → NO. Gaze direction is scored, not attention.
B7 (reverse)	Persistent disengagement from the shared activity, active or passive. Metric: episode count.	Stem: episodes of withdrawal from the group activity.	Stem: episodes of withdrawal from shared activities.	Non-participation during a passage not assigned to the participant is not withdrawal.

10.8 Domain C — Emotional–Motivational Regulation

All Domain C items are scored from explicitly defined behavior. No item requires the rater or caregiver to infer an internal affective state.

Item	Observable referent and criterion	ClinRO-T — opportunity and stem (20-min OMT segment)	ObsRO-P — opportunity and stem (≤48 h daily-life window)	Boundary and exclusions
C1	Remaining at one's own station, in the required posture, for the duration of the assigned unit.	Stem: remains at own station in playing posture for the duration of the passage; leaves only when instructed.	Stem: remains engaged in the activity without leaving before it ends.	Leaving on instruction or for a physiological need is excluded.

Item	Observable referent and criterion	ClinRO-T — opportunity and stem (20-min OMT segment)	ObsRO-P — opportunity and stem (≤48 h daily-life window)	Boundary and exclusions
C2	Overt distress behavior after an error or correction—raised voice, object thrown or struck, hands over ears, leaving the seat or crying—ends within 60 s or does not occur.	Opportunity: an audible error, or a correction addressed to the participant. Stem: any overt distress ends within 60 s.	Stem: after an error or correction, any visible distress subsides within about a minute.	Observable behavior is rated, not inferred affect. No error or correction → S-NA. Distress beyond 5 min is additionally recorded under C7.
C3	Engagement with an externally imposed correction or change, without verbal refusal, gesture of refusal or cessation of the activity.	Opportunity: a correction from the conductor. Stem: attempts the corrected action without refusal and without stopping.	Stem: after a change of plan, engages with the new arrangement without refusal.	Attempting and failing counts as engagement; refusal behavior is scored, not performance.
C4	Assistance requested by speech, sign, gesture or device, without raised voice, throwing or leaving the place.	Stem: requests assistance without raised voice, throwing, or leaving the seat.	Stem: asks for help without raised voice, throwing, or leaving the place.	Not requesting help where help was needed scores 1 for that opportunity; it is not NO.
C5	During non-active intervals ≥30 s, remaining in place and oriented to the shared activity, without disruptive vocalization or non-task motor disruption.	Opportunity: intervals ≥30 s in which the participant is not playing. Stem: remains seated and oriented to the ensemble.	Stem: during waiting intervals in daily routines, remains in place without disruption.	For ClinRO-T, an interval shorter than 30 s does not create a C5 scoring opportunity.
C6	After the onset of agitation signs—raised voice, rapid repetitive movement, leaving the place or non-task object manipulation—return to seated,	Stem: after agitation signs appear, returns to task-oriented posture within 60 s.	Stem: after agitation appears, settles within about a minute.	Agitation is defined by the listed signs and by nothing else. No onset → S-NA.

Item	Observable referent and criterion	ClinRO-T — opportunity and stem (20-min OMT segment)	ObsRO-P — opportunity and stem (≤48 h daily-life window)	Boundary and exclusions
	task-oriented posture within 60 s.			
C7 (reverse)	Episode of sustained overt distress—crying, screaming, self- or object-directed aggression or leaving the room—lasting >5 min and not resolved by brief self-regulatory attempts. Metric: episode count.	Stem: episodes of sustained overt distress lasting more than five minutes.	Stem: episodes of sustained overt distress lasting more than five minutes.	An episode >5 min consumes a quarter of the segment; record it in the calibration notes with its effect on the other domains.

10.9 Domain D — Motor–Sensorimotor

Item	Observable referent and criterion	ClinRO-T — opportunity and stem (20-min OMT segment)	ObsRO-P — opportunity and stem (≤48 h daily-life window)	Boundary and exclusions
D1	Required posture maintained without loss of position needing external correction.	Stem: maintains instrumental posture throughout the passage.	Stem: maintains posture and balance during the activity.	Posture corrected on instruction is not a failure unless the correction must be repeated.
D2	Two hands, or limb and trunk, coordinated in the required action without loss of the object or of position.	Stem: coordinates the two hands in the required instrumental action.	Stem: handles everyday objects with both hands without dropping them.	For single-hand instruments substitute limb–trunk coordination and record the substitution.
D3	Requested limb moved within 3 s of an external signal.	Stem: moves the requested limb within 3 s of the signal.	Stem: moves the requested limb on request.	Overlaps with B2 when the signal is gestural; B2 scores social responsiveness, D3 motor execution.

Item	Observable referent and criterion	ClinRO-T — opportunity and stem (20-min OMT segment)	ObsRO-P — opportunity and stem (≤48 h daily-life window)	Boundary and exclusions
D4	Motor sequence of ≥3 elements executed in the correct order.	Opportunity: a written instrumental passage requiring a prespecified motor sequence of ≥3 elements. Stem: executes the required motor sequence in the correct order.	Stem: carries out a three-step task in the correct order.	Timing errors without order errors are scored under Domain E.
D5	Force applied to an object modulated to the level the task requires.	Stem: regulates pressure and touch on the instrument to the written dynamic, without excess.	Stem: adjusts force when handling delicate objects or during physical contact with another person.	—
D6	Movement through the environment during transitions without collision, fall, or contact requiring correction.	Stem: moves safely during transitions.	Stem: moves safely in the environment.	Environmental hazards outside the participant's control are excluded.
D7 (reverse)	Repetitive movement without task relevance. Metric: episode count.	Stem: episodes of repetitive non-functional movement.	Stem: episodes of repetitive non-functional movement.	Movement required by the instrumental technique is excluded; a self-regulatory function is noted, not recoded.

10.10 Domain E — Synchronization

Item	Observable referent and criterion	ClinRO-T — opportunity and stem (20-min OMT segment)	ObsRO-P — opportunity and stem (≤48 h daily-life window)	Boundary and exclusions
E1	Action initiated on the external temporal signal, within one beat.	Opportunity: the entries written in the participant's part. Stem: enters on the beat at the assigned entry.	Stem: starts the action on the signal, together with the others.	An entry re-cued individually after a missed entry is a failure of that opportunity.

Item	Observable referent and criterion	ClinRO-T — opportunity and stem (20-min OMT segment)	ObsRO-P — opportunity and stem (≤48 h daily-life window)	Boundary and exclusions
E2	Stable pace maintained ≥10 s without external correction.	Opportunity: passages of ≥10 s with a stable written pulse. Stem: maintains the rhythm for at least 10 s.	Stem: maintains a steady pace for at least 10 s.	Passages with a written tempo change are excluded and scored under E6.
E3	Intensity or dynamic level changed on signal, within one bar.	Opportunity: the dynamic changes written in the part. Stem: changes dynamics on the signal.	Stem: changes intensity on request.	Change not audible from the recording → NO.
E4	Return to synchrony within 5 s of a detected loss.	Stem: re-synchronizes within 5 s of losing the beat.	Stem: gets back in step with the others quickly.	No loss of synchrony → S-NA; this is not a failure and must not be coded NO.
E5	Own action temporally aligned with that of the section or a partner over ≥10 s.	Stem: synchronizes with the section.	Stem: synchronizes actions with a partner.	If the section itself loses synchrony, the opportunity is void and coded S-NA. A passage that does not provide at least 10 s of potentially aligned action also does not constitute an opportunity. Passages selected as E5 opportunities must exceed this duration according to the notated tempo, and their realized duration is verified in the realized-event log.
E6	Speed adjusted on request within one bar and maintained ≥5 s.	Opportunity: the tempo changes written in the part. Stem: changes tempo on request.	Stem: changes speed on request.	Adjusting and immediately reverting is a failure of that opportunity.

Item	Observable referent and criterion	ClinRO-T — opportunity and stem (20-min OMT segment)	ObsRO-P — opportunity and stem (≤48 h daily-life window)	Boundary and exclusions
E7 (reverse)	Loss of rhythm or coordination not attributable to external cues or technical faults. Metric: episode count.	Stem: episodes of loss of rhythm.	Stem: episodes of loss of coordination or rhythm.	Losses caused by a section error or by a conductor cue are excluded.

ClinRO-T opportunity taxonomy

For ClinRO-T items 1–6, the grid distinguishes a planned opportunity from a realized opportunity. A planned opportunity is entered in the presession score map from the score and conductor cue. The realized-event log records whether that event occurred, was omitted or was repeated and identifies its timestamp and any restart or deviation. Repetitions generate additional realized opportunities; omissions void the corresponding planned opportunity. A realized opportunity enters the scoring denominator only when the designated participant was observable.

Some scoring events have no presession score-map antecedent and are identified directly from the recording. Examples include performance errors that generate A5 and, where applicable, C2 opportunities; corrections issued during the session that generate applicable A3, C2 or C3 opportunities; and detected losses of synchrony that generate E4 opportunities. Item 7 is treated separately: qualifying episodes are recorded in the realized-event log but are not classified as opportunities and do not use an opportunity denominator. Item-specific rules remain authoritative.

Item-level classification. Each item carries two prespecified classifications, fixed before data collection and independent of domain assignment. The first is the origin of the scoring opportunity, score-determined or endogenous, as stated in section 3.1.9 of the main manuscript: a score-determined opportunity is entered in the presession score map and verified in the realized-event log, whereas an endogenous opportunity arises from an unplanned event and is identified from the recording alone. The second is the principal observational requirement, which determines the perceptual demand placed on the rater and is reported as a secondary stratum in the phase 1 agreement analysis (S10). Because perceptual-musical demands occur in several domains, agreement is reported by item and, secondarily, by observational-demand class.

Item	Opportunity origin	Observational-demand class
A1	Score-determined	Cue-response
A2	Score-determined	Score-location
A3	Score-determined	Cue-response
A4	Score-determined	Score-location
A5	Endogenous	Spontaneous-event
A6	Score-determined	Cue-response
A7	Endogenous	Episode-count
B1	Score-determined	Cue-response
B2	Score-determined	Cue-response
B3	Score-determined	Score-location

Item	Opportunity origin	Observational-demand class
B4	Score-determined	Score-location
B5	Endogenous	Spontaneous-event
B6	Score-determined	Cue-response
B7	Endogenous	Episode-count
C1	Score-determined	Score-location
C2	Endogenous	Spontaneous-event
C3	Endogenous	Spontaneous-event
C4	Endogenous	Spontaneous-event
C5	Score-determined	Score-location
C6	Endogenous	Spontaneous-event
C7	Endogenous	Episode-count
D1	Score-determined	Score-location
D2	Score-determined	Motor-sequence
D3	Score-determined	Cue-response
D4	Score-determined	Motor-sequence
D5	Score-determined	Motor-sequence
D6	Score-determined	Score-location
D7	Endogenous	Episode-count
E1	Score-determined	Temporal-synchronization
E2	Score-determined	Temporal-synchronization
E3	Score-determined	Temporal-synchronization
E4	Endogenous	Temporal-synchronization
E5	Score-determined	Temporal-synchronization
E6	Score-determined	Temporal-synchronization
E7	Endogenous	Episode-count

Twenty-three items are score-determined and twelve endogenous. By observational-demand class: eight score-location, seven cue-response, six temporal-synchronization, six spontaneous-event, three motor-sequence and five episode-count items. Two boundary cases are recorded. A3 is classified score-determined because changes of instruction written in the part are entered in the score map; a conductor correction is an endogenous instance of the same referent and, when it occurs, is logged as such in the realized-event log. C3 is classified endogenous because its only eliciting condition is a correction, which has no presession antecedent.

1 Part II — Scoring, reverse-scoring and boundary cases

This part specifies scoring direction, reverse-scoring of item 7, domain-score availability and the handling of mixed observations within a reporting window.

2 Item polarity and rationale

Items 1–6 are scored so that higher values indicate more frequent occurrence of the specified target behavior. Item 7 records disruptive or non-functional episodes and is reverse-scored so that all final item scores have the same direction. This is a scoring convention and does not imply clinical benefit.

3 Reverse-scoring specification

Applicability

Reverse-scoring applies only to item 7 in each domain (A7, B7, C7, D7 and E7) and only when a numeric raw episode band from 1 to 5 is available. NO and TECH are not reverse-scored and do not contribute to aggregation. S-NA is unavailable for item 7.

Transformation

For a numeric raw episode band:
Reversed score = 6 − raw episode band

Raw episode band	Reversed score
1	5
2	4
3	3
4	2
5	1

Items 1–6 are not reverse-scored. Exact variable names and derivation syntax are specified in S6–S7.

Handling non-numeric entries

For items 1–6, NO, S-NA and TECH do not produce a numeric item score and are excluded from aggregation. For item 7, the corresponding non-numeric routes are NO and TECH; S-NA is not available. TECH records are retained for feasibility and traceability and are flagged for quality-control follow-up (S6–S7).

4 Domain-level aggregation and score availability

A domain mean is computed only when at least 5 of its 7 items have valid numeric scores. This is the domain-score availability rule and is applied identically to ClinRO-T and ObsRO-P. When the rule is not met, the domain mean is not computed and the corresponding quality-control flag is raised. NO, S-NA and TECH do not contribute numerically.
For each record, the number of available domain means is reported as an integer from 0 to 5. This is a data-availability indicator; a value of 0 is an observed indicator value and is not itself an uncoded missing value. No cross-domain composite is computed in phase 1. Exact variable names and implementation rules are provided in S6–S7.

5 Boundary-case guidance

The observation grid specifies the item-level conditions that invalidate an opportunity or exclude a putative occurrence. Those definitions are not repeated here.
Mixed observations within a window are handled according to the item-specific scoring process rather than by selecting a predominant pattern. For ClinRO-T items 1–6, every observable realized opportunity contributes to the counts, and the platform derives the ordinal category from the resulting proportion. For ObsRO-P items 1–6, the caregiver selects the opportunity-conditioned frequency category across directly observed relevant situations. Item 7 is scored using the channel-specific

episode-count bands. Boundary cases identified during calibration are recorded in the calibration documentation described in S3.

Related specifications

Rater calibration against this grid, including concordance review and drift monitoring, is specified in S3. Quality-control flags and the data-clarification workflow are specified in S6 and S11. Prohibited practices for the pre-implementation phase are listed in S3 and S9.

Supplementary Material S6

Variable dictionary

1 Purpose and scope

This document specifies the variable names, data types, metadata fields, derived indicators and quality-control (QC) flags for the longitudinal dataset generated by the dual-informant COA system comprising two other-report instruments: ClinRO-T, classified as a clinician-reported outcome, and ObsRO-P, classified as a caregiver observer-reported outcome.
The schema will be configured to support traceability, contemporaneous data capture and preservation of original records. Correct implementation will be verified through user-acceptance testing. These specifications do not establish formal regulatory compliance or constitute validation of the electronic system. The schema preserves alignment with the administration, scoring and governance procedures prespecified in the main manuscript.

2 Primary identifiers and submission metadata

Privacy and governance

The analytical dataset contains no direct identifiers. The pseudonymous P_ID, session identifier S_ID and re-identification file are governed separately; the re-identification file and any direct identifiers are held exclusively by the Data Controller (see S11).

Variable	Type	Description / operational logic
P_ID	String (alphanumeric)	Participant Identifier; pseudonymous study key (no direct identifiers).
S_ID	Integer	Session Identifier (1–20) for the OMT session associated with the COA entry.
COA_TYPE	Categorical	Informant channel: ClinRO-T (clinician channel) or ObsRO-P (caregiver channel).
RATER_ID	Categorical or Null	ClinRO-T rater: Psychologist A or Psychologist B. Null for ObsRO-P.

Variable	Type	Description / operational logic
SESSION_END_TS	Timestamp (ISO 8601)	End time of the corresponding OMT session; temporal reference for the ClinRO-T deadline and ObsRO-P reporting interval.
FORM_OPEN_TS	Timestamp (ISO 8601)	Server-side time at which the electronic form was opened.
SUB_TS	Timestamp (ISO 8601)	Server-side finalization time of the submission; post-submission locking, time-zone handling and timestamp capture will be verified through user-acceptance testing.
SUBMISSION_LATENCY_HOURS	Float (hours)	Elapsed time from SESSION_END_TS to SUB_TS; used to assess the ≤24-hour ClinRO-T deadline and ObsRO-P submission by the end of the 48-hour interval.
COMPLETION_DURATION_MIN	Float (minutes)	Elapsed time from FORM_OPEN_TS to SUB_TS; summarized descriptively as a feasibility measure.
SEGMENT_ID	String or Null	Identifier of the 20-minute recording segment used for ClinRO-T; Null for ObsRO-P.
SEGMENT_POSITION	Categorical or Null	Opening, middle or closing segment; Null for ObsRO-P.
ORCHESTRAL_SECTION	Categorical	Participant's orchestral section for the scored session.
ASSIGNED_PART_ID	String	Version-controlled identifier of the participant's assigned instrumental part.
RECORDING_CONFIG_ID	String or Null	Identifier linking the ClinRO-T segment to the recording-configuration log; Null for ObsRO-P.
SCORE_MAP_VERSION	String or Null	Frozen version of the presession score map; Null for ObsRO-P.
REALIZED_EVENT_LOG_VERSION	String or Null	Frozen version of the realized-event log; Null for ObsRO-P.

Computation notes

- SUBMISSION_LATENCY_HOURS = (SUB_TS − SESSION_END_TS) / 3600
- COMPLETION_DURATION_MIN = (SUB_TS − FORM_OPEN_TS) / 60

All timestamps are recorded in ISO 8601 format with a consistent time-zone offset and retained in the audit trail.

3 Item-level variables (observational data)

Each domain contains 7 items. Items 1–6 are positively scored; item 7 is scored from episode count and reverse-scored during processing (S5, S7).

Naming convention

- [DOM][ITEM]_RAW — Ordinal raw band or applicable non-numeric response code. For ClinRO-T items 1–6, the ordinal band is derived from the recorded counts. For ObsRO-P items 1–6, it is selected by the caregiver. For item 7, it is derived from the episode count.
- [DOM][ITEM]_SCORE — Rule-derived numeric score after prespecified transformations.
- [DOM][ITEM]_OPP_REALIZED — ClinRO-T items 1–6 only: number of scoring opportunities actually realized in the scored segment, derived from the frozen realized-event log (integer ≥ 0; Null for ObsRO-P and for item 7). Identical for both raters, prepopulated in the form and locked; it is not a rater judgment and is not treated as an independently rated quantity. The planned opportunities of the score map are documented in S5 and do not enter the rater denominator.
- [DOM][ITEM]_OPP_OBSERVABLE — ClinRO-T items 1–6 only: number of realized opportunities on which the participant was observable (integer ≥ 0, ≤ _OPP_REALIZED). Rater-determined.
- [DOM][ITEM]_MET — ClinRO-T items 1–6 only: number of observable opportunities on which the criterion was met (integer ≥ 0, ≤ _OPP_OBSERVABLE). [DOM][ITEM]_RAW for these items is derived server-side as _MET / _OPP_OBSERVABLE mapped onto the proportion bands of S5, and is not entered by the rater.
- [DOM]7_EPISODE_COUNT — Item 7 only: number of qualifying episodes observed during the applicable channel-specific window. Integer ≥0; Null when NO or TECH applies. The platform derives [DOM]7_RAW from the episode-count bands specified in S5.

Additional canonical variables

- RECRUITMENT_SOURCE — categorical: spontaneous APS interest, OPBG referral, ASL Roma 4 referral.
- INF_ID — pseudonymous identifier of the ObsRO-P informant; a new identifier is issued on any replacement.
- INF_RELATION_CLASS — categorical: parent, other family member, regular support person.
- INFORMANT_CHANGE — {0,1}; 1 where the informant differs from the one used at the participant's previous target week.
- FORM_VERSION and FORM_DATE — version identifier and freeze date of the deployed form, recorded on every submission.
- [DOM]_ITEMS_USED — identity of the items contributing to each computed domain mean, because the ≥5-of-7 rule permits a mean to be computed over different item subsets on different occasions.

Domain prefixes

- A = COG = Cognitive–Executive
- B = SOC = Socio–Communicative
- C = EMO = Emotional–Motivational Regulation
- D = MOT = Motor–Sensorimotor
- E = SYN = Synchronization

Domain letters and variable prefixes map one-to-one (A → COG, B → SOC, C → EMO, D → MOT, E → SYN). Item variables use the word prefix (for example, the item labelled C7 in the item bank is the variable EMO7); the letters A–E are retained only as item identifiers and are never used as variable prefixes.

Allowed values and handling

Variable	Type	Allowed values / handling
[DOM][ITEM]_RAW, items 1–6	Categorical	{1, 2, 3, 4, 5, NO, S-NA, TECH}. For ClinRO-T, the numeric band is derived from OPP_OBSERVABLE and MET. For ObsRO-P, the numeric category is entered directly.
[DOM]7_EPISODE_COUNT	Integer or Null	Integer ≥0 when the interval was adequately observed; Null when NO or TECH applies.
[DOM]7_RAW	Categorical	{1, 2, 3, 4, 5, NO, TECH}. Numeric bands are derived from EPISODE_COUNT. S-NA is not permitted.
[DOM][ITEM]_SCORE	Integer (1–5) or Null	If RAW is numeric, items 1–6 retain the raw band and item 7 is reverse-scored. If RAW is NO, S-NA or TECH, as applicable to the item, SCORE is Null.

Non-observability taxonomy

- NO — For items 1–6, at least one relevant opportunity or situation arose but none was directly observable. For item 7, observation was insufficient to determine whether qualifying episodes occurred. Excluded from aggregation.
- S-NA — No relevant opportunity or situation arose. Applies only to items 1–6 and is excluded from aggregation.
- TECH — A technical interruption or device/platform error affected observation, form completion or submission, as applicable to the channel. Retained, excluded from aggregation and flagged for quality-control follow-up.

4 Derived and aggregated indicators

A domain mean is computed only when at least 5 of its 7 items have valid numeric scores. This is the domain-score availability rule.

Variable	Type	Computation	Availability condition
COG_MEAN	Float or Null	Arithmetic mean of valid COG item scores (1–5; after reverse-scoring for item 7).	Null if <5/7 valid numeric items
SOC_MEAN	Float or Null	Mean of valid SOC item scores (1–5).	Null if <5/7
EMO_MEAN	Float or Null	Mean of valid EMO item scores (1–5).	Null if <5/7
MOT_MEAN	Float or Null	Mean of valid MOT item scores (1–5).	Null if <5/7
SYN_MEAN	Float or Null	Mean of valid SYN item scores (1–5).	Null if <5/7
N_AVAILABLE_DOMAINS	Integer	Count of domain means satisfying the 5-of-7 domain-score availability rule.	Range 0–5; data-availability indicator only

Derivation logic. The reverse-scoring transformation, the domain-mean computation and the exclusion of NO, S-NA and TECH from aggregation are specified once in S7 and are not restated here. S6 defines variable names, data types and permitted ranges only.

5 Quality-control flags (binary; 0 = pass, 1 = flag)

Quality-control flags are generated automatically to support monitoring and the data clarification request (DCR) workflow. They accompany the source record and never overwrite the original entry.

QC flag	Trigger logic	System variable(s)	Primary response
QC_WINDOW_NONCOMPLIANT	SUBMISSION_LATENCY_HOURS >24 h (ClinRO-T) or >48 h (ObsRO-P).	SUBMISSION_LATENCY_HOURS	Retain in the all-submitted dataset; exclude from the primary window-compliant analysis set and retain for sensitivity summaries.

QC flag	Trigger logic	System variable(s)	Primary response
QC_PATTERN_ANOMALY	Invariant or straight-line response patterns.	PATTERN_TYPE (platform-derived classification)	Initiate a descriptive response-process review. The flag does not independently invalidate the record.
QC_TECH_FLAG	Any TECH selection encountered.	ITEM_TECH_FLAG	Exclude the item from aggregation; device troubleshooting if needed.
QC_DOMAIN_UNAVAILABLE	Fewer than 5 of 7 numeric item scores within a domain.	DOMAIN_VALID_ITEMS_N	Domain mean not computed; availability flag retained.
QC_INCOMPLETE	The item has neither a numeric response nor an applicable NO, S-NA or TECH code.	ITEM_COMPLETE_FLAG	DCR to clarify nature of omission.
QC_REVIEW_TRIGGER	20% of submitted ObsRO-P forms randomly selected for response-process quality-control review.	REVIEW_STATUS	Prespecified response-process QC review.

Implementation notes

- PATTERN_TYPE categories may include: invariance, highly repetitive alternation, or other platform-specific anomaly classes.
- DOMAIN_VALID_ITEMS_N records the number of numeric item scores available within the domain; at least 5 are required to compute the domain mean.
- Window violations (QC_WINDOW_NONCOMPLIANT = 1) do not delete records; they are retained and may be excluded from primary analyses per protocol.

- Selection into QC_REVIEW_TRIGGER is performed without reference to the caregiver's score values and does not indicate an anomalous response.

6 Data types, ranges, and coding conventions

Variable	Type	Range / levels	Notes
P_ID	String	—	Pseudonymous participant key; no direct identifiers in the analytical dataset.
S_ID	Integer	1–20	Session sequence number.
COA_TYPE	Categorical	{ClinRO-T, ObsRO-P}	Informant separation is maintained end-to-end.
RATER_ID	Categorical / Null	{A, B, Null}	ClinRO-T rater; Null for ObsRO-P.
RECRUITMENT_SOURCE	Categorical	{APS_SELF_REFERRAL, OPBG_REFERRAL, ASL4_REFERRAL}	Recruitment-source classification.
INF_ID	String / Null	—	Pseudonymous ObsRO-P informant identifier; Null for ClinRO-T.
INF_RELATION_CLASS	Categorical / Null	{parent, other family member, regular support person, Null}	ObsRO-P informant relationship; Null for ClinRO-T.
INFORMANT_CHANGE	Binary / Null	{0, 1, Null}	ObsRO-P informant-change flag; Null for ClinRO-T.
SESSION_END_TS	Timestamp	ISO 8601	Temporal reference for reporting-window calculations.
FORM_OPEN_TS	Timestamp	ISO 8601	Server-side form-opening time.
SUB_TS	Timestamp	ISO 8601	Server-finalized, post-submission locked and audit-logged as configured; correct implementation will be verified through user-acceptance testing.

Variable	Type	Range / levels	Notes
SUBMISSION_LATENCY_HOURS	Float	≥0	Hours from SESSION_END_TS to SUB_TS.
COMPLETION_DURATION_MIN	Float	≥0	Minutes from FORM_OPEN_TS to SUB_TS.
SEGMENT_ID	String / Null	—	ClinRO-T recording-segment identifier; Null for ObsRO-P.
SEGMENT_POSITION	Categorical / Null	{opening, middle, closing, Null}	Prespecified within-session segment position.
ORCHESTRAL_SECTION	Categorical	Prespecified section categories	Orchestral section used for sampling stratification and interpretation.
ASSIGNED_PART_ID	String	—	Version-controlled identifier of the assigned instrumental part.
RECORDING_CONFIG_ID	String / Null	—	Links ClinRO-T records to the recording-configuration log; Null for ObsRO-P.
SCORE_MAP_VERSION	String / Null	—	Frozen score-map version; Null for ObsRO-P.
REALIZED_EVENT_LOG_VERSION	String / Null	—	Frozen realized-event-log version; Null for ObsRO-P.
FORM_VERSION	String	—	Version of the administered electronic form.
FORM_DATE	Date	ISO 8601 date	Form-freeze or effective date.
[DOM][ITEM]_OPP_REALIZED	Integer / Null	≥0 or Null	ClinRO-T items 1–6 only; supplied by the frozen realized-event log.
[DOM][ITEM]_OPP_OBSERVABLE	Integer / Null	0 to OPP_REALIZED or Null	ClinRO-T items 1–6 only; rater-determined.
[DOM][ITEM]_MET	Integer / Null	0 to OPP_OBSERVABLE or Null	ClinRO-T items 1–6 only; rater-determined.
[DOM]7_EPISODE_COUNT	Integer / Null	≥0 or Null	Item 7 only; source value for derivation

Variable	Type	Range / levels	Notes
			of the raw episode band.
[DOM][ITEM]_RAW, items 1–6	Categorical	{1, 2, 3, 4, 5, NO, S-NA, TECH}	Original or derived ordinal category or applicable non-numeric code.
[DOM]7_RAW	Categorical	{1, 2, 3, 4, 5, NO, TECH}	Derived episode-count band or applicable non-numeric code; S-NA is unavailable.
[DOM][ITEM]_SCORE	Integer / Null	1–5 or Null	Items 1–6 retain a numeric RAW value; item 7 is reverse-scored. Null for applicable non-numeric codes.
[DOM]_MEAN	Float / Null	1–5 or Null	Null if <5/7 valid numeric items.
[DOM]_ITEMS_USED	Structured string/list or Null	Item identifiers	Identifies the item scores contributing to the domain mean.
N_AVAILABLE_DOMAINS	Integer	0–5	Count of available domain means.
DOMAIN_VALID_ITEMS_N	Integer	0–7	Count of numeric item scores available within a domain.
QC_*	Binary	{0,1}	1 = flag; 0 = pass (quality-control flags below).

7 Governance, traceability, and version control

- All transformations—band derivation, reverse-scoring, aggregation and flagging—are deterministic and documented in the electronic-form logic and data-transformation documentation.
- Before form freeze, changes to definitions, thresholds or variable names are version-controlled through a change log recording date, time, author and rationale. After form freeze, a substantive change requires a documented protocol amendment, generates a new schema or form version and applies prospectively.
- DCRs, response-process QC reviews and procedural resolutions are append-only; original entries are never overwritten and no locked rating is altered or merged.

8 Notes on alignment with the pre-implementation-phase

- This specification does not implement imputation or inferential modeling; it classifies and flags data for transparent handling during collection.
- Phase 1 analyses comprise inter-rater agreement, measurement-process indicators and exploratory descriptive cross-context correspondence. Domain-structure evaluation, responsiveness and measurement error are deferred to a subsequent purpose-designed validation study. All analyses use this schema without altering source records or audit trails.

Supplementary Material S7

Codebook and scoring logic

1 Purpose and scope

This codebook specifies the deterministic scoring procedures for the dual-informant COA system, covering:

- derivation of item-level ordinal bands;
- reverse-scoring of item 7;
- domain-level aggregation under the 5-of-7 domain-score availability rule;
- derivation of N_AVAILABLE_DOMAINS;
- standardized handling of NO, S-NA and TECH;
- integration with quality-control monitoring and the prespecified response-process review.

Phase 1 analyses comprise inter-rater agreement, measurement-process indicators and exploratory descriptive cross-context correspondence. The elements deferred to a subsequent purpose-designed validation study are specified in S10.

2 Dataset architecture and variable map

Scoring operates on a structured longitudinal dataset in which one record represents one locked COA submission. ClinRO-T produces separate rater-specific records for Psychologists A and B. Variable definitions and allowed values are specified in S6.

Primary identifiers and timing

- P_ID, S_ID, COA_TYPE and RATER_ID; RECRUITMENT_SOURCE; and, for ObsRO-P, INF_ID, INF_RELATION_CLASS and INFORMANT_CHANGE
- SESSION_END_TS, FORM_OPEN_TS, SUB_TS, SUBMISSION_LATENCY_HOURS and COMPLETION_DURATION_MIN
- FORM_VERSION and FORM_DATE

Contextual and traceability metadata—including SEGMENT_ID, SEGMENT_POSITION, ORCHESTRAL_SECTION, ASSIGNED_PART_ID, RECORDING_CONFIG_ID, SCORE_MAP_VERSION and REALIZED_EVENT_LOG_VERSION—are retained as specified in S6 but are not transformed by the scoring algorithm.

Item-level fields

- [DOM][ITEM]_RAW — ordinal raw band or applicable non-numeric code
- [DOM][ITEM]_SCORE — final item score from 1 to 5 or Null
- [DOM][ITEM]_OPP_REALIZED, [DOM][ITEM]_OPP_OBSERVABLE and [DOM][ITEM]_MET — ClinRO-T items 1–6 only
- [DOM]7_EPISODE_COUNT — item 7 only

Domain prefixes

- COG (Cognitive–Executive),
- SOC (Socio–Communicative),
- EMO (Emotional–Motivational Regulation),
- MOT (Motor–Sensorimotor),
- SYN (Synchronization)

Aggregated indicators

- [DOM]_MEAN and [DOM]_ITEMS_USED for each domain; N_AVAILABLE_DOMAINS (0–5), defined as the number of available domain means
- Quality-control flags specified in S6, including QC_DOMAIN_UNAVAILABLE, QC_WINDOW_NONCOMPLIANT and QC_TECH_FLAG

3 Item-level routing and derivation

3.1 ClinRO-T items 1–6

- The platform applies the following sequence:
- If a technical failure prevents scoring, set [DOM][ITEM]_RAW = TECH and [DOM][ITEM]_SCORE = Null.
- If [DOM][ITEM]_OPP_REALIZED = 0, set [DOM][ITEM]_RAW = S-NA and [DOM][ITEM]_SCORE = Null.
- If [DOM][ITEM]_OPP_REALIZED >0 but [DOM][ITEM]_OPP_OBSERVABLE = 0, set [DOM][ITEM]_RAW = NO and [DOM][ITEM]_SCORE = Null.
- If [DOM][ITEM]_OPP_OBSERVABLE >0, verify: 0 ≤ [DOM][ITEM]_MET ≤ [DOM][ITEM]_OPP_OBSERVABLE ≤ [DOM][ITEM]_OPP_REALIZED.
- Calculate: p = [DOM][ITEM]_MET / [DOM][ITEM]_OPP_OBSERVABLE
- Derive [DOM][ITEM]_RAW from p:

Proportion p	RAW band
p ≥0.90	5
0.70 ≤ p <0.90	4
0.40 ≤ p <0.70	3
0.10 ≤ p <0.40	2
0 ≤ p <0.10	1

- For items 1–6, set [DOM][ITEM]_SCORE = [DOM][ITEM]_RAW.

The realized-opportunity count is supplied by the frozen realized-event log. The rater enters only OPP_OBSERVABLE and MET. All three counts and the derived band are retained.

3.2 ObsRO-P items 1–6

The caregiver does not enter opportunity counts. The platform accepts one of the following routes:

- a numeric frequency category from 1 to 5 when at least one relevant situation occurred and behavior was directly observed;
- S-NA when no relevant situation occurred;
- NO when at least one relevant situation occurred but the caregiver did not directly observe the participant's behavior;
- TECH when a technical failure prevents form completion or submission.

For a numeric response, [DOM][ITEM]_SCORE = [DOM][ITEM]_RAW. For NO, S-NA or TECH, [DOM][ITEM]_SCORE = Null. No opportunity denominator is recorded.

3.3 Item 7 in both channels

Item 7 is derived from episode count and does not use an opportunity denominator. S-NA is not an allowed response.
When the reporting window was adequately observed, the rater or caregiver enters [DOM]7_EPISODE_COUNT as an integer ≥0. The platform derives the raw episode band according to channel.

ClinRO-T: 20-minute segment

EPISODE_COUNT	RAW band
0	1
1	2
2	3
3	4
≥4	5

ObsRO-P: 48-hour interval

EPISODE_COUNT	RAW band
0	1
1–2	2
3–6	3
7–12	4
≥13	5

If observation was insufficient to determine whether episodes occurred, set [DOM]7_RAW = NO and [DOM]7_EPISODE_COUNT = Null. If a technical failure prevents entry or submission, set [DOM]7_RAW = TECH and [DOM]7_EPISODE_COUNT = Null.
Non-numeric responses are not reverse-scored and produce [DOM]7_SCORE = Null.

4 Reverse-scoring of item 7

Reverse-scoring ensures consistent directionality across all items.

- Applicability: only item 7 in each domain (A7/B7/C7/D7/E7) and only if [DOM]7_RAW ∈ {1–5}.
- Transformation:

[DOM]7_SCORE = 6 − [DOM]7_RAW

- Items 1–6: [DOM][1–6]_SCORE = [DOM][1–6]_RAW; no reverse-scoring is applied.
- For item 7, NO or TECH produces [DOM]7_SCORE = Null. S-NA is not available.

Interpretive note (pre-implementation): a higher Item_Score reflects greater expression of the target referent within the domain's behavioral scope. This is an observational convention, not a clinical claim.

5 Domain-score availability and aggregation

Compute a domain mean only when at least 5 of 7 items in that domain have numeric scores from 1 to 5.

- When at least 5 items are numeric:

[DOM]_MEAN = sum of available [DOM][ITEM]_SCORE values / number of available numeric item scores
Record the identities of the contributing items in [DOM]_ITEMS_USED.

- When fewer than 5 items are numeric:

[DOM]_MEAN = Null
QC_DOMAIN_UNAVAILABLE = 1
DOMAIN_VALID_ITEMS_N retains the number of numeric item scores available.

NO, S-NA and TECH do not contribute numeric values to the mean. No imputation is applied.

6 Number of available domains

N_AVAILABLE_DOMAINS is the number of domain means satisfying the 5-of-7 domain-score availability rule. It is derived for every record and ranges from 0 to 5. N_AVAILABLE_DOMAINS is a data-availability indicator only. It has no clinical or functional-score interpretation, and no arithmetic composite across the five domains is computed in phase 1. A value of 0 is a valid derived value and is not itself an uncoded missing value.

7 Quality-control hooks and timing safeguards

Scoring is integrated with the quality-control monitoring specified in S6.

Timing

- QC_WINDOW_NONCOMPLIANT = 1 when SUBMISSION_LATENCY_HOURS >24 hours for ClinRO-T or >48 hours for ObsRO-P.
- Window-noncompliant records are retained in the all-submitted dataset, excluded from the primary window-compliant analysis set and retained for sensitivity summaries.
- COMPLETION_DURATION_MIN is summarized descriptively and does not independently determine record validity.

Pattern anomalies

- QC_PATTERN_ANOMALY = 1 when the prespecified data-processing rules identify an invariant, straight-line or other non-differentiated response pattern. PATTERN_TYPE records the platform-derived classification of the pattern.
- The flag initiates descriptive response-process review and does not independently invalidate the record.

Technical interruptions

- QC_TECH_FLAG = 1 when a TECH response is present; ITEM_TECH_FLAG registers the affected item.
- The affected item is excluded from aggregation but retained for feasibility, traceability and technical review.

Completeness and domain-score availability

- QC_INCOMPLETE = 1 when an item lacks both a numeric response and an applicable NO, S-NA or TECH code; ITEM_COMPLETE_FLAG registers the affected item.
- QC_DOMAIN_UNAVAILABLE = 1 when fewer than 5 of 7 item scores are numeric within a domain. DOMAIN_VALID_ITEMS_N retains the corresponding count.

Prespecified caregiver response-process review

- QC_REVIEW_TRIGGER = 1 when an ObsRO-P submission is randomly selected into the prespecified 20% response-process review subset. Selection does not indicate an anomalous response.

All quality-control flags are non-destructive and accompany the source record. They support the data-clarification workflow described in S11.

8 Worked examples (scoring and aggregation)

Item-7 derivation and reverse-scoring: ClinRO-T, Domain C item 7

Example 1: one observed episode

- EMO7_EPISODE_COUNT = 1
- EMO7_RAW = 2
- EMO7_SCORE = 6 − 2 = 4

Example 2: no observed episodes during an adequately observed segment

- EMO7_EPISODE_COUNT = 0
- EMO7_RAW = 1
- EMO7_SCORE = 6 − 1 = 5

Example 3: technical failure

- EMO7_EPISODE_COUNT = Null
- EMO7_RAW = TECH
- EMO7_SCORE = Null
- QC_TECH_FLAG = 1

Domain mean with NO/S-NA exclusions (ObsRO-P, SOC)

- Valid numeric items: 5 of 7 (e.g., SOC1_SCORE=3, SOC2_SCORE=4, SOC3_SCORE=3, SOC4_SCORE=5, SOC6_SCORE=4)
- Non-numeric: SOC5_RAW = S-NA, SOC7_RAW = NO
- SOC_MEAN = mean(3,4,3,5,4) = 3.8 (the 5-of-7 domain-score availability rule is met).
- SOC_ITEMS_USED = {SOC1, SOC2, SOC3, SOC4, SOC6}.

Domain-score availability example

- Available domain means: COG_MEAN = 3.6, SOC_MEAN = 3.8 and EMO_MEAN = 3.2.
- Unavailable domain means: MOT_MEAN = Null and SYN_MEAN = Null.
- N_AVAILABLE_DOMAINS = 3.

9 Data clarification request interface

When a quality-control flag requires clarification:

- For ClinRO-T, no data clarification request is issued and no paired scoring pattern is reviewed until both rater-specific records for the affected participant-session observation have been locked (S3; S11).
- The OPBG Clinical Research Coordinator issues a data clarification request (DCR) identifying the variable, record and anomaly.
- The relevant rater or caregiver may provide procedural context. An externally identifiable administrative or technical error may be documented through an appended clarification.
- The procedural resolution is appended to the audit trail; no locked rating is altered.

Original entries are never overwritten; clarifications remain append-only.

10 Governance and version control

- All scoring transformations are deterministic and documented in the electronic-form logic and data-transformation documentation.
- Before form freeze, changes to variable names, thresholds or scoring rules are version-controlled through a change log recording date, time, author and rationale. After form freeze, a substantive change requires a documented protocol amendment, generates a new version and applies prospectively.

No post-hoc rule modification is applied to already finalized records; historical logic remains reproducible.

Supplementary Material S8

Interface specifications and temporal windows

1 Purpose and scope

This document specifies the user interface (UI) and time-handling configuration for the dual-informant COA system:

- ClinRO-T (clinician-reported) and ObsRO-P (caregiver-reported),
- KoBoToolbox (ODK-based) delivery with Enketo web forms,
- standardized timing, routing, validation, and metadata capture,
- security, privacy, and accessibility guardrails.

The scope is limited to pre-implementation operational details (no data analysis or psychometric testing).

2 Platform and delivery model

- Platform: KoBoToolbox (ODK-based), rendered via Enketo responsive web forms.
- Device coverage: smartphone, tablet, laptop/desktop (modern browsers).
- Distribution: secure, role-segregated links; independent channels for ClinRO-T and ObsRO-P.
- Submission control: the platform will be configured to apply post-submission locking.
- Auditability: server-side timestamping and logging are prespecified; correct operation will be verified through user-acceptance testing (UAT).

3 Temporal windows and timing safeguards

Reporting windows (fixed, prespecified)

- ClinRO-T: ≤ 24 hours after session end.
- ObsRO-P: ≤ 48 hours after session end.

Completion-duration monitoring

- Form-open and submit timestamps are retained to describe form-entry duration. No fixed minimum or maximum duration is treated as evidence of record validity.
- Completion duration is interpreted only alongside completeness, technical logs, and documented deviations.

Time anchoring and latency computation

- The platform records SUB_TS (server-side finalization time).
- SUBMISSION_LATENCY_HOURS = (SUB_TS − session_end_timestamp)/3600; used to enforce window compliance.

Timing-related QC hooks (non-destructive flags)

- QC_WINDOW_NONCOMPLIANT = 1 if submission exceeds the window (ClinRO-T >24 h; ObsRO-P >48 h).
- Completion duration is retained descriptively and does not independently trigger exclusion or a data-query request.
  (Flags accompany the record; they do not delete or overwrite original entries.)

4 Form structure and routing

Domain and item layout

- Five domains (COG, SOC, EMO, MOT, SYN), 7 items/domain (items 1–6 positive; item 7 negative).
- ClinRO-T and ObsRO-P use paired, context-adapted behavioral referents. Pairing denotes intended functional correspondence and implies no metric equivalence; in Domain E the daily-life items are functional analogues of ensemble synchronization rather than identical referents.

Routing and response pathways

- Primary response: ordinal category 1–5. For ClinRO-T items 1–6 the form displays the locked realized-opportunity count supplied by the frozen realized-event log. The rater enters only the number of realized opportunities that were observable and the number on which the criterion was met; the ordinal category is derived server-side from MET / OPP_OBSERVABLE against the prespecified proportion bands (S5, S7), is displayed for confirmation and is not free-selected. For ObsRO-P items 1–6 the frequency category is selected directly. For item 7 in both channels the reporter enters the number of qualifying episodes observed within the applicable window, and the platform derives the raw episode band from the channel-specific boundaries specified in S5 and S7; the derived band is displayed for confirmation and is not free-selected.
- Non-observability routes (item-specific availability): for items 1–6 all three routes are available; for item 7, S-NA is disabled because the denominator is the observation interval rather than discrete opportunities, so zero qualifying episodes is entered as the valid floor category and NO is used only when the window was insufficiently observable. Routes available:
    - NO (Not Observed),
    - S-NA (Structural Not Applicable),
    - TECH (Technical interruption).
- Routing rules are configured so that choosing NO/S-NA/TECH bypasses numeric entry, preventing forced responses; correct operation will be verified through user acceptance testing.

Required/optional logic

- Items 1–6 require a selection among 1–5, NO, S-NA and TECH. Item 7 instead requires a non-negative episode count when the window is adequately observed, or NO/TECH when applicable; S-NA is unavailable for item 7.
- If no selection is made, a gentle validation prompt appears.
- No additional free text is required; optional comments can be disabled to minimize burden and analytic ambiguity (pre-implementation).

5 Validation, constraints, and error messaging

- Client-side prompts for missing choices.
- Mutual exclusivity: numeric selection and NO/S-NA/TECH cannot co-occur on the same item.
- Consistent scales: channel-specific ordinal response anchors are displayed consistently across devices and sessions.
- Minimal friction: validation text is short, neutral, and non-directive.

6 Non-observability taxonomy (UI behavior)

- NO → marks that an opportunity for the behavior arose within the window but the participant could not be observed at that moment. Non-occurrence when the opportunity arose and the participant was observable is a rating of 1, not NO (main text §3.1.10; S5).

- S-NA → marks the absence of any opportunity for the behavior within the window.
- TECH → marks technical interruption (e.g., network loss, device failure).
- UI effect:
  - For NO/S-NA/TECH, the numeric fields are disabled or bypassed.
  - A short informational note may appear ("Recorded as non-observable; this item will not contribute to domain scoring").
  - TECH also displays a discreet nudge to attempt a retry if still within window.

7 Metadata capture (server-side)

The form captures, at minimum:

- SUB_TS (server-finalized timestamp),
- COMPLETION_DURATION_MIN (minutes open→submit),
- device/browser fingerprints where available (non-identifying),
- submission status/latency used for window checks,
- routing footprints when needed for traceability or QC review (e.g., whether NO/S-NA/TECH was presented/selected).

All metadata are append-only and governed by the pseudonymization model (see S11).

8 Scoring-relevant interface behavior

- Items 1–6 produce numeric values 1–5.
- Item 7 is presented as an episode-count entry rather than as a direct category selection. It is negatively valenced and is reverse-scored in processing, not on screen.
- NO/S-NA/TECH selections set [DOM][ITEM]_SCORE = Null.
- Domain means are computed only if ≥5/7 valid numeric items (5-of-7 rule).
- TECH selections are retained in the dataset, excluded from aggregation, and QC-flagged (QC_TECH_FLAG = 1).

9 Security, privacy, and access control

- Encrypted transmission, role-segregated links, and post-submission lock.
- No direct identifiers are collected in the analytical dataset; P_ID is pseudonymous and S_ID denotes the scored session (1–20).
- The re-identification file and any direct identifiers are controlled by the Data Controller (S11).
- Audit trail is preserved for all finalized entries and DCR resolutions.

10 Accessibility and usability

- Responsive layout (mobile-first), legible contrast, concise labels.
- Minimal scrolling where possible; domain sections separated visually.
- Avoidance of dense instructional text; plain-language micro-copy near controls.
- No reliance on color only for meaning.

- No completion-time limit is displayed; secure re-entry remains possible within the applicable temporal boundary.

11 Error handling and TECH registration

- Loss of connectivity or browser crash: if the session cannot be resumed reliably, the reporter can reopen the secure link and submit within the window.
- If a device/platform error prevents completion of an item, the reporter can select TECH for that item.
- TECH triggers QC_TECH_FLAG = 1; the record remains in the dataset (excluded from aggregation).

12 Versioning and change control

- Each live form carries a FORM_VERSION string and a FORM_DATE.
- Any change (text, routing, options, layout) is recorded in a version-controlled changelog (append-only) and communicated before the next collection cycle.
- Historical forms remain reproducible (linked to platform snapshots and ETL notes).

13 Planned UAT (pre-implementation)

UAT has not yet been performed. Before phase 1 implementation, it will verify:

- routing logic for NO/S-NA/TECH,
- timing capture (SUB_TS, COMPLETION_DURATION_MIN),
- window calculations (SUBMISSION_LATENCY_HOURS),
- display integrity across device classes,
- post-submission lock and audit logging.

UAT will not collect behavioral data; it will assess technical implementability only. No technical performance is reported in the present manuscript.

Supplementary Material S9

External anchoring and Vineland-3 mapping

1 Purpose and scope

This document defines how the Vineland Adaptive Behavior Scales, Third Edition (Vineland-3) is used as an external interpretive anchor for the dual-informant COA system (ClinRO-T / ObsRO-P). It clarifies conceptual non-equivalence (extended-timeframe adaptive functioning vs. bounded-interval observation), administration and timing, data-handling safeguards, and the high-level mapping strategy used to contextualize COA indicators without implying metric identity, calibration, or psychometric interchangeability.

2 Conceptual positioning

COA (bounded-interval)

Bounded-interval, contextually proximal observations with a ≤24-hour ClinRO-T coding deadline and a 48-hour ObsRO-P post-session observation/submission interval. COA indicators characterize observable behavioral expression within the specified session and daily-life intervals.

Vineland-3 (trait-like)

Semi-structured clinician-administered interview indexing adaptive functioning across extended timeframes (habits/skills, typical behavior), producing norm-referenced standardized scores.

Implication

The relationship is partial and conceptual, seeking interpretive convergence where functional domains overlap (e.g., communication, daily living, socialization, motor aspects), while preserving the non-equivalence of temporal scope, score metrics, and intended use.

3 Administration and timing

Targets and schedule

Vineland-3 is administered at T0 (baseline) and T1 (at completion of the 20-week phase 1 observation period, after approximately 20 OMT sessions) to describe trait-like profiles and contextualize inter-individual heterogeneity in the anticipated approximately 14–32-year recruitment range.

Role segregation

Psychologist A conducts both T0 and T1 interviews in a semi-structured format to ensure procedural consistency and informed probing. Psychologist B has no access to Vineland-3 interview content or results before completion and locking of all independent ClinRO-T ratings.

COA channel independence

ClinRO-T and ObsRO-P are collected via segregated digital channels with server-side timestamping and post-submission locking; Vineland-3 is not used to guide COA scoring, routing, or feedback during data collection.

4 Conceptual mapping strategy

Objective

Provide a contextual lens for interpreting COA patterns—for example, to examine whether participants with specific Vineland-3 profiles (communication, socialization, daily living, motor) show differences in domain-level summaries across the five scheduled observations (COG, SOC, EMO, MOT, SYN), without presuming score equivalence or transferability.

Mapping principles

- Domain-to-domain alignment (conceptual only):
    - COA–SOC ↔ Vineland-3 Socialization (conceptual overlap in reciprocal interaction and participation).
    - COA–COG / COA–EMO ↔ aspects of Communication/Socialization that require regulation, engagement, and flexible behavior (interpretive, not causal).
    - COA–MOT / COA–SYN ↔ Motor and participation-relevant routines (e.g., coordination, timing demands), strictly at the level of contextual interpretation.
- Non-equivalence disclaimer: COA domain summaries and Vineland-3 standard scores index distinct constructs at different temporal scales; no direct metric mapping, no back-calibration, no score rescaling.
- Analysis readiness (future phase 1): If exploratory convergence is assessed, it will use correlational or grouping approaches appropriate for contrasting T0/T1 standardized adaptive-functioning Vineland-3 scores with the participant-level COA summary defined in S10, namely the median of the participant's five domain means for each domain, computed only over occasions on which that domain mean satisfied the ≥5-of-7 rule. Analyses use Psychologist B's locked ClinRO-T records and carry no assumption of bidirectional inference.

5 Data-handling, independence, and governance

- Pseudonymization: analytical datasets store the pseudonymous P_ID and session identifier S_ID only; the re-identification file linking P_ID to personal identity is held separately by the Data Controller (see S11).
- Audit trail: All submissions (COA) and interviews (Vineland-3 scoring outputs) are logged; DCRs and their procedural resolutions are append-only.
- Informant independence: Psychologist B does not access ObsRO-P; caregivers do not see ClinRO-T outputs; Vineland-3 interview content is not exposed to informants beyond standard consent and interview procedures.
- No feedback loops: Vineland-3 is not used to coach COA responses, alter anchors, or modify COA workflows or thresholds in the pre-implementation phase.

6 High-level anchoring use-cases

These use-cases are prospective and non-inferential in the pre-implementation phase; any exploration occurs during the observational study:

- Descriptive stratification: Group repeated-observation COA summaries by Vineland-3 profiles (e.g., quartiles in communication or socialization) to examine qualitative differences in domain completeness, non-observability patterns (NO/S-NA/TECH), or distributions across the five scheduled occasions.
- Feasibility-context checks: Examine descriptively the relationship between Vineland-3 adaptive scores and the availability of valid COA item responses (e.g., frequencies of NO/S-NA and satisfaction of the 5-of-7 domain-score availability rule), without prespecifying a direction of association; this is a feasibility-anchored analysis, not a psychometric test.

- Pattern description (non-metric): Use Vineland-3 only to contextualize descriptive variation across the five scheduled COA observations, without fitting individual trajectories, re-scaling scores, or imputing across instruments.

7 Constraints and prohibited practices

- No metric alignment: Do not re-express COA domain summaries as Vineland-3 standard scores or vice versa.
- No causal inferences: Do not interpret trait–state associations as mechanistic or directional.
- No forced convergence: Vineland-3 results do not modify COA ratings, response anchors, scoring rules, the 5-of-7 domain-score availability rule or missingness handling. COA scoring remains based on item-level scoring, the 5-of-7 domain-score availability rule, and N_AVAILABLE_DOMAINS as a 0–5 data-availability indicator; no cross-domain COA total score is computed (see S5–S7).
- No psychometric property of the COA is inferred from Vineland-3 stratification or correspondence in phase 1. These analyses are exploratory and contextual; they do not establish reliability, responsiveness, criterion validity, metric equivalence or score interchangeability.

8 Operational workflow

- At T0/T1: Conduct Vineland-3 interviews (Psychologist A), finalize scoring outputs, and store as pseudonymous records.
- During COA collection: No changes to COA windows, routes, or anchors based on Vineland-3.
- QC and DCR: If timing or pattern anomalies occur in COA, these are handled via standard QC flags and DCR—never via Vineland-3-based overrides.
- Version control: Any changes in interview procedures, scoring documentation, or export formats are recorded in the governance changelog (see S11/S12).

Supplementary Material S10

Statistical framework

1 Purpose and scope

This document specifies the planned analytical framework for the phase 1 observational implementation of the dual-informant COA system. The focus is on:

- descriptive feasibility (timing, adherence, data availability),
- descriptive measurement-process behavior (e.g., non-observability patterns, domain-score availability rule),

- prespecified inter-rater agreement for ClinRO-T and exploratory descriptive cross-context correspondence between the two channels.

Phase 1 estimates inter-rater agreement and measurement-process indicators using methods appropriate to ordinal observer ratings and clustered repeated observations; domain-structure evaluation, responsiveness and measurement error are deferred to a subsequent purpose-designed validation study; no such analyses are undertaken here.

Observational base

All analyses operate on the prespecified balanced panel of the main text §3.1.4: N = 20 participants, m = 5 scored occasions per participant, S = 20 scored sessions, O = N·m = 100 participant-session observation units and, with independent double-coding, 200 ClinRO-T rating records.

2 Analysis populations and records

Analysis sets

- All-submitted set: all finalized COA entries (ClinRO-T and ObsRO-P) retained with audit metadata.
- Primary analytic set (window-compliant): excludes entries with QC_WINDOW_NONCOMPLIANT = 1. Window-violating entries remain available for sensitivity summaries (non-primary).
- Complete paired ClinRO-T set: all scheduled participant–session observations independently coded by Psychologists A and B (target n = 100); observations lacking two locked ratings are documented as protocol deviations and excluded from the primary inter-rater agreement analysis.

Record retention and flags

All records are append-only and never overwritten. QC flags (e.g., QC_TECH_FLAG and QC_PATTERN_ANOMALY) are non-destructive and accompany records across analyses (see S6/S7).

3 Variables and pre-processing

Item handling

- Valid numeric entries: 1–5.
- Non-observability: NO, S-NA, TECH → excluded from aggregation; TECH is retained and QC-flagged. For items 1–6, the recorded and observable opportunity counts are retained and their distribution is summarized, so that scores computed on few observable opportunities are distinguishable from scores computed on the full set.
- Negative-valence items (A7/B7/C7/D7/E7): reverse-scored prior to aggregation when Raw is numeric from 1 to 5, using [DOM]7_SCORE = 6 - [DOM]7_RAW (see S5 and S7).

Domain means and domain coverage

- 5-of-7 rule: compute [DOM]_MEAN only when ≥5/7 items carry valid numeric scores (1–5). Otherwise, set to Null and raise QC_DOMAIN_UNAVAILABLE = 1.
- N_AVAILABLE_DOMAINS: count of domain means meeting the 5-of-7 rule (0–5); no cross-domain composite is computed (see S6/S7).

Timing metrics

- SUBMISSION_LATENCY_HOURS: hours from session end to server-finalization; verifies the ≤24 h ClinRO-T coding deadline and submission by the end of the 48 h ObsRO-P interval.
- COMPLETION_DURATION_MIN: minutes from form open to submit; summarized descriptively as a feasibility measure.

4 Feasibility and data-quality summaries (primary descriptive outputs)

Adherence and throughput

- Per-participant and overall submission counts and completion rates by week and informant.
- Distribution of SUBMISSION_LATENCY_HOURS relative to the ClinRO-T deadline and ObsRO-P interval end. Window-violating entries are summarized separately and by latency band (0–6 h, 6–24 h and more than 24 h beyond the applicable boundary), so that marginal ObsRO-P submissions, in which the observation interval and the submission deadline coincide at 48 h, are distinguishable from substantive non-compliance. Retention in the all-submitted set and exclusion from the primary analytic set are unchanged.

Response-process and non-observability

- Frequencies of NO / S-NA / TECH by domain, informant and scheduled segment phase over time; observable-opportunity yield is summarized as the complement of structural non-observability.
- Domain-score availability rate (at least 5 of 7 items) and reasons for failure (e.g., excess NO/S-NA).
- QC flags (e.g., QC_PATTERN_ANOMALY and QC_TECH_FLAG) as counts and percentages; pattern anomalies are reported using the platform/ETL classification scheme defined in S6.

Score availability and distributions

- Per-domain availability (proportion of non-Null [DOM]_MEAN) and its temporal evolution.
- Descriptive distributions (median, IQR) of [DOM]_MEAN by informant and week; and the distribution of N_AVAILABLE_DOMAINS as a data-availability indicator.

These summaries are designed to characterize feasibility and prespecified data-quality dimensions in the planned observational dataset, including structural integrity, completeness, consistency, temporal

compliance, and score availability, consistent with COA best-practice reporting and the need for structured pre-implementation checks.

5 Missing-data handling and sensitivity

Treatment policy

- No imputation is planned for phase 1.
- Non-observability codes (NO/S-NA/TECH) are treated as classified missingness and excluded from aggregation.
- TECH is retained for feasibility and traceability.

Sensitivity views

- Window-violating entries (QC_WINDOW_NONCOMPLIANT = 1) will be summarized separately to understand the extent and potential impact of temporal non-compliance.
- Completion-duration distributions will be summarized descriptively and will not be used alone to classify inattentive responding.

6 Planned measurement-process descriptives (phase 1)

The following are planned for phase 1; methods are declared now for transparency. No estimates are produced in the pre-implementation phase.

6.1 Inter-rater agreement (ClinRO-T; complete paired set)

Agreement analysis will distinguish ordinal item ratings from domain-level means. Item-level agreement will be summarized using quadratic-weighted Cohen's kappa, exact agreement, and agreement within one response category. Domain-level agreement will use a two-way random-effects, absolute-agreement, single-measure intraclass correlation coefficient (ICC(2)). Ninety-five percent confidence intervals for both coefficients will be obtained by participant-level cluster bootstrap so that the five repeated observations per participant are not treated as independent. No coefficient will be converted into a validation threshold. Concordance obtained on the simulated calibration material before the first scored session (S3) is computed with the same statistics and reported separately from the phase 1 estimates; it characterizes training adequacy and anchor discriminability and is not an instrument reliability estimate.
Agreement is reported at three levels: per item, per domain, and by observational-demand class (score-location, cue-response, temporal/synchronization, motor-sequence, spontaneous-event, and episode-count items). Class-level summaries locate any agreement shortfall by the kind of observation an item requires rather than by domain alone. A domain-level shortfall does not by itself warrant revision of the domain: it initiates the diagnostic review specified in the main text (§4.2), and only referent ambiguity surviving that review bears on the domain structure—the remaining sources (training, the perceptual demand of the items, the adequacy of the score map or realized-event log, and audio-video quality) being procedural or technical.

Quantities on which agreement is computed

Agreement on ClinRO-T items 1–6 is examined for the two rater-determined quantities, the observable-opportunity count and the criterion-met count, and for the ordinal category derived from

them. The realized-opportunity count is supplied by the common frozen event-log layer and is therefore not treated as an independently rated quantity: computing agreement on it would be inflated by construction. Agreement is additionally reported by denominator class (fewer than 4, 4–9, and 10 or more observable opportunities), because opportunity density varies with the instrumental part.
For each rater-determined count, exact agreement and the absolute rater difference will be reported; count-level summaries will also be stratified by denominator class.

6.2 Cross-context correspondence

Cross-context correspondence will be examined separately for Psychologist A's locked ClinRO-T records versus the matched ObsRO-P records and for Psychologist B's locked ClinRO-T records versus the same ObsRO-P records. For each domain, matched participant-week pairs will be displayed using paired distribution or scatter plots and summarized using Spearman rank correlation, with ninety-five percent descriptive uncertainty intervals obtained by the participant-level cluster-bootstrap procedure specified in the agreement-analysis part of this document (S10). Analyses are exploratory and are not pooled across clinician raters. Because the two channels differ in reporter, context and response process, these coefficients are not interpreted as inter-rater reliability, metric equivalence, criterion validity, or evidence of transfer. The two analyses are parallel and exploratory; they are not pooled or averaged, no composite ClinRO-T score is created across raters, and a difference between them indexes rater dependence of the clinician channel rather than a property of the daily-life channel. Repeated observations within participants are accounted for descriptively. Analyses acknowledge contextual and metric non-equivalence and are not intended to estimate agreement or establish transfer.

6.3 Descriptive within-person variation

Descriptive within-person variation summaries across the 20-week period (for example, median absolute within-person difference and run charts).
No idiographic state-space, latent-trajectory, or time-series model is planned because five observations per participant are insufficient for those analyses.

Prespecified sensitivity and composition analyses

Item composition of domain summaries: the identity of the contributing items is recorded with every domain summary and reported with it, and the proportion of summaries suppressed under the ≥5-of-7 rule is reported; domain means computed over different subsets are not compared directly without stating the composition. Domain C nested items: because C2, C6 and C7 index the same behavior class at increasing durations, Domain C is summarized both with the full item set and with a single member of that set retained; the retained item is selected prospectively during the pre-initiation content review and frozen before enrollment, with no phase 1 data informing the selection. Item 7: because its denominator is time rather than discrete opportunities, domain-level cross-context correspondence is reported with and without it; in addition, among records with all seven items numerically present, the seven-item domain mean and the items-1–6-only mean are compared as a paired within-record sensitivity, reported descriptively. External anchoring: exploratory correspondence with the Vineland-3 uses Psychologist B's locked ClinRO-T records, B being masked to the Vineland-3 results, and the participant-level value is the median of that participant's five domain means computed only over occasions satisfying the ≥5-of-7 rule; the corresponding analysis on Psychologist A's records is reported separately as subject to common-rater variance. Informant continuity: records carrying INFORMANT_CHANGE = 1 are described separately and no new

reporter is treated as continuous with the previous informant. Age band: domain summaries are additionally reported for the 14–17 and 18–32 strata, descriptively and without inferential comparison.

7 Multiple-comparison control and inference policy

No formal hypothesis testing or multiplicity control is planned in the pre-implementation phase. In phase 1, prespecified estimation of inter-rater agreement and of the associated descriptive uncertainty intervals will be reported as specified above and labeled clearly as post-collection analyses. Domain-structure evaluation, responsiveness and measurement error are not estimated in phase 1 and are deferred to a subsequent purpose-designed validation study.

8 Reporting, reproducibility, and version control

- Outputs: feasibility tables, QC summaries, non-observability profiles, availability of [DOM]_MEAN and the distribution of N_AVAILABLE_DOMAINS, and item-level and domain-level inter-rater agreement estimates, and cross-context plots.
- Reproducibility: all transformations and flags follow the deterministic rules specified in S6–S7; ETL and scoring logic are version-controlled (append-only changelog).
- Deviations: any deviation from this framework will be documented with date/time, rationale, and impact assessment in the analytical log.

Supplementary Material S11

Data-management plan

1 Purpose and scope

This Data-Management Plan (DMP) defines how COA data and related metadata are captured, protected, processed, stored, version-controlled, quality-controlled, and retired across the project lifecycle.
The DMP applies to both informant channels—ClinRO-T (clinician-reported) and ObsRO-P (caregiver-reported)—and covers:

- Pseudonymization and role-segregated access
- Secure data capture (KoBoToolbox/Enketo/ODK)
- Audit-trail and QC procedures informed by selected data-integrity attributes—contemporaneity, preservation of the original submitted record, traceability and auditability—in a non-GxP research context
- Retention and deletion schedules, backups, incident response, and change control
- Planned GDPR governance procedures, including legal-basis assessment, data-subject rights, and DPIA readiness

Phase 1 agreement and measurement-process analyses are specified in S10; domain-structure evaluation, responsiveness and measurement error are deferred to a subsequent purpose-designed validation study. This DMP does not prescribe analytic methods.

2 Roles and responsibilities

- Bambino Gesù Children's Hospital, IRCCS (OPBG; planned institutional Data Controller): under the governance arrangement to be formalized before study initiation, determines the purposes and means of processing. It holds the re-identification file and direct identifiers within its institutional environment, approves exports, authorizes retention or retirement, and oversees incident response.
- Principal Investigator (PI): ensures protocol adherence; approves data-access requests consistent with least-privilege principles.
- System Administrator (Platform/ETL): maintains KoBoToolbox/ODK configuration, user accounts, encryption parameters, server-side logging, backups, and restore procedures; enforces post-submission lock.
- Psychologist A (rating clinician): completes ClinRO-T; does not access ObsRO-P; has no access to the re-identification file.
- Psychologist B (Independent Secondary Rater): independently codes every scheduled ClinRO-T participant–session observation through a separate access path. The rater has no intervention or clinical-management role, does not attend live sessions, and cannot view Psychologist A's entries before both ratings are locked. The rater has no access to ObsRO-P, Vineland-3 results, clinical records, treatment objectives, or previous assessments. Masking to participant identity and program context is not claimed.
- OPBG Clinical Research Coordinator (CRC; to be appointed before study initiation): maintains delegation, training, deviation, and DCR logs and coordinates onboarding and version control. The CRC conducts the prespecified ObsRO-P response-process QC review, verifies that paired ClinRO-T ratings are locked before discrepancy review, and maintains the DMP changelog under PI oversight.
- OPBG Statistician: finalizes the Statistical Analysis Plan before data collection or database access; generates the random selection for the ObsRO-P QC review; receives only the pseudonymized frozen analytical dataset; documents analysis code and outputs; and does not issue DCRs or modify source ratings.
- Euterpe APS Operational Site Representative (to be designated before study initiation): verifies that required recording authorizations are documented for persons present, manages recording-field logistics and reports site-level deviations to the OPBG CRC; has no access to rating outputs.
- Data-Protection Officer (where applicable): advises on GDPR compliance, DPIA, and breach notification processes.

3 Data taxonomy and identifiers

All analytical data are pseudonymized; no direct personal identifiers appear in the working dataset.

- Primary keys (analytics):
  - P_ID (pseudonymous Participant Identifier)
  - S_ID (Session Identifier; 1–20)
- Informant channel: COA_TYPE ∈ {ClinRO-T, ObsRO-P}
- Timestamps and timing: SUB_TS (server-finalization), SUBMISSION_LATENCY_HOURS, COMPLETION_DURATION_MIN

- Items: [DOM][ITEM]_RAW ∈ {1..5, NO, S-NA, TECH}; [DOM][ITEM]_SCORE ∈ {1..5, Null}
- Aggregates: [DOM]_MEAN (Null if <5/7 valid items) and N_AVAILABLE_DOMAINS (0–5)
- QC flags: QC_WINDOW_NONCOMPLIANT, QC_PATTERN_ANOMALY, QC_TECH_FLAG, QC_DOMAIN_UNAVAILABLE, QC_INCOMPLETE, and QC_REVIEW_TRIGGER (see S6; S7)

The re-identification file linking P_ID to direct personal identifiers is physically and logically segregated and held solely by the Data Controller.

4 Data-flow overview

Data capture

ClinRO-T and ObsRO-P are submitted via independent secure links (KoBoToolbox/Enketo); client-side validation is configured to prevent forced numeric responses when NO/S-NA/TECH is selected (S8); correct operation will be verified through user acceptance testing.

Server processing

On submission, the platform will be configured to create a server-finalized, post-submission-locked record with SUB_TS and server-side audit metadata; correct operation of these controls will be verified through user-acceptance testing (UAT); routing footprints and metadata are logged server-side for audit.

QC attribution

ETL or form logic applies window- and pattern-based QC rules (e.g., QC_WINDOW_NONCOMPLIANT and QC_PATTERN_ANOMALY) and registers QC_TECH_FLAG on any TECH entry. Completion duration is retained descriptively.

Derivation layer

Item-level transformations occur deterministically (reverse-scoring only for item 7 with 1–5 raw; NO/S-NA/TECH excluded) to compute [DOM]_MEAN if the 5-of-7 rule is met; N_AVAILABLE_DOMAINS is derived on every record as the count of domain means satisfying the ≥5-of-7 rule and ranges from 0 to 5 (S5–S7).

Independent ClinRO-T coding and response-process QC

Psychologist B receives only the pseudonymized video segments, target identifiers and prespecified coding materials required for independent ClinRO-T rating. Psychologists A and B enter ratings through separate access paths; neither can view the other's entries before both records are locked. The OPBG CRC performs procedural QC and manages DCRs after locking; all clarifications and procedural resolutions are append-only, and neither original rating is overwritten (S3; main text §3.1.11).

4.1 Controlled export

Only the PI (or delegate) can approve exports of pseudonymized analytical datasets; direct identifiers and the re-identification file never leave the Data Controller's domain.

5 Security controls (capture storage export)

- Transport security: all submissions use encrypted transmission (HTTPS/TLS) from client to server.
- Storage security: platform/datastore encryption at rest (where configurable); logical separation of production vs. test/UAT environments.
- Access control: role-based access with least-privilege; two-factor authentication (2FA) enabled where available; session-timeout policies; no shared credentials.
- Audit logging: server-side logs capture authentication, submission events (SUB_TS), form version, and export operations; logs are protected from tampering and monitored periodically.
- Post-submission lock: once finalized, submissions cannot be edited; any subsequent clarifications are handled via DCR and appended to the record.

6 Pseudonymization and key management

- Key-separation model: the analytical dataset contains only the pseudonymous P_ID and session identifier S_ID. The re-identification file linking P_ID to direct personal identifiers is stored separately in a restricted repository controlled by the Data Controller; S_ID is not a re-identification key.
- Re-identification-key access: access to the re-identification file is limited to designated Data Controller personnel; every use (e.g., to coordinate consent withdrawal) is logged and justified.
- Re-identification policy: re-identification is prohibited unless explicitly authorized by the Data Controller for a legally permitted purpose (e.g., fulfilling data-subject rights).

7 QC integration and DCR governance

QC flags are non-destructive; they do not alter raw entries or derived scores.
Triggers:

- Timing (QC_WINDOW_NONCOMPLIANT)
- Pattern anomalies (QC_PATTERN_ANOMALY)
- Domain coverage (QC_DOMAIN_UNAVAILABLE)
- Completeness (QC_INCOMPLETE)
- TECH routing (QC_TECH_FLAG)

DCR workflow (append-only):

- Issue DCR (OPBG CRC / System Administrator) with date/time, record identifiers and description of the anomaly, only after any paired independent ratings have been locked.

- Rater clarification (procedural note, correction outside the locked record if appropriate).
- Procedural resolution (decision, rationale), appended to the audit trail.
- No raw record is ever overwritten; procedural resolutions remain traceable and attributable, and never replace or merge a locked rating.

8 Data-subject rights and legal basis (GDPR)

- Legal bases: the applicable data-processing legal basis and the related data-protection documentation will be finalized and documented by the institutional Data Controller and the relevant data-protection functions before study initiation. Study-specific consent, assent and research-video authorization procedures are specified separately and will be implemented only after ethics approval. Processing will be limited to the study purpose.
- Research-video authorization: study-specific written consent will separately cover recording at Euterpe APS, secure institutional storage, access by authorized research personnel and independent secondary rating. Consent will be obtained from each identifiable person with decision-making capacity; parental or legally authorized representative consent and developmentally appropriate assent or documented dissent procedures will apply where required. Identifiable professionals, musicians and volunteers will provide separate authorization. Any person lacking the required authorization will remain outside the recording field or be masked before research access. Ordinary APS media authorization will not be treated as research consent.
- Rights: access, rectification (via DCR notes—no direct overwrite of raw submissions), restriction of processing, withdrawal of consent (prospective), and erasure where legally applicable.
- Process: requests are handled by the Data Controller with PI support; feasibility is assessed case-by-case (e.g., complete erasure vs. continued retention in pseudonymized analytical form when legally permitted).
- Transparency: participants (and caregivers for ObsRO-P) receive plain-language information about data capture, windows, pseudonymization, and governance procedures.

9 Data retention and deletion

- Retention clock: starts at T1 (end of the observational period) or at final data-freeze, whichever is later.
- Active retention: the minimum period will be defined in the approved protocol, the opinion of the competent Territorial Ethics Committee and applicable institutional and legal requirements.
- Deletion: upon reaching end-of-life, pseudonymized analytical datasets and QC logs are securely deleted; the re-identification file is deleted last, after the analytical data are retired, to prevent accidental re-linkage.
- Interim purges: only test/UAT artifacts and failed uploads are purged routinely; production submissions are preserved until data-freeze and final retention review.

10 Backups and disaster recovery

- Backups: regular, encrypted backups of production data and logs; retention of backup copies consistent with institutional policy.
- Restore tests: periodic verification (e.g., quarterly) of restore procedures for integrity and completeness.
- Separation of duties: backup administration is segregated from analytical users, a configuration intended to limit privilege escalation.

11 Incident response and breach handling

- Detection and triage: continuous log monitoring for anomalies; clear escalation paths to the Data Controller and PI.
- Containment: prompt revocation of compromised credentials; session invalidation; platform hardening steps.
- Assessment: scope, data at risk, likelihood of identification, and potential harm.
- Notification: if required by law or by the competent Territorial Ethics Committee, data subjects and supervisory authorities are notified within statutory timelines; the Committee is informed as per protocol.
- Post-incident actions: root-cause analysis; remediation (technical and procedural); DMP update with lessons learned.

12 Cross-border transfer and vendor management

- Hosting locality: server region and data-residency are documented; if cross-border transfers occur, appropriate contractual and technical safeguards (e.g., SCCs) are applied.
- Vendor due diligence: platform providers (KoBoToolbox/ODK) are reviewed for security posture, uptime, data-processing terms, and incident-response commitments.
- Change management: any vendor or hosting changes trigger risk re-assessment, DMP updates, and communication to study stakeholders.

13 Validation, UAT, and configuration control

- UAT scope (pre-implementation): routing (NO/S-NA/TECH), timing capture, submission lock, audit logging, and the server-side derivation of the ClinRO-T ordinal category from the recorded counts; no behavioral data will be collected during UAT (see S8).
- Configuration records: every change to form text, routing, or validation is versioned (FORM_VERSION/FORM_DATE), with an append-only changelog.
- Freeze policy: before phase 1 data collection, a form freeze is performed; subsequent changes require PI and Data Controller approval and a new version identifier.

14 Training and awareness

- Raters and caregivers: receive procedural training/onboarding (S3–S4) emphasizing independent reporting, windows, and non-observability routes.

- System administrators and authorized QC personnel: receive platform-security, logging, backup and DCR training appropriate to their assigned access; understand least-privilege and segregation-of-duties principles.

15 Risk assessment and DPIA readiness

- DPIA triggers: introduction of new high-risk processing (e.g., novel linkages, new identifiers, cross-border hosting changes) prompts a DPIA or DPIA update.
- Mitigations: pseudonymization; encryption in transit/at rest; strict role segregation; append-only audit trail; non-destructive QC flags; incident-response plan; clear retention/deletion schedules.

16 Reproducibility, traceability, and documentation

- Deterministic transformations: reverse-scoring and aggregation rules are documented in S5–S7; all derived indicators are reproducible.
- Traceability: QC flags, DCRs, procedural resolutions and exports are logged with date/time, user, and rationale.
- Documentation set: protocol, DMP (S11), Codebook/Dictionary (S6–S7), UI/temporal windows (S8), anchoring (S9), and Statistical Framework (S10) remain synchronized and version-controlled.

Supplementary Material S12

Instrument development record and protocol traceability matrix

1 Scope and use of this document

This document has two parts. Part I is a development record for the dual-informant clinical outcome assessment system comprising ClinRO-T and ObsRO-P: it documents the derivation of the current item set and the content-development procedures that precede the phase 1 form freeze. Part II is a traceability matrix that assigns a status to each specification element and locates it in the main text and in Supplementary Materials S1–S12.

Three stages are distinguished throughout. The first comprises elements prespecified at the present pre-implementation stage. The second comprises elements evaluated in the phase 1 observational study, namely inter-rater agreement and measurement-process evidence. The third comprises elements deferred to a subsequent purpose-designed validation study: domain-structure evaluation, responsiveness and measurement error.

The matrix follows the structure of the study specification itself and not that of an intervention protocol. The inclusive orchestral programme functions as the observation context and is not the object of evaluation here.

1.1 Status tags

- Specified — documented in the main text and Supplementary Materials at the location indicated.

- Not applicable — outside the design by construction at the pre-implementation stage.
- Planned (pre-initiation) — declared for the content-development stage preceding the form freeze.
- Planned (phase 1) — declared for the phase 1 observational study.
- Deferred (validation study) — reserved for the subsequent purpose-designed validation study.

No empirical analysis is reported in this document: the matrix records specification, status and location only.

1.2 Notation

Domains are designated by the acronyms COG, SOC, EMO, MOT and SYN and, in the item codes, by the letters A–E in the correspondence given in Table 4; item positions run from 1 to 7 within each domain, position 7 carrying negative polarity and reverse scoring. Cross-references point to Supplementary Materials S1–S12 and to main-text locations (§, Tables 1–10, Figures 1–4).

2 Part I — Instrument development record

2.1 Conceptual antecedent

Both instruments derive from exploratory item pools developed with clinical input within a prior academic collaboration and published, with their supplements, as a Perspective article. Those pools are the conceptual antecedent of ClinRO-T and ObsRO-P. They were not psychometrically established, and no measurement property is inherited from them.

2.2 Reference version for the development audit

The reference for the item-level audit is the 35+35 candidate version obtained by multidisciplinary re-specification of the antecedent pools (§3.2.1). The audit covers all 70 channel-specific formulations and was completed before enrollment.

2.3 Changes from the reference version

Relative to that version, 25 of the 70 channel-specific formulations were textually modified. In the ClinRO-T, eight formulations were reformulated (C1–C7 and E7). In the ObsRO-P, 17 formulations were changed: ten re-pairings (B1, B2, B3, B6, C1, C2, C3, D1, D2 and D4), three replacements (B4, C5 and D5) and four further reformulations (C4, C6, C7 and E7). The remaining 45 formulations were carried over unchanged.
The revisions were introduced before enrollment and without access to participant data, with the purpose of improving direct observability and cross-context correspondence. Item count and domain structure were preserved, with 35 items per instrument distributed across five domains of seven items. Psychometric equivalence with the antecedent formulations is not assumed.

Change class	ClinRO-T	ObsRO-P	Total
Reformulation	8	4	12
Re-pairing	0	10	10
Replacement	0	3	3

Change class	ClinRO-T	ObsRO-P	Total
Unchanged	27	18	45
Total	35	35	70

Any item revision arising from the modified Delphi procedure or from cognitive interviewing will be recorded in this section before the phase 1 form freeze.

2.4 Development-stage status and evidence location

Development element	Status	Evidence / location
Exploratory antecedent item pools	Completed and published	Antecedent Perspective article and its supplements
Multidisciplinary re-specification into the 35+35 candidate version	Completed before enrollment	§3.2.1; §2.2 above
Item-level development audit of all 70 formulations	Completed before enrollment	§3.2.1; §2.3 above
Channel-specific modifications (n = 25)	Completed without participant data	§3.2.1; §2.3 above
Modified Delphi with independent experts and intended observers	Planned (pre-initiation)	§3.2.1; S1–S2
Cognitive interviewing with intended reporters	Planned (pre-initiation)	§3.2.1; S1–S2, S4
Item revision arising from those procedures	Planned, if indicated	§2.3 above
Phase 1 form freeze	Planned (pre-initiation), after the content-development procedures	S1–S2, S11
Content validity	Not established at this stage	§3.2.1; §3.1 below
Structural validity, responsiveness, measurement error	Deferred (validation study)	S10; §5.2

Elements marked as planned precede the phase 1 form freeze.

3 Part II — Protocol traceability matrix

3.1 Content-development concepts and their limits of transfer

The COSMIN taxonomy and its associated guidance were developed for patient-reported outcome measures. They are used here for content-development concepts only — concept of interest, context of use, item relevance, comprehensiveness, comprehensibility, and cognitive testing with the intended observers — and not as a conformity framework for the present other-report instruments. Reliability, structural validity, responsiveness and measurement error lie outside the pre-implementation stage. Scoring rules, the non-observability taxonomy, aggregation and quality control are recorded in the operational sections below.

3.2 Conceptual framework and construct clarity

- Target domains (COG, SOC, EMO, MOT, SYN) and item polarity, with positions 1–6 positive and position 7 negative — Specified → S1–S2; §2.2, §3.2.2; Table 4.
- Unweighted within-domain aggregation rule, prespecified; no cross-domain composite is computed in phase 1; structural adequacy is not claimed and no causal interpretation is advanced — Specified → §3.2.2, §3.1.10, §4.2, §4.3.
- Scope of the term *neurofunctional*, stated as a level of description without neural indicator — Specified → §1.1, §3.2.2.
- Distinction between extended timeframe and bounded interval — Specified → §3.1.8; S9; Table 8.

3.3 Content-development rationale and planned content-validity procedures

- Contextual relevance of inclusive orchestral therapy as an observation setting rather than as an intervention under evaluation — Specified → §1.2, §3.1.2; S1–S2.
- Cross-context pairing at referent level, context-adapted across the T and P channels — Specified → §2.2, §3.1.9; S1–S2; Table 4.
- Non-observability taxonomy (NO, S-NA, TECH) preventing forced responses — Specified → §3.1.10; S6–S7; Figure 2.
- Modified Delphi procedure and cognitive interviewing — Planned (pre-initiation), before the phase 1 form freeze → §3.2.1; S1–S2.

3.4 Item construction and scoring rules

- Structure of five domains by seven items, with reverse scoring at position 7 — Specified → §2.2, §3.1.10; S5–S7.
- Domain-score availability rule requiring at least five of seven numeric items for a domain mean — Specified → §3.1.10, §4.2; S6–S7; Table 9.
- Completeness represented by N_AVAILABLE_DOMAINS and by domain-specific availability, no cross-domain composite being computed in phase 1 — Specified → S6–S7.
- Grounds prespecified for revising the domain structure before the validation study: near-invariant item distributions, domain-concentrated NO or S-NA, divergent domain-level agreement, and inter-item association inconsistent with the grouping — Specified → §4.2.
- Candidate reconfiguration of domain C, applicable only if those grounds are met, consisting in reassignment of the regulatory items to the cognitive–executive domain and retention of the remaining items as a separate disruption index; no other regrouping is prespecified — Specified → §4.2.

3.5 Further prespecified elements

- Sensitivity summary for domain C retaining a single member of the nested set C2/C6/C7 — Specified → §4.2, §4.3.
- Domain-level cross-context correspondence reported with and without position 7 — Specified → §4.2; S10.

- Agreement reported on the two rater-determined counts, on the derived ordinal band and by denominator class — Specified → §3.1.9, §4.2; S10.
- Reporting of sex, gender and age band following the SAGER guidelines — Specified → §3.1.3; Table 10.
- Convergent analyses against the Vineland-3, restricted to the locked records of Psychologist B — Specified → §3.1.8; S9, S10.
- Provenance of calibration material, consisting of simulated segments without patient or real-session footage — Specified → §3.1.5; S3.
- Calibration concordance between Psychologists A and B, computed on the simulated segments before the first scored session and reported separately from the phase 1 agreement estimates — Specified → §3.1.5; S3, S10.
- Opportunity-identification layer, with realized-opportunity counts drawn from the frozen realized-event log, common to both raters and separate from behavioral rating — Specified → §3.1.6, §3.1.9; S5–S8; Figure 1.
- Item-level classification fixed before data collection, recording opportunity origin (score-determined or endogenous) and principal observational-demand class, with agreement by demand class reported as a secondary descriptive stratum — Specified → §3.1.6, §3.1.9; S5, S10.
- Routing for position 7 through a time-based episode-count band, zero episodes constituting a valid floor score of 1 rather than S-NA — Specified → §3.1.9, §3.1.10; S5–S8; Figure 2.
- Informant replacement handled through INF_ID, INF_RELATION_CLASS and INFORMANT_CHANGE, with a prespecified sensitivity description — Specified → §3.1.9; S4, S6, S10.
- Canonical variable schema, S6 being the single source of truth for variable names, datatypes and ranges — Specified → S6.
- Missing-data handling without imputation at the pre-implementation stage — Specified → S7, S10.

3.6 Measurement properties

- Inter-rater agreement, reported separately at item level (ordinal) and domain level, with participant-level clustering addressed — Planned (phase 1) → S10. Agreement evidence is collected in phase 1 and reliability is not claimed as established.
- Structural validity, not estimable at the planned observation density of 100 records over 35 items clustered within 20 participants — Deferred (validation study) → §4.2, §5.2.
- Responsiveness and measurement error, which the phase 1 design cannot separate from true change — Deferred (validation study) → §4.3. Descriptive within-person variation is planned in phase 1 and does not constitute an estimate of either property → §5.2.

3.7 Instrument definition and rationale

- Definition of both instruments as bounded-interval other-report clinical outcome assessments — Specified → §1.4; S1–S2; Tables 1–2.
- Rationale of bounded-interval, context-specific observation of directly observable behavior — Specified → §1.3, §1.4; S1–S2.

3.8 Administration schedule and timing

- Temporal boundaries: coding deadline of 24 hours or less for the ClinRO-T; 48-hour post-session observation interval for the ObsRO-P, with submission by the end of the interval — Specified → §1.3, §3.1.9; S8; Table 3.
- Completion duration recorded prospectively without a fixed cutoff — Specified → §3.1.9, §4.1; Table 3.

3.9 Mode of administration and training

- Electronic capture through KoBoToolbox/Enketo (ODK), with independent channels and post-submission lock — Specified → §2.1, §3.1.11; S8.
- Rater calibration and caregiver training — Specified → §3.1.5; S3–S4.

3.10 Masking, role segregation and quality control

- Role segregation and masking of Psychologists A and B — Specified → §3.1.7; S3; Table 7.
- Quality-control workflow and data clarification requests — Specified → §3.1.11; S6–S7; Table 9.

3.11 Data handling, privacy and ethics

- Pseudonymization through P_ID and S_ID, audit trail and encryption — Specified → §3.1.12; S11.
- Ethics review, study-specific consent and assent, and the in-session dissent procedure — Planned (pre-initiation) → §3.1.13; S11. Recording and outcome collection begin only once these conditions are met.

3.12 Observation setting

- Inclusive orchestral environment as measurement context — Specified → §1.2, §3.1.2; Table 5.
- Personnel, with music therapists delivering the sessions and Psychologists A and B occupying the rater roles — Specified → §3.1.7; S3.
- Procedural description centred on ObsRO capture rather than on clinical delivery — Specified → §2, §3.1.9.
- Intervention fidelity, dose and session structure are not evaluated, the programme being the context of observation and not the object of study — Not applicable.

3.13 Electronic capture, user acceptance testing and data-integrity controls

- Server-side timestamping (SUB_TS) and post-submission lock — Specified → §2.1, §3.1.11; S8, S11; Table 9.
- Quality-control flags covering timing, response pattern, TECH, domain-score availability, completeness and review trigger — Specified → §3.1.11; S6–S7; Table 9.
- User-acceptance testing of routing, timing capture and logging — Planned (pre-initiation) → S8, S11.

- Append-only data clarification requests with procedural resolution only, excluding rating adjudication, consensus or merging of the two locked ClinRO-T records — Specified → §3.1.11, §3.1.7; S11.
- Role-segregated access and pseudonymization — Specified → §3.1.12; S11.
- Change control and versioning through FORM_VERSION, FORM_DATE and changelog — Specified → S8, S11.

3.14 Non-observability, missingness and aggregation

- Taxonomy of NO, S-NA and TECH implemented in the interface and in the scoring rules — Specified → §3.1.10; S6–S7; Figure 2.
- Exclusion of NO, S-NA and TECH from aggregation, TECH being retained and flagged — Specified → §3.1.10; S6–S7.
- Availability rule of five of seven items for domain scores, with no cross-domain composite in phase 1 — Specified → §3.1.10; S6–S7.

3.15 Pre-implementation outputs and feasibility benchmarks

- Adherence, timing and quality-control summaries, as a planned reporting structure — Specified → §4.1–§4.2; S10.
- Phase 1 agreement and measurement-process plan — Specified → S10.

3.16 Statistical framework

- Descriptive feasibility indicators covering availability, latency and domain-score availability — Specified → S10.
- Phase 1 agreement, measurement-process and exploratory descriptive cross-context analyses, using methods appropriate to ordinal observer ratings and clustered repeated observations — Planned (phase 1) → S10.
- Idiographic time-series and state-space modelling, which five observations per participant do not support and which is therefore not prespecified — Not applicable.
- Attainable score categories, floor and ceiling effects, non-observability, score availability and measurement error, addressed in the sections indicated rather than through analytical detection and quantification limits, which do not transfer to ordinal observer ratings — Specified → §4.2–§4.3; S5, S10.

3.17 Risk and governance

- Pseudonymization and key separation — Specified → §3.1.12; S11.
- Audit trail and export control — Specified → S11.
- Backup, disaster recovery, incident response and breach handling — Specified → S11.
- Readiness of the data protection impact assessment and vendor management — Specified → §3.1.12; S11.

3.18 Version control and change management

- Form freeze before phase 1, subsequent changes requiring approval by the principal investigator and the data controller — Specified → S11.

- Synchronization of Supplementary Materials S1–S12 with the main text — Specified → S11.
- Cross-document consistency check on naming, thresholds and taxonomy, verified against the frozen main text, S1–S12, Tables 1–10 and Figures 1–4 — Specified → S6–S7; Tables 2 and 4 for classification and domains; Tables 9 and 10 for quality control and thresholds.

4 Summary status

Development and protocol elements are prespecified in S1–S12 and in the main text; empirical implementation remains pending. Phase 1 is limited to inter-rater agreement and to the prespecified measurement-process analyses, while domain-structure evaluation, responsiveness and measurement error are deferred to the validation study. No measurement property is established at the present stage, and no psychometric estimate is reported. Governance and data-integrity controls for electronic capture are prespecified; institutional agreement, ethics review, data-protection actions, role appointments and user-acceptance testing remain pre-initiation conditions.

5 Cross-reference index

Main text: §2 (Materials), §3 (Methods), §4 (Anticipated results), Tables 1–10.

Supplementary Materials: S1–S2 (item banks), S3–S4 (training), S5 (observation grid, calibration and reverse scoring), S6–S7 (dictionary and codebook), S8 (interface and windows), S9 (anchoring), S10 (statistical framework), S11 (data management plan). The domain rationale is given in the main text (§3.2.2, §4.2).
